# Resting‐State Electroencephalogram Complexity Is Associated With Oral Ketamine Treatment Response: A Bayesian Analysis of Lempel–Ziv Complexity and Multiscale Entropy

**DOI:** 10.1002/brb3.70166

**Published:** 2024-11-28

**Authors:** Jules S. Mitchell, Toomas E. Anijärv, Adem T. Can, Megan Dutton, Daniel F. Hermens, Jim Lagopoulos

**Affiliations:** ^1^ Thompson Institute University of Sunshine Coast Birtinya Queensland Australia; ^2^ Department of Clinical Sciences Malmö, Faculty of Medicine Clinical Memory Research Unit, Lund University Lund Sweden; ^3^ Thompson Brain & Mind Healthcare Birtinya Queensland Australia

**Keywords:** complexity, electroencephalogram, ketamine, suicidality

## Abstract

**Introduction:**

Subanesthetic doses of ketamine are a promising novel treatment for suicidality; however, the evidence for predictive biomarkers is sparse. Recently, measures of complexity, including Lempel–Ziv complexity (LZC) and multiscale entropy (MSE), have been implicated in ketamine's therapeutic action. We evaluated electroencephalogram (EEG)‐derived LZC and MSE differences between responders and nonresponders to oral ketamine treatment.

**Methods:**

A total of 31 participants received six single, weekly (titrated) doses of oral racemic ketamine (0.5–3 mg/kg) and underwent EEG scans at baseline (Week 0), post‐treatment (Week 6), and follow‐up (Week 10). Resting‐state (eyes closed and open) recordings were processed in EEGLAB, and complexity metrics were extracted using the Neurokit2 package. Participants were designated responders or nonresponders by clinical response (Beck Suicide Scale [BSS] score reduction of ≥ 50% from baseline to the respective timepoint or score ≤ 6) and then compared in terms of complexity across resting‐state conditions and time.

**Results:**

Employing a Bayesian mixed effects model, we found strong evidence that LZC was higher in the eyes‐open compared to eyes‐closed condition, as were MSE scales 1–3. At a global level, responders displayed elevated eyes‐open baseline complexity compared to nonresponders, with these values decreasing from baseline to post‐treatment (Week 6) in responders only. Exploratory analyses revealed that the elevated baseline eyes‐open LZC in responders was spatially localized to the left frontal lobe (F1, AF3, FC1, and F3).

**Conclusion:**

EEG‐complexity metrics may be sensitive biomarkers for evaluating and predicting oral ketamine treatment response, with the left prefrontal cortex bein a possible treatment response region.

## Graphical abstract

EEG‐complexity metrics may be sensitive biomarkers for evaluating and predicting oral‐ketamine treatment response. These findings underscore the value in evaluating both eyes open and closed conditions, and indicate elevated baseline eyes open complexity in the left pre‐frontal cortex may predict a favourable oral‐ketamine treatment response.

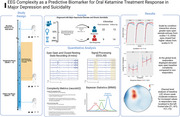

## 1 Introduction

### 1.1 Background

Suicidality, the psychopathological state of experiencing recurrent and intrusive thoughts pertaining to taking one's own life, is a multifaceted and pressing public health concern (Henry 2021; Naghavi 2019). Current pharmacological treatment options are primarily based upon the considerable comorbidity with major depressive disorder (MDD), bipolar disorder (BPD), and post‐traumatic stress disorder (PTSD) (Cai et al. 2021; Dome et al. 2019; Fox et al. 2021). These treatments, which include antidepressants (e.g., fluoxetine and venlaflaxine), mood stabilizers (e.g., lithium), antipsychotics (e.g., clozapine), and neurostimulation (e.g., electroconvulsive therapy), have limited efficacy (Cipriani et al. 2013; Fink, Kellner, and McCall 2014; Gibbons et al. 2012; Masdrakis and Baldwin 2023; Wilkinson et al. 2022). Furthermore, the time to efficacy can be protracted and nonlinear, and in some individuals the therapeutic effects are attenuated or nonexistent (Lopez‐Castroman et al. 2016). In part, this variability in response may reflect the disconnection between treatment mechanism and the neurobiological substructure of suicidality, for which there remains ambiguity (Can et al. 2023).

### 1.2 Ketamine and Glutamatergic Dysfunction

The role of glutamatergic dysfunction in suicidality has recently gained traction (Canet al. 2023); a revision primarily borne out of the significant and rapid responses to low‐dose (0.5–3.0 mg/kg) ketamine and the associated modulation of excitatory and inhibitory signaling and connectivity (Abdallah et al. 2018; Colla et al. 2021). Whilst there has been limited investigation of ketamine's treatment efficacy in suicidality specifically (Can et al. 2021), reduced suicidal symptoms have been observed in studies of MDD (Ionescu et al. 2019), BPD (McIntyre et al. 2020), and PTSD (Feder et al. 2014). Replicating and extending upon preclinical findings (Chowdhury et al. 2012; Shinohara et al. 2021), human studies show that low doses alter metabolic cycling dynamics (Abdallah et al. 2018 ) and the balance of excitatory and inhibitory signaling (Li et al. 2017; Milak et al. 2020). Increasing preclinical evidence indicates ketamine's inhibition of NMDA receptors on inhibitory neuronal populations may play an integral role in the disinhibition of cortical pyramidal neurons (Ali et al. 2020; Cichon et al. 2023; Gerhard et al. 2020), which instigates a surge in glutamatergic signaling and bottom‐up prediction error gain. At a system level, this drives a reconfiguration of neural network connections within and between psychopathology‐related regions (Li et al. 2020; Muthukumaraswamy et al. 2015). Importantly, ketamine‐mediated NMDA receptor hypofunction and global network reconfiguration may deprecate existing and entrenched internal (predictive) models that influence perceptual inference and decision‐making certainty (Salvador et al. 2022; Weber et al. 2020).

### 1.3 Ketamine and Entropy‐Mediated Plasticity

Until recently, the unification of ketamine's neurobiological mechanisms and its treatment‐associated psychological epiphenomena within a cogent theoretical framework has been lacking. Initially formulated with respect to the psilocybin literature, we recognize the “entropic brain” hypothesis (Carhart‐Harris et al. 2014), and its integration in the model of canalization and temperature and entropy‐mediated plasticity (TEMP; Carhart‐Harris et al. 2023) holds considerable value as a parsimonious model of psychopathology with verifiable treatment implications. We argue this can (and should) be extended to suicidality and ketamine's therapeutic action. As outlined in their exposition (Carhart‐Harris et al. 2023), to which we refer the reader seeking an extensive discussion, psychopathology may in fact reflect a hyper‐refined and entrenched (canalized) brain state incapable of, or at least resistant to, revision. Moreover, they outline the value inherent to classical psychedelics (i.e., psilocybin) is the capacity to pharmacologically induce changes in the brain's state space via TEMP, or more generally the “entropic brain” effect, which is characterized by increased complexity or richness of neuronal communication.

In contrast to psilocybin's (primarily) serotonergic action (Carhart‐Harris et al. 2023), ketamine alters excitatory and inhibitory neuronal metabolism and signaling dynamics via (primarily) noncompetitive NMDA receptor antagonism (Gerhard et al. 2020; Shinohara et al. 2021). Despite differing in its mechanisms of action, there is growing evidence to indicate that ketamine similarly induces an acute state of TEMP. In humans, ketamine has been shown to decrease within‐ and increase between‐network connectivity (Demchenko et al. 2022), and increase the irregularity/unpredictability (broadly referred to as complexity or entropy) of neuronal firing dynamics derived from magnetoencephalogram (MEG)/electroencephalogram (EEG) data (De La Salle et al. 2016; Farnes et al. 2020; Luppi et al. 2023; Schartner et al. 2017). Critically, the effects on complexity may be temporally dependent, with a recent report in military veterans with treatment‐resistance depression (TRD) finding a single intravenous dose of ketamine (0.1, 0.25, or 0.5 mg/kg) increased (compared to baseline) neural complexity (Lempel–Ziv complexity [LZC] and multiscale entropy [MSE]) at 30 minutes post‐infusion, but decreased it at 24‐hours (Murphy et al. 2023).

The capacity for ketamine to alter neural complexity dynamics is of particular relevance, as nonlinear complexity indices derived from EEG have been associated with MDD severity (Torre Luque and Bornas 2017), and diagnosis (Bachmann et al. 2018; de Aguiar Neto and Rosa 2019; Yang et al. 2023). Furthermore, complexity indices have been associated with treatment response to both antidepressants and transcranial magnetic stimulation (Jaworska et al. 2017; Lebiecka et al. 2018; Méndez et al. 2012; Shalbaf et al. 2018; Thomasson et al. 2000). Collectively, these findings indicate methods for estimating neural complexity (reviewed by Lau et al. 2022) may provide a biomarker for predicting and evaluating treatment‐response trajectories to ketamine.

### 1.4 Rationale, Aims, and Hypotheses

We previously reported on the safety, tolerability, and change in suicidal symptoms following a 6‐week open‐label trial of low‐dose (0.5–3.0 mg/kg) oral ketamine (racemic) treatment delivered once per week (Can et al. 2021). Previous secondary analyses have shown treatment‐associated changes in hippocampal structural volume (Dutton et al. 2024), functional connectivity (Can, Hermens, et al. 2023), resting (Anijärv et al. 2023), and auditory‐evoked centro‐parietal band power (Can, Schwenn, et al. 2023).

To date, there has been no investigation of oral ketamine's effects on complexity dynamics, nor an evaluation of protracted changes in tsuch indices between responders and nonresponders to oral ketamine treatment. Furthermore, no previous investigations have employed Bayesian analyses for the estimation and comparison of complexity values. Given that a central interest of such research is in quantifying parameter estimates, and where there is evidence of a difference with treatment and/or across groups, a Bayesian statistical approach is warranted, particularly considering the Bayesian credible intervals permit what confidence intervals cannot; a range within which the parameter has a given (e.g. 95%) probability of being in (Makowski et al. 2019).

The current study investigated changes in resting‐state eyes‐open and eyes‐closed complexity following a 6‐week open‐label trial of low‐dose oral ketamine (racemic) treatment, and at 4‐week follow‐up. Specifically, this secondary analysis examined the treatment‐associated changes in LZC and MSE in treatment responders versus nonresponders. Employing a Bayesian mixed effects model, it was hypothesised that treatment responders would have higher neural complexity at baseline compared to nonresponders. Second, it was hypothesized that evidence for a response status by time interaction would be found, such that only treatment responders would show robust evidence of a decrease in neural complexity from baseline to posttreatment (Week 6). These predictions were informed by previous studies showing complexity decreased with antidepressant treatment, and that this reduction correlated with the degree of symptom remission (Méndez et al. 2012; Thomasson et al. 2000). Third, we anticipated a regression to baseline levels from post‐treatment to follow‐up, similar to previous investigations of this open‐label trial. Finally, it was predicted that complexity indices would be greater in the eyes‐open condition compared to the eyes‐open condition, as observed in previous studies (Lord and Allen 2023; Vecchio et al. 2021; Yang et al. 2023).

## 2 Materials and Methods

### 2.1 Study Design

The Oral Ketamine Trial on Suicidality (OKTOS) was undertaken at the Thompson Institute, University of the Sunshine Coast (UniSC), Australia, between August 2018 and November 2019 (Can et al. 2021). The trial recruited participants with chronic suicidality (i.e., experiencing suicidal ideation of varying intensity over months to years) as determined by the trial psychiatrist, and who had a baseline Beck Suicide Scale (BSS) score ≥ 6. Additional relevant inclusion criteria included the participants not previously being users of ketamine (clinically or recreationally) nor having a history of psychotic disorders.

Participants were administered subanaesthetic doses of racemic oral ketamine once per week for 6 weeks (under direct supervision of the trial psychiatrist). Dosing commenced at 0.5 mg/kg and was subsequently titrated at successive weeks according to participant response and tolerability, with increments of up to 0.5 mg/kg per week. Participant response and tolerability were assessed by clinical evaluation of physical and psychotomimetic side effects. For the interested reader, the by‐participant dosing schedule has been described previously (Can et al. 2023). Upon treatment cessation (Week 6) and at follow‐up (Week 10), responders were classified as those showing a BSS score reduction of ≥ 50% from baseline to the respective timepoint or having a BSS score ≤ 6 (see Table 1).

**TABLE 1:** Timepoint‐by‐condition breakdown of key demographic and electroencephalogram (EEG) parameters.

Demographics									
Timepoint	Group			Mean age (SD)			Sample size (% Female)		
Baseline/posttreatment	Responder			42.6 (13.8)			22 (50)		
	Nonresponder			51.4 (13.5)			9 (55)		
Follow‐up	Responder			48.3 (15.0)			13 (38)		
	Nonresponder			42.0 (13.7)			14 (71)		
EEG Parameters									
Timepoint	Baseline			Posttreatment			Follow‐up		
Condition type	EC	EO	Total	EC	EO	Total	EC	EO	Total
Recording number	30	28	58	29	30	59	27	26	53
Recording duration analyzed (% of total)	238.54 (99.4)	239.23 (99.7)	—	238.92 (99.59)	237.05 (99.08)	—	239.29 (99.74)	238.47 (99.40)	—
Total data points	59,634	59,808	—	59,730	59,427		59,824	59,618	—
Median EEG recording time (minimum/maximum)	1:21 p.m. (10:59 a.m./5:15 p.m.)			12:09 p.m. (09:55 a.m./5:52 p.m.)			12:35 p.m. (10:33 a.m./5:22 p.m.)		

Abbreviations: EC, eyes closed; EEG, electroencephalogram; EO, eyes open; Max, maximum; Min, minimum; NR, nonresponder; Resp, responder; SD, standard deviation.

### 2.2 Participants

All participants provided informed written consent. The trial was registered with the Australian Clinical Trials Registry (ACTRN12618001412224), approved by Bellberry Human Rights Research Ethics Committee (approval number: 2017–12‐982), and ratified by the UniSC Human Research Ethics Committee (A181101).

A total of 31 OKTOS participants were included in this secondary analysis. Approximately equal proportions of males and females were included in the current analyses (54% female), with a mean age (SD) of 45.64 (13.95) years. Concurrent psychopharmacological agents included antidepressants (e.g., SSRIs and SNRIs), antipsychotics, and mood stabilizers (see Can et al. 2023 for a full list). Complete resting‐state EEG recordings were acquired for 22 participants (all timepoints and conditions [i.e., eyes open and closed]). A total of nine participants were missing at least one EEG timepoint or condition type, with the by‐timepoint breakdown being: baseline, two (ID 6 and 31) missing eyes open and one (ID 24) missing both conditions; posttreatment, one (ID 1) was missing eyes closed and one (ID 21) missing both conditions; and follow‐up, one (ID 6) missing eyes open and four (ID 14, 19, 34, and 39) missing both conditions.

### 2.3 Electroencephalogram (EEG)

#### 2.3.1 Acquisition

EEG was acquired in a light‐ and temperature‐controlled room using a Biosemi ActiveTwo 32‐channel system (Biosemi B⋅V, Amsterdam, Netherlands) at a sampling rate of 1024 Hz. Scalp electrodes (Ag/AgCl active electrodes impedances < 40 kΩ) were localized according to the International 10–20 standardized system, with additional vertical and horizontal electrodes to record electrooculograms (EOGs) used to monitor for eye movements.

#### 2.3.2 Preprocessing

Data were processed offline using the EEGLAB software (version 2023.0) with custom MATLAB scripts for semi‐automated preprocessing (see GitHub link). First, non‐EEG electrodes (EOGs) were removed, and each recording was cut to include only the 4‐min resting‐state recording. Data were subsequently down‐sampled from 1028 to 250 Hz and re‐referenced from Cz to the average of all 32 channels using the *fullRankAvg* function in EEGLAB (retaining full rank of the data [i.e., 32]).

A 1 Hz (passband lower edge; 0.5 Hz [−6 dB] cutoff frequency) high‐pass FIR filter (zero‐phase, non‐causal, hamming window) was applied before 50 Hz line noise (and its harmonics) was removed using the *Zapline* and *Cleanline* plugins. The combination of *Zapline's* stationary spatial filter approach and *Cleanline's* nonstationary time‐domain approach produces superior results (Miyakoshi et al. 2021). A 50 Hz (passband upper edge; 56.25 Hz [−6 dB] cutoff frequency) high low‐pass FIR filter (zero‐phase, non‐causal, hamming window) was subsequently applied.

The down‐sampled and filtered data were subsequently submitted for independent component analysis (ICA) using the “runica” method. Using ICLabel, components were automatically labeled based on the scalp topographies, time series, activity and spectral profiles. Eye, muscle, heart, line, and channel noise were flagged and removed from the data if labeled with 80% or greater confidence. The data were again re‐referenced to average.

Following artifactual independent component removal, the *clean_rawdata* plugin was applied with artifact subspace reconstruction. Bad channels were defined as those with scalp‐wide correlation coefficients of < 0.80, which were subsequently reconstructed using spherical spline interpolation. Similarly, reconstruction was applied to burst, flat‐line, and trending artifacts exceeding parameter thresholds. Finally, the surface Laplacian (spline method) was applied to further attenuate low‐frequency spatial artifacts. The cleaned resting‐state eyes‐closed and eyes‐open data were exported (.set file format) for subsequent Python data formatting and analysis using MNE‐Python (Gramfort et al. 2013) and Neurokit2 (Makowski et al. 2021).

#### 2.3.3 Lempel–Ziv Complexity

LZC provides an index of the compressibility of a binarized time series of finite length based on the number of unique sequences (patterns), where “complexity” is the inverse of compressibility. Formally, the LZC algorithm scans the binarized time series, creating a dictionary of unique patterns and increasing the LZC count for each unique pattern (Lempel and Ziv 1976).

We estimated LZC using the Neurokit2 function (Makowski et al. 2021). The median preprocessed recording duration across participants and timepoints was 239s. Each recording was split into nonoverlapping 5 s epochs (47 total), with 1250 samples (250 Hz × 5 s) per epoch. The data were binarized according to whether an individual value was greater than (1) or less than (0) the median of epoch's amplitude. LZC was calculated by epoch for each channel, with the mean across channels forming the global value. Note, the Neurokit2 algorithm scales the LZC value by the logarithm of the sequence length and then divides it by the length of the sequence.

#### 2.3.4 Multiscale Entropy

MSE provides an estimate of a time series signals complexity across different timescales to account for the temporal variability in cortical processes (Costa et al. 2002, 2005,2002, 2005). MSE is an extension of sample entropy, applying a coarse‐graining (down‐sampling) procedure to the original time series data (Scale 1) before calculating sample entropy at each timescale. For example, at Scale 2 the time series is down‐sampled by successively averaging two consecutive timepoints (*Y*_1_ = *X*_1_ + *X*_2_/2, *Y*_2_ … *Y_N_*), for the length (*N*) of the time series. The complete mathematical rationale for MSE and its functional interpretation in biological signals is described in Costa et al. (2002, 2005,2002, 2005).

For the current study, MSE was estimated on the same 5‐s epochs extracted for the LZC analysis. Subsequently, averaging was performed across epochs and channels to provide a single MSE value per scale. Contrary to previous methodologies (Murphy et al. 2023), MSE was unable to be calculated for 20 scale factors due to unstable estimates. Stable MSE estimates were obtained with 10 scale factors (timescale range of 4–40 ms), with an embedding dimension of 2 and tolerance set to the packages default (0.2 × standard deviation).

### 2.4 Statistical Analysis

#### 2.4.1 Mixed Effects Model

Primary analyses were conducted using the Bayesian Regression Models using the STAN (*BRMS*) R package (Bürkner 2017) with custom scripts (see GitHub link). The *BRMS* package supports fitting Bayesian (non)linear multivariate multilevel models using the Hamilton Monte Carlo variant No‐U‐Turn Sampler (NUTS) implemented in STAN, a probabilistic programming language for Bayesian model specification and inference (Carpenter et al. 2017). Bayesian mixed effects models were fit using the *BRMS* package (version 2.20.4), with data organization, summaries, plotting, and post hoc comparisons supported by the *ggplot2*, *tidybayes*, *bayesplot*, *bayestestR*, *emmeans, tidyverse*, and *xlsx* packages.

Separate models were specified and fit for the MSE and LZC. For each final model, population‐level (fixed) effects of timepoint (baseline, posttreatment, and follow‐up), response status (nonresponder and responder), condition (eyes closed and eyes open), and their interaction were included. A multivariate model was utilized for the MSE analysis, with the scale values from 1 to 10 specified as the dependent variables (all varying effects of subject across the response variables [scales 1–10] were modeled as correlated [specified by |p|]).

For each dependent variable, the distribution was truncated at 0 in accordance with values being real‐valued positives. Both final models included by‐participant random intercepts and slopes for population‐level effects, as defined below:

$$\begin{array}{ccc} & & \mathrm{brm}(\mathrm{LZC}\;|\mathrm{res}p_{\mathrm{trunc}}\left(\mathrm{lb}=0\right)\;∼\;1+\mathrm{Time}\mathrm{point}\;\times \;\mathrm{Resp}\mathrm{onder}\\ & & \;\times \;\mathrm{Task}+\left(1+\mathrm{Time}\mathrm{point}+\mathrm{Resp}\mathrm{onder}+\mathrm{Task}\;|p|\;\mathrm{Subj}\mathrm{ect}\right) \end{array}$$

$$\begin{array}{ccc} & & \mathrm{brm}(\;\mathrm{bf}(\mathrm{mvbi}\mathrm{nd}(\mathrm{Scale}\;1,\;\mathrm{Scale}\;2,\cdots \mathrm{Scale}\;10|\mathrm{res}p_{\mathrm{trunc}}\left(\mathrm{lb}=0\right)\\ & & ∼\;1+\mathrm{Time}\mathrm{point}\;\times \;\mathrm{Resp}\mathrm{onder}\;\times \;\mathrm{Task}\;\times \;\mathrm{Scale}\\ & & +\left(1+\mathrm{Time}\mathrm{point}+\mathrm{Resp}\mathrm{onder}+\mathrm{Task}\;|p|\;\mathrm{Subj}\mathrm{ect}\right) \end{array}$$

The following model specifications were set: family = gaussian, chains = 4, thinning = 1, iterations = 15,000 (warm‐up = 7500). Thus, after warm‐up a total of 30,000 posterior samples were obtained ([15,000 − 7500] × 4).

##### 2.4.1.1 Prior Specification

Prior distributions on model parameters must be specified for Bayesian analyses. Informed by (1) logical bounds, (2) previously reported mean and standard deviation (SD), and (3) prior predictive checks, regularizing‐principled priors were specified (see Supporting Information A: Priors, Table S1). Note, in BRMS, the prior on the intercept corresponds to the value when all factors are set to the first level; this equates to nonresponders at baseline for the eyes‐closed condition. For the LZC models, a normal (mean = 0.5, SD = 0.1) prior was implemented for the intercept, consistent with previous reports of LZC values in MDD being around 0.5 (Murphy et al. 2023; Yang et al. 2023). For the MSE model, a normal (mean = 1.2, SD = 0.2) prior was implemented for the intercept across scales; in the absence of comparable previous literature, this allocated credibility to values spanning 0.6–1.8. In each model, a normal (mean = 0, SD = 0.1) prior was specified for each beta coefficient, predicting changes to be positive or negative, with most probability centered on zero effect.

##### 2.4.1.2 Model Comparison

To reduce run time, the following parameter modifications were made for model comparison: chains = 2, iterations = 2000 (warm‐up = 1000). Model fit was compared using the leave‐one‐out (LOO) cross‐validation and “bayes_R2” functions from the *BRMS* package. The aforementioned mixed models provided the best fit for the LZC and MSE data (see Supporting Information B: Model Comparison, Tables S2–S5).

##### 2.4.1.3 Prior Predictive Checks, Convergence, and Posterior Checks

Prior predictive checks for the final MSE and LZC models were conducted to validate the priorpredicted reasonable values (i.e., ≥ 0; see Supporting Information C: Prior Predictive Checks, Figures S1 and S2). Model convergence and performance were assessed using trace plots, R‐hat values (< 1.01), autocorrelation, error distributions, observed versus predicted values, and effective sample size for the posterior distributions (see Supporting Information D: Model Performance and Convergence, Figures S3a–S14b). Posterior predictive checks were performed to evaluate the correspondence between the data's minimum, maximum, and mean values and those predicted from the posterior distribution (Supporting Information E: Posterior Predictive Checks, Figures S15–S19f).

##### 2.4.1.4 Posterior Distribution Effect Estimates

Summarized median fixed and random effect estimates (95% credible intervals) are reported in text (see Supporting Information F: Effect Estimates, Table S8). Note again, the intercept reported refers to nonresponders at baseline for the eyes‐closed condition. From this, beta coefficients are calculated. To evaluate the “significance” of each parameter's beta coefficient estimate and to provide a bridge between frequentist and Bayesian approaches, the probability of direction is reported (Makowski et al. 2019). Importantly, this statistic has a 1:1 correspondence with the frequentist *p*‐values, being interpreted as the probability (defined by the posterior distribution) that the estimate is of the median estimates sign (i.e., the (un)certainty with which an effect goes in a particular direction). To supplement this approach, frequentist statistics are reported in the Supporting Information (see Supporting Information G: Frequentist Statistics, Tables S11–S13).

##### 2.4.1.5 Post Hoc Comparisons

Main effects and interactions indicated by beta coefficient estimates and the probability of direction were followed up with post hoc comparisons of marginal means using the *emmeans* package (Searle et al. 1980). Using estimated marginal means and 95% highest posterior density (HPD), the evidence for differences between factor levels was evaluated (see Supporting Information H: Estimated Marginal Means, Tables S14–S17). The HPD characterizes the uncertainty of the estimation, whereby the interval comprises 95% of the distribution's density mass; the range of most credible parameter estimates (Kruschke and Liddell 2018).

#### 2.4.2 Channel‐Level Comparisons

Bayesian paired and independent sample (student) *t*‐tests were performed using the JASP software (version 0.17.2.1). Differences in by‐channel resting‐state LZC values across conditions and responders versus nonresponders were assessed (full output in Supporting Information I: JASP Output, Tables S18 and S19). The default JASP prior (Cauchy = 0.707) was used, and the number of posterior samples was set to 1000. Bayes factors (BF_10_) and sampling errors (%) are reported, with the BF_10_ representing the likelihood of the alternative hypothesis given the data. The likelihood of the null hypothesis given the data is given by dividing 1 by the BF_10_. For all tests, the null hypothesis being tested was the equality of the two groups (response status). The qualitative grading of evidence (BF_10_) follows JASP recommendations: < 3 = weak evidence; ≥ 3 = moderate evidence; ≥ 10 = strong evidence; ≥ 30 = decisive evidence. Topographical plots of channel‐level LZC values and BF_10_ were created using the *ggplot2* R package.

## 3 Results

### 3.1 Data Overview

A total of 31 participants were included in each mixed effects model, with a total of 170 observations. Referring to Table 1, note that (1) the reduction in the sample size of analyzed data at follow‐up was due to missing data (*n* = 4) and (2) the change in mean age and percentage of females in each group was due to the combination of missing data and the change in group membership at follow‐up. The number of eyes closed and eyes open recordings and sex proportions were largely consistent across timepoints (Table 1), as were the recording durations. To verify that the time of EEG recording did not systematically influence responder versus nonresponder complexity, we conducted additional mixed effects models with recording time (pre‐ or post‐12 p.m.) as a fixed effect. While evidence of a difference in baseline eyes‐closed MSE scale 1 values between responders and nonresponders was found, there was no evidence of systematic differences (see Supporting Information J: Time of Day, Figures 20a–21b). To verify that the EEG recordings were not confounded by drowsiness, we visually checked the spectral plots for the first and second halves of the 4‐min recording period for a quality control sample (10%). No systematic differences indicative of drowsiness (increased slow wave activity, alpha blocking, etc.), and this was confirmed with EEG recording session runsheets.

Each model displayed good within‐ and between‐chain convergences (trace plots, R‐hat values [< 1.01]), with acceptable autocorrelation and prior predicted values within a logical range. Furthermore, errors were normally distributed, observed versus predicted values followed a linear distribution, and the effective sample size (number of independent draws) in the bulk and tail was high, suggesting the model produced stable and robust estimates (see Supporting Information E: Model Performance and Convergence, Figures S3a–S14b).

### 3.2 Global Analysis

#### 3.2.1 Multiscale Entropy

**FIGURE 1:**
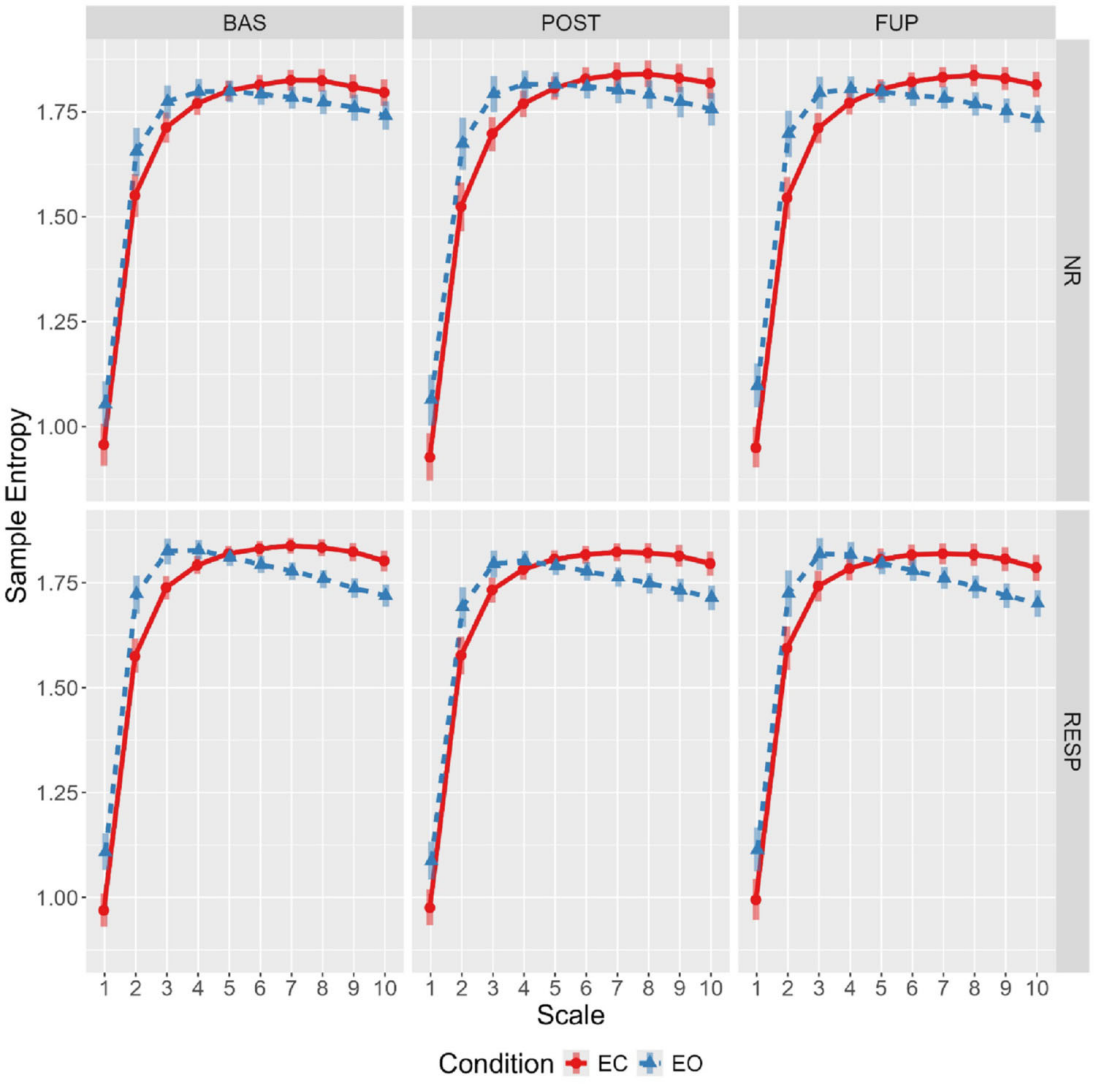
Estimated marginal means for multiscale entropy showing condition‐by‐scale interaction across timepoint and response status. Bars represent 95% highest posterior density. BAS, baseline; EC, eyes closed; EO, eyes open; FUP, follow‐up; NR, nonresponder; POST, posttreatment; RESP, responder.

The full effect estimates are reported in the Supporting Information (see Supporting Information F: Effect Estimates, Table S9). Evidence of a condition‐by‐scale interaction was found, with eyes‐open sample entropy values being higher across scales 1 to 4, whereas the eyes‐closed condition showed higher values from scales 6 to 10 (Figure 1; see Supporting Information F: Effect Estimates, Table S9 and Supporting Information H: Marginal Means, Table S15).

**FIGURE 2:**
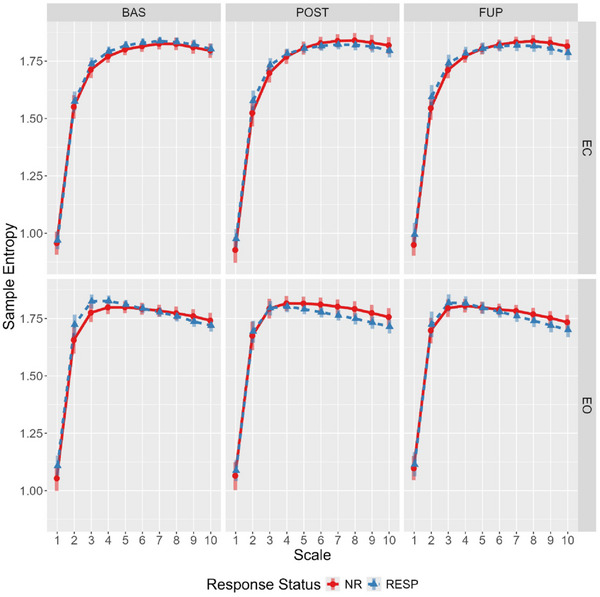
Estimated marginal means for multiscale entropy showing response‐by‐scale interaction across timepoint and condition. Bars represent 95% highest posterior density. BAS, baseline; EC, eyes closed; EO, eyes open; FUP, follow‐up; NR, nonresponder; POST, posttreatment; RESP, responder.

At baseline, responders showed marginally higher eyes‐open sample entropy for scales 1–4 compared to nonresponders, and these differences were attenuated at post‐treatment and follow‐up (Figure 2). Specifically, this attenuation was primarily driven by reduction in eyes‐open sample entropy for responders from baseline to post‐treatment (effect estimate probability of directions ranged from 97.5% to 99%), with some evidence for the opposite effect in nonresponders (effect estimate probability of directions ranged from 92% to 97.2%).

#### 3.2.2 Lempel–Ziv Complexity

**FIGURE 3:**
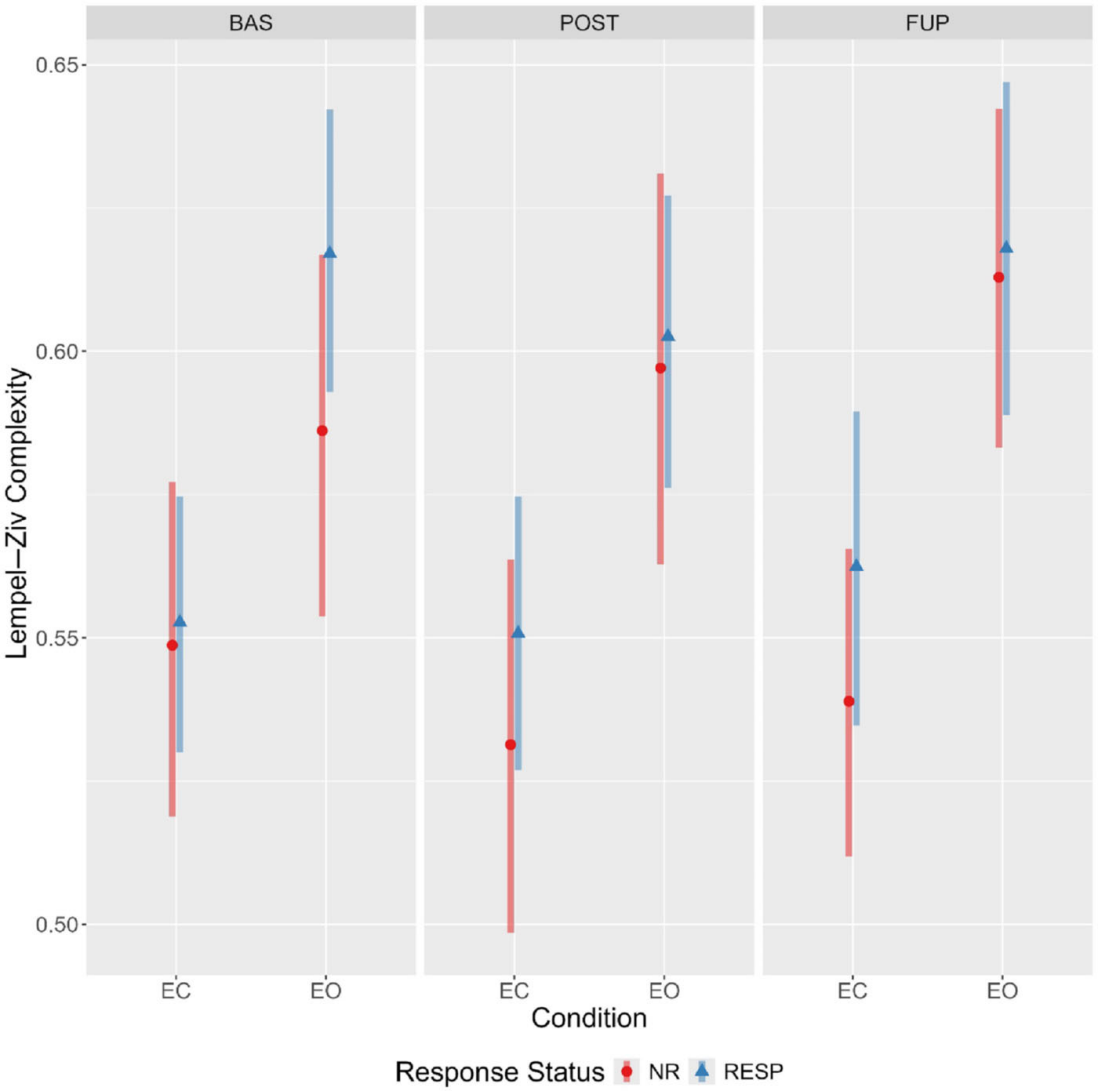
Estimated marginal means for Lempel–Ziv complexity demonstrating condition‐by‐timepoint interaction across response status. Bars represent 95% highest posterior densities. BAS, baseline; EC, eyes closed; EO, eyes open; FUP, follow‐up; NR, nonresponder; POST, posttreatment; RESP, responder.

**FIGURE 4:**
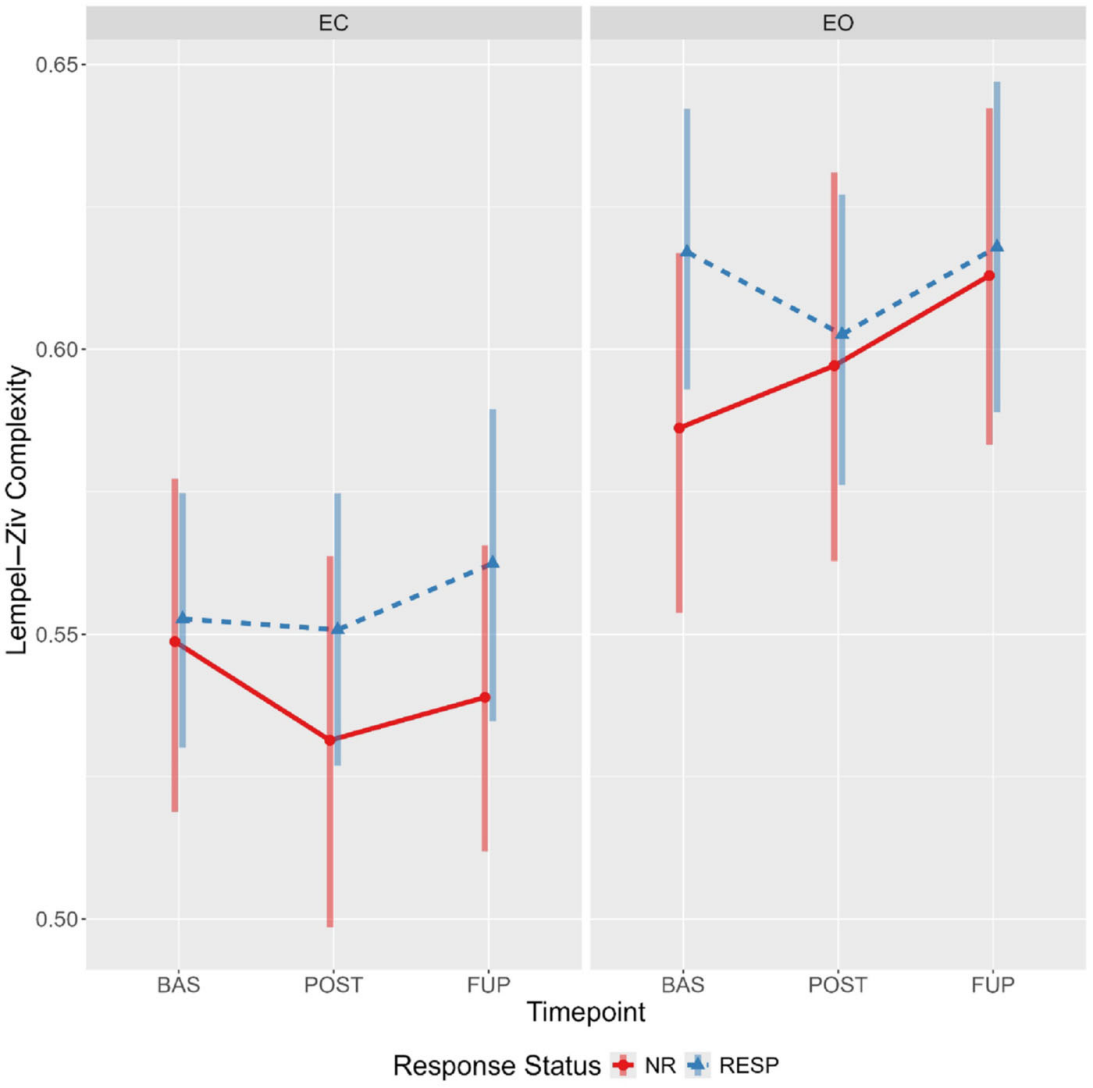
Estimated marginal means for Lempel–Ziv complexity showing timepoint‐by‐condition‐by‐response status interaction. Bars represent 95% highest posterior densities. BAS, baseline; EC, eyes closed; EO, eyes open; FUP, follow‐up; NR, nonresponder; POST, posttreatment; RESP, responder.

Evidence for a main effect of condition was observed, with LZC values being considerably higher during the eyes‐open condition irrespective of response status or timepoint (Figure 3). Furthermore, there was reasonable evidence for a time‐by‐condition‐by‐responder interaction (Table 2). Specifically, responders had somewhat elevated eyes‐open LZC at baseline compared to nonresponders (94.9% of the effect estimate distribution being positive). The effect estimates (and probability of direction), coupled with the estimated marginal means (Figure 4; see Supporting Information G: Marginal Means, Table S14) indicate a decrease in eyes open LZC from baseline to post‐treatment for responders (effect estimate probability of direction = 97%), but the opposite pattern for nonresponders (effect estimate probability of direction = 95%). An almost identical, but mirrored, pattern was observed in the eyes‐closed condition, with responders showing increasingly greater LZC compared to nonresponders from baseline to follow‐up (Figure 4).

**TABLE 2:** Posterior distribution median effect estimates for Lempel–Ziv complexity.

Parameter	Effect estimate (95% CI)	PD
Nonresponder (intercept)	0.5487 [0.5192, 0.5777]	1
Responder	0.0041 [−0.0265, 0.0348]	0.6052
Posttreatment	−0.0174 [−0.0445, 0.01]	0.8989
Follow‐up	−0.0097 [−0.038, 0.0186]	0.757
Eyes open	0.0372 [0.0095, 0.0652]	0.9954
Responder: posttreatment	0.0155 [−0.0175, 0.0479]	0.827
Responder: follow‐up	0.0195 [−0.0181, 0.0569]	0.8486
Responder: eyes open	0.0273 [−0.0056, 0.0593]	0.9495
Posttreatment: eyes open	0.0286 [−0.0059, 0.0622]	0.9507
Follow‐up: eyes open	0.0367 [0.0037, 0.0692]	0.9849
Responder: posttreatment: eyes open	−0.0414 [−0.0809, 0.0009]	0.9728
Responder: follow‐up: eyes open	−0.0453 [−0.0889, −0.0018]	0.9798

Abbreviations: 95% CI, 95% credible intervals; PD, probability of direction.

### 3.3 Channel Analysis

In light of the above findings, a post‐hoc exploratory analysis of baseline LZC values across channels was conducted to evaluate whether the condition and response status differences were localised to particular electrodes.

#### 3.3.1 Condition

A Bayesian paired‐sample *t‐*test across resting conditions revealed that the observed global LZC difference was largely spatially conserved at the channel level. Strong‐to‐decisive evidence for increased LZC in the eyes‐open condition was present at 18 of the 32 channels, with only four channels having less than moderate evidence of a difference (Supporting Information I: JASP Output, Table S18). Notably, the magnitude of LZC values varied considerably across channels within and between conditions, with the largest differences situated in the anterior‐lateral (frontal lobe) and parietal‐occipital regions.

#### 3.3.2 Response Status

**FIGURE 5:**
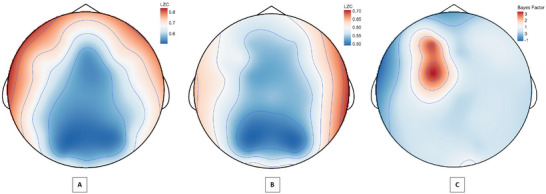
Topographical plot of by‐channel eyes‐open Lempel–Ziv complexity values at baseline for (A) responders, (B) nonresponders, and (C) the evidence (Bayes factors) for a difference between the groups.

A Bayesian independent samples *t*‐test of LZC values in responders versus nonresponders indicated anecdotal to moderate evidence for differences in the eyes‐open conditions at channels Fp1, AF3, F3, and FC1 channels (see Figure 5). No evidence of channel differences was found for the eyes‐closed condition (Supporting Information I: JASP Output, Table S19).

#### 3.3.3 Mixed Effects Model

Considering the LZC differences between responders and nonresponders were localised to four channels (Fp1, AF3, F3, and FC1), an additional (exploratory) multivariate Bayesian mixed effects model of the eyes‐open data was conducted (see Supporting Information: By‐Channel Model, Tables S6 and S7 for prior specification and model checks). We anticipated a similar trajectory to the global analysis, with responders showing a complexity reduction from baseline to post‐treatment, and the differences in complexity values between groups attenuating following treatment.

**FIGURE 6:**
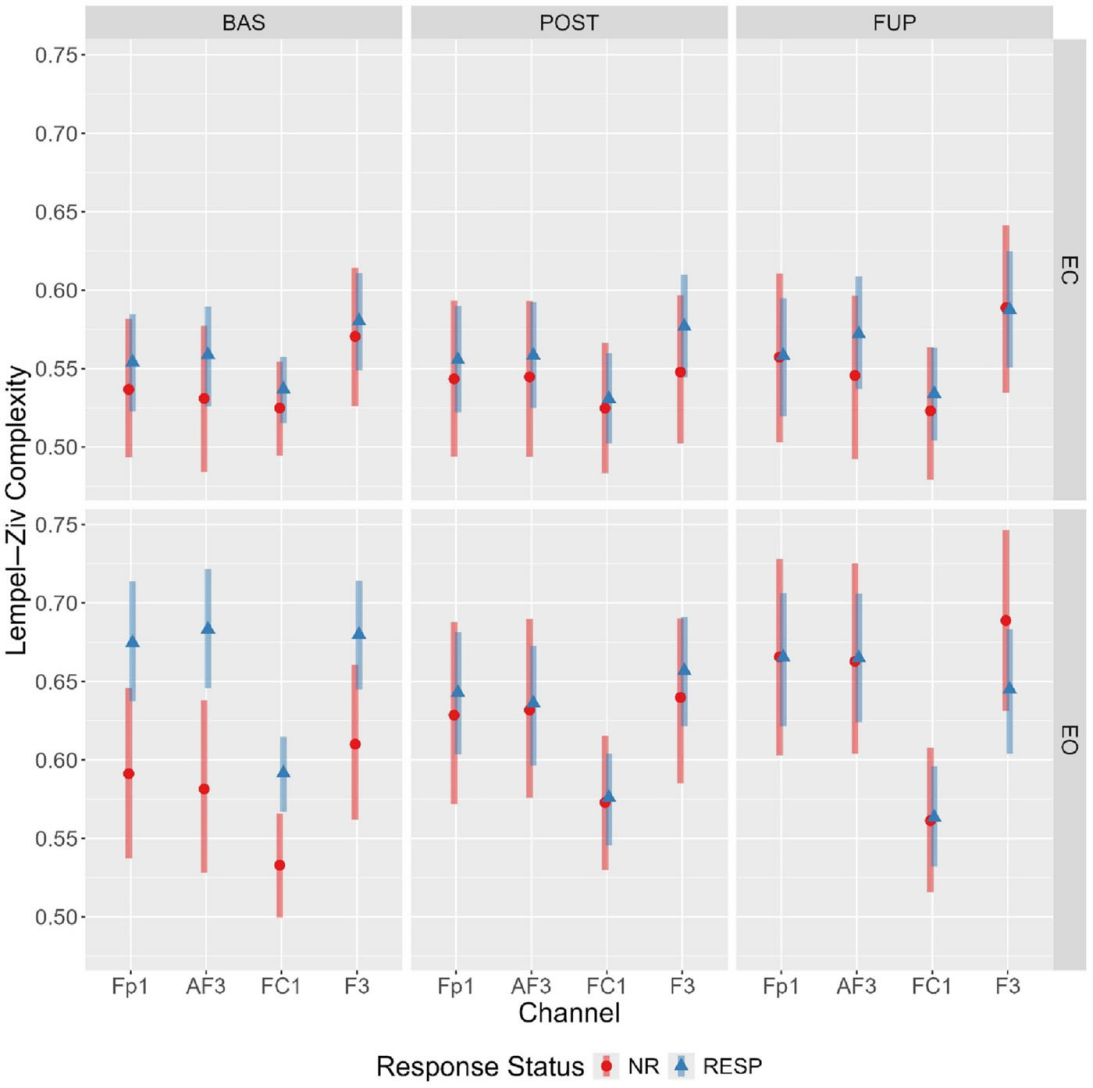
Estimated marginal means for Lempel–Ziv complexity at channels Fp1, AF3, FC1, and F3, showing timepoint‐by‐condition‐response status interaction. Bars represent 95% highest posterior density. AF3, anterior‐frontal 3; BAS, baseline; EC, eyes closed; EO, eyes open; F3, frontal 3; FC1, fronto‐central 1; Fp1, frontal pole 1; FUP, follow‐up; NR, nonresponder; POST, posttreatment; RESP, responder.

Mirroring the global LZC analysis, the model provided evidence for a main effect of condition and a timepoint‐by‐condition‐by‐responder interaction for each electrode (see Supporting Information B, F, G, and H for outputs). A comparison of estimated marginal means showed that eyes‐open LZC values across each of the four channels were elevated in responders at baseline (with minimal overlap in 95% HPD). In responders, these levels decreased from baseline to post‐treatment, yet increased in nonresponders (Figure 6), with the distributions overlapping at post‐treatment and follow‐up.

## 4 Discussion

This secondary analysis of OKTOS (Can et al. 2021) examined two nonlinear complexity metrics, LZC and MSE, in responders and nonresponders across timepoints, resting‐state conditions, and scales (for MSE only), using Bayesian mixed effect modeling. As hypothesised, complexity was generally greater in the eyes‐open resting state; although, for MSE, the effect of condition differed across scale. Greater sample entropy values for scales 1–4 were elevated in the eyes‐open condition, no differences existing between Scales 5–6, and the opposite relationship (eyes closed greater than open) was observed from scales 6–10. For both the LZC and MSE global values, there was some evidence of a timepoint‐by‐condition‐by‐responder interaction. Specifically, responders displayed elevated eyes‐open LZC and MSE scales 1 and 2 values at baseline compared to nonresponders, as hypothesised. In both instances, this difference was attenuated at post‐treatment and follow‐up; a change driven by decreased and increased complexity from baseline to post‐treatment in responders and nonresponders, respectively.

An unexpected pattern was found when comparing differences between eyes‐closed and eyes‐open complexity values across timepoints and response status, particularly for LZC. In responders, the difference in LZC between conditions became progressively less pronounced from baseline to follow‐up; however, the opposite was observed for nonresponders (refer to Figure 3). An exploratory channel‐level analysis of LZC values revealed the elevated baseline eyes‐open complexity in responders was spatially localized to four left‐hemispheric frontal channels (Fp1, AF3, F3, and FC1), which displayed a treatment‐associated decrease from baseline to post‐treatment.

### 4.1 Contextualisation With Previous Literature

These results provide further evidence that baseline EEG‐derived metrics are related to oral ketamine treatment response in suicidality (Can et al. 2023). However, the contextualisation and generalisability of these findings is constrained as previous studies have examined the acute effects on complexity in response to a single intravenous ketamine infusion (Farnes et al. 2020; Murphy et al. 2023; Schartner et al. 2017). Acute (< 2 h) increases in complexity were found in all three studies, with only the most recent study by Murphy et al. (2023) reporting reduced MSE at 24‐hours. As ketamine's physiological action is intimately related to its pharmacokinetic and dynamic profile (Mion and Villevieille 2013), themselves a function of administration route, dose, formulation, and time (Yanagihara et al. 2003), further studies with similar a dose‐frequency schedule of oral ketamine and comparable EEG measurement timepoints are needed to verify these observations.

The concurrent drop in complexity and treatment response to oral ketamine is consistent with previous findings in antidepressant treatment for MDD (Méndez et al. 2012; Thomasson et al. 2000). Relatedly, the findings extend upon the broader literature on the predictive value of complexity metrics for mental health treatments (Lebiecka et al. 2018; Shalbaf et al. 2018; Torre Luque and Bornas 2017). The spatial localisation of the differences in LZC is particularly notable given elevated complexity of left‐hemispheric frontal channels was previously reported in a sample previously diagnosed with MDD (Lord and Allen 2023). Interestingly, this region has previously been implicated in treatment response to transcranial magnetic stimulation targeting the left dorsolateral prefrontal cortex (Shalbaf et al. 2018). Moreover, this localisation to the left frontal cortex is consistent with the prior (albeit mixed) literature implicating frontal alpha asymmetry in MDD (Arns et al. 2016).

In terms of mapping the observed sensor‐level dynamics to cortical regions, a previous study utilising simultaneous EEG and MRI localised the channels (Fp1, AF3, F3, and FC1) with elevated eyes‐open complexity in responders to the superior and middle frontal gyrus, and frontal pole (left Brodmann areas 6, 8, and 9) (Scrivener and Reader 2022). Considering these regions have a role in action selection, working memory, and reasoning, we speculate that oral ketamine induced recalibration of neural activity dynamics in these regions (indexed by changes in LZC) may be key to the therapeutic response.

With respect to the analysis of complexity as a function of resting‐state condition, the observed increase in complexity (LZC) during the resting‐state eyes‐open condition reaffirms recent findings (Lord and Allen 2023; Vecchio et al. 2021; Yang et al. 2023). For MSE, the observed condition‐by‐scale relationship is consistent with previous findings in a sample with MDD (Jaworska et al. 2017). This crossover (Figure 1) suggests the eyes‐open condition is more irregular at fine temporal scales (4–16 ms), whereas the eyes‐closed condition is relatively more irregular at coarser scales (> 24 ms). Together, this underscores the importance of incorporating both conditions in investigations of complexity‐related metrics, as they may capture differing aspects of network dynamics.

### 4.2 Interpretation

With respect to what the baseline elevation of complexity, and subsequent attenuation with treatment, in responders means in a physiological and psychological sense, several interpretations may be considered. The first follows from how each is calculated. In the case of LZC, complexity reflects the compressibility of the binarised time series, whereas for MSE (i.e., sample entropy) it reflects the irregularity in time‐domain amplitude patterns; defined as (dis)similarity (threshold defined by the tolerance) between a pattern of a given length (*m*) and the next sample in the sequence (*m* + 1) (see Delgado‐Bonal and Marshak 2019). In both instances, higher values could represent signal source process dynamics with greater richness or randomness (Lau et al. 2022). While this precludes a direct interpretation, some perspective can be gained by conditioning on something “meaningful” (Grassberger 2012), nay functional, such as concurrent changes in suicidality. In doing so, the observed baseline elevations of eyes open complexity in responders and the subsequent decline with treatment suggests something meaningful; it was characteristic of those who had a therapeutic response (i.e., an alleviation of suicidal thoughts).

The second follows from the perspective that signal variability is a requisite characteristic of a brain that flexibly samples state space, affording adaptability in the repertoire of perceptual, cognitive, and behavioral hypotheses that one may entertain in reasoning about the world (Garrett et al. 2013). In this sense, the elevated complexity in responders would be considered adaptive; however, this conflicts with the behavioral characteristics of the sample; highly rigid patterns of suicidal thoughts and self‐defeating behaviors.

We speculate that the heightened baseline LZC localised to the left frontal channels of responders may be indicative of a dominant attractor; a highly stable network node to which the trajectories of neuronal dynamics (on average) tend. There is reasonable evidence that some depression presentations are associated with increased within‐ and decreased between‐network connectivity (Demchenko et al. 2022), and it has been proposed that this could precipitate greater complexity (Mohammadi and Moradi 2021). Thus, the elevated left‐frontal complexity values in responders may indicate information processing is primarily occurring via the local cortical areas these channels are capturing (a preponderance of state space exploration is anchored here, which drives irregular patterns). In other words, a hyper‐refined and entrenched (canalised) brain state (Carhart‐Harris et al. 2023).

This perspective is consistent with the entropic brain and TEMP perspective, in that oral ketamine induced “noise” (entropy) is capable of therapeutically perturbing this canalised state (Carhart‐Harris et al. 2023). Albeit speculative, this leads to several testable predictions. These being that a network analysis of effective connectivity dynamics in responders would reveal a high clustering (rich‐club) coefficient between the four left‐frontal channels, and lower network integration (e.g., global efficiency) compared to nonresponders at baseline, with subsequent treatment‐induced attenuation of these features. These questions remain a matter for future research.

### 4.3 Limitations

The validity and causal interpretability of current findings are inherently limited by the nature of the study's open‐label dose‐ranging treatment design. Until this is investigated in a randomised control design with a suitable active (e.g., Midazolam) control group, randomisation to dose, medication controls, and samples of greate size, strong conclusions are not possible. Additionally, the observed changes are not only protracted (6 and 10 weeks) but also the result of multiple oral ketamine treatments. Therefore, it is impossible to know whether the decrease in complexity found in responders was indicative of a cumulative treatment effect or the typical response to even a single dose of oral ketamine.

Two key limitations of the current study was that it did not capture the acute effects of ketamine and it used a different ketamine dose and route of administration. Hence, there is limited scope for meaningful comparisons with previous literature that have examined acute effects of intravenous administrations, which display a different pharmacodynamic and kinetic profile to oral administrations (Yanagihara et al. 2003). However, the protracted measurement of oral ketamine's effects may also be considered a strength, as the transient state of “ketamine euphoria” and its associated nonspecific effects on physiology, behavior, and perception did not obscure clinical measurements.

The LZC and MSE methods are similarly not without limitations. Notably, both cases involve a discretization of the data: LZC discretizes the time series samples and MSE discretizes pattern (mis)‐matches. With respect to MSE, it is important to note the complexity values are strongly influenced by the choice of tolerance and the application of global similarity bounds, for which previous work has noted key limitations (Kosciessa et al. 2020). Despite these limitations, both metrics displayed similar sensitivity to condition and responder associated complexity changes.

Lastly, the lack of source‐localisation methods (beamforming, dipole fitting, eLORETA) applied may be considered a limitation, as the differences observed cannot be mapped to cortical sources. While we recognise this, the application of the surface Laplacian adhered to the methods of previous studies of ketamine and complexity, maximizing methodological comparability. atBy applying the (reference‐free) surface Laplacian, we were able to retain the full complement of channels. Morever, the surface Laplacian was previously shown to be the optimal montage for the determination of information theoretic complexity indices (Trujillo, Stanfield, and Vela 2017), not to mention it has the added benefit of not requiring a forward head model or individual MRI scan availability, which each introduce additional parameters, assumptions and processing.

### 4.4 Implications

These findings have several important implications for future research on signal complexity metrics, psychopathology, and ketamine treatment. Firstly, future research should not limit analyses to eyes closed only (as previously done), as it appears the eyes‐closed and open conditions capture unique signatures of complexity dynamics. Secondly, the Bayesian analysis provides the first examination of LZC and MSE posterior distributions in treatment resistant suicidality and MDD. The parameter estimates derived may inform prior specification for subsequent investigations of low‐dose ketamine and complexity metrics. With packages such as BRMS improving accessibility and usability for Bayesian statistical methods, there now exists an actionable way forward from the well‐known, but typically underplayed, limitations of stopping intentions, *p*‐values, and confidence intervals (Kruschke and Liddell 2018). Code is provided (see GitHub link) to facilitate subsequent research employing a Bayesian approach to hypothesis testing and parameter estimation.

## 5 Conclusion

This is the first examination, to our knowledge, of oral ketamine's effect on EEG‐complexity indices in suicidality and the relation to treatment response. Aside from the obvious need for further studies employing a randomised control design, robust sample sizes, and more frequent measurement timepoints, future research must examine (1) transdiagnostic relevance and (2) dynamic measures that capture network interactions. Concerning the former, if this finding represents a generality, similar spatial profiles of elevated complexity should be present in oral ketamine responders of differing mental health conditions. In terms of the later, the complexity metrics applied herein are static, revealing nothing about the (nonlinear) relationships between the dynamics at each channel. Accordingly, subsequent analyses of effective networks dynamics at rest and under “load” (i.e., event‐related paradigms) are needed to better understand oral ketamine's impact on information processing dynamics and the relationship to psychopathology. In conclusion, we provide a novel investigation of oral ketamine's effect on EEG complexity indices in suicidality within a Bayesian statistical framework.

## Author Contributions

**Jules S. Mitchell**: conceptualization, methodology, formal analysis, visualization, writing–original draft, writing–review and editing, software. **Toomas E. Anijärv**: conceptualization, methodology, software, formal analysis, writing–original draft, writing–review and editing. **Adem T. Can**: methodology, validation, investigation, resources, data curation, writing–review and editing, funding acquisition, project administration. **Megan Dutton**: project administration, investigation, data curation, validation, writing–review and editing. **Daniel F. Hermens**: validation, supervision, project administration, writing–review and editing, investigation, resources. **Jim Lagopoulos**: methodology, validation, investigation, resources, writing–review and editing, supervision, project administration, funding acquisition.

### Peer Review

The peer review history for this article is available at https://publons.com/publon/10.1002/brb3.70166.

## Supporting information



Supporting Information

## Data Availability

Research data are not shared.
