# Supplementary material for: Resting‐State Electroencephalogram Complexity Is Associated With Oral Ketamine Treatment Response: A Bayesian Analysis of Lempel–Ziv Complexity and Multiscale Entropy
Source: Brain Behav. 2024 Nov 28;14(11):e70166. doi: 10.1002/brb3.70166 (PMC11603427; doi:10.1002/brb3.70166)

**Supplementary Materials**

**Resting State Neural Complexity Differs in Responders vs. Non-Responders to Oral Ketamine Treatment: An Analysis of Lempel-Ziv Complexity and Multi-Scale Entropy**

Mitchell, Jules. S^a^*., Anijärv, Toomas. E^a,c^., Can, Adem. T^a^., Dutton, Megan^a^., Hermens, Daniel. F^a^., & Lagopoulos, Jim^b^.

1. University of Sunshine Coast, Thompson Institute, Birtinya, 4575, Sunshine Coast [Queensland], Australia
2. Thompson Brain and Mind Healthcare, Birtinya, 4575, Sunshine Coast [Queensland], Australia
3. Clinical Memory Research Unit, Department of Clinical Sciences Malmö, Faculty of Medicine, Lund University, Lund, Sweden

*Corresponding Author

Thompson Institute, 12 Innovations Parkway, Birtinya, 4575, Sunshine Coast, Queensland, Australia

jules.mitchell@research.usc.edu.au

Note: All figures are provided on Github at https://github.com/JulesMitchell/RestComplexity

**Supplementary A. Priors**

Table S1. BRMS prior specification

|  | Intercept | Beta (Mean, SD) | Sigma (Df, Mean, SD) | Standard Deviation (Mean, SD) |
| --- | --- | --- | --- | --- |
| Lempel-Ziv Complexity | Normal (0.5, 0.1) | Normal (0, 0.1) | Student_t (3, 0.05, 0.05) | Cauchy (0, 0.01) |
| Multi-Scale Entropy | Normal (1.2, 0.2) |  |  |  |

**Supplementary B. Model Comparison**

Table S2. Comparison of R-Squared values across Lempel-Ziv Complexity models (Note: Estimates calculated with chains = 2, and iterations = 2000).

| Model Formula | R^2^ | Estimated Error | Q2.5 | Q97.5 |
| --- | --- | --- | --- | --- |
| LZC \| resp_trunc(lb = 0) ~ 1 + Responder * Timepoint * Task | 0.2504 | 0.0462 | 0.1555 | 0.3386 |
| LZC \| resp_trunc(lb = 0) ~ 1 + Responder * Timepoint * Task + (1 \| Subject) | 0.7304 | 0.0249 | 0.6743 | 0.7719 |
| LZC \| resp_trunc(lb = 0) ~ 1 + Responder * Timepoint * Task + (1 + Timepoint \|\| Subject) | 0.7569 | 0.0268 | 0.6991 | 0.8033 |
| LZC \| resp_trunc(lb = 0) ~ 1 + Responder * Timepoint * Task + (1 + Timepoint + Responder \|\| Subject) | 0.7564 | 0.0272 | 0.6995 | 0.8056 |
| LZC \| resp_trunc(lb = 0) ~ 1 + Responder * Timepoint * Task + (1 + Timepoint + Responder + Task \|\| Subject) | 0.8339 | 0.0359 | 0.7508 | 0.8901 |

Table S3. Comparison of leave-one-out cross validation (LOO) expected log pointwise predictive density (ELPD) across Lempel-Ziv Complexity models. (Note: Estimates calculated with chains = 2, and iterations = 2000). Standard error, SE.

| Model Formula | ELPD_LOO (SE) |
| --- | --- |
| LZC \| resp_trunc(lb = 0) ~ 1 + Responder * Timepoint * Task + (1 + Timepoint + Responder + Task \|\| Subject) | 326.163 (9.0217) |
| LZC \| resp_trunc(lb = 0) ~ 1 + Responder * Timepoint * Task + (1 + Timepoint \|\| Subject) | 310.5318 (9.6305) |
| LZC \| resp_trunc(lb = 0) ~ 1 + Responder * Timepoint * Task + (1 + Timepoint + Responder \|\| Subject) | 309.8358 (9.5893) |
| LZC \| resp_trunc(lb = 0) ~ 1 + Responder * Timepoint * Task + (1 \| Subject) | 308.3047 (9.8947) |
| LZC \| resp_trunc(lb = 0) ~ 1 + Responder * Timepoint * Task | 232.1536 (10.3459) |

Table S4. Comparison of R-Squared values across Multi-Scale Entropy models (Note: Estimates calculated with chains = 2, and iterations = 2000).

| Model Formula | Response Variable | R^2^ | Est.Error | Q2.5 | Q97.5 |
| --- | --- | --- | --- | --- | --- |
| mvbind (Scale 1, Scale 2, … Scale 10) \| resp_trunc(lb = 0) ~ 1 + Responder * Timepoint * Task | Scale 1 | 0.2466 | 0.0466 | 0.154 | 0.3344 |
|  | Scale 2 | 0.2375 | 0.045 | 0.1499 | 0.324 |
|  | Scale 3 | 0.2035 | 0.0448 | 0.1156 | 0.2892 |
|  | Scale 4 | 0.1402 | 0.04 | 0.0665 | 0.2212 |
|  | Scale 5 | 0.1161 | 0.0363 | 0.0505 | 0.1917 |
|  | Scale 6 | 0.1617 | 0.0422 | 0.0854 | 0.2478 |
|  | Scale 7 | 0.2217 | 0.0434 | 0.1326 | 0.3059 |
|  | Scale 8 | 0.2506 | 0.0468 | 0.1586 | 0.3433 |
|  | Scale 9 | 0.2604 | 0.0457 | 0.1664 | 0.3449 |
|  | Scale 10 | 0.2376 | 0.046 | 0.1458 | 0.3248 |
| mvbind (Scale 1, Scale 2, … Scale 10) \| resp_trunc(lb = 0) ~ 1 + Responder * Timepoint * Task + (1 \|p\| Subject) | Scale 1 | 0.7581 | 0.019 | 0.7174 | 0.7904 |
|  | Scale 2 | 0.781 | 0.0167 | 0.7424 | 0.8094 |
|  | Scale 3 | 0.7716 | 0.0172 | 0.7349 | 0.7997 |
|  | Scale 4 | 0.7444 | 0.0198 | 0.6998 | 0.7775 |
|  | Scale 5 | 0.7285 | 0.0209 | 0.6831 | 0.7639 |
|  | Scale 6 | 0.7504 | 0.019 | 0.7083 | 0.7813 |
|  | Scale 7 | 0.7788 | 0.0168 | 0.7415 | 0.8063 |
|  | Scale 8 | 0.7853 | 0.0159 | 0.749 | 0.812 |
|  | Scale 9 | 0.789 | 0.0153 | 0.7553 | 0.8146 |
|  | Scale 10 | 0.7764 | 0.0173 | 0.7393 | 0.8064 |
| mvbind (Scale 1, Scale 2, … Scale 10) \| resp_trunc(lb = 0) ~ 1 + Responder * Timepoint * Task + (1 + Timepoint \|p\| Subject) | Scale 1 | 0.7531 | 0.0212 | 0.7072 | 0.7885 |
|  | Scale 2 | 0.7752 | 0.0173 | 0.7369 | 0.8046 |
|  | Scale 3 | 0.7644 | 0.0187 | 0.7225 | 0.7953 |
|  | Scale 4 | 0.7384 | 0.0211 | 0.6919 | 0.7743 |
|  | Scale 5 | 0.721 | 0.0231 | 0.6676 | 0.7586 |
|  | Scale 6 | 0.7422 | 0.0215 | 0.6954 | 0.7768 |
|  | Scale 7 | 0.7725 | 0.0183 | 0.7306 | 0.8013 |
|  | Scale 8 | 0.7794 | 0.0178 | 0.7391 | 0.8081 |
|  | Scale 9 | 0.7834 | 0.017 | 0.7457 | 0.8116 |
|  | Scale 10 | 0.7743 | 0.0181 | 0.7348 | 0.8053 |
| mvbind (Scale 1, Scale 2, … Scale 10) \| resp_trunc(lb = 0) ~ 1 + Responder * Timepoint * Task + (1 + Timepoint + Responder\|p\| Subject) | Scale 1 | 0.7715 | 0.0236 | 0.7215 | 0.8121 |
|  | Scale 2 | 0.7932 | 0.0217 | 0.7461 | 0.831 |
|  | Scale 3 | 0.8051 | 0.0206 | 0.7609 | 0.8385 |
|  | Scale 4 | 0.8 | 0.0204 | 0.7551 | 0.8355 |
|  | Scale 5 | 0.7925 | 0.0263 | 0.7331 | 0.8366 |
|  | Scale 6 | 0.8259 | 0.0214 | 0.7757 | 0.86 |
|  | Scale 7 | 0.8451 | 0.0193 | 0.8011 | 0.8758 |
|  | Scale 8 | 0.8483 | 0.0193 | 0.8039 | 0.8794 |
|  | Scale 9 | 0.8411 | 0.0199 | 0.7965 | 0.873 |
|  | Scale 10 | 0.8213 | 0.0209 | 0.7749 | 0.858 |
| mvbind (Scale 1, Scale 2, … Scale 10) \| resp_trunc(lb = 0) ~ 1 + Responder * Timepoint * Task + (1 + Timepoint + Responder + Task \|p\| Subject) | Scale 1 | 0.8629 | 0.0218 | 0.8135 | 0.8989 |
|  | Scale 2 | 0.8976 | 0.0159 | 0.8605 | 0.9229 |
|  | Scale 3 | 0.9256 | 0.0119 | 0.8978 | 0.9443 |
|  | Scale 4 | 0.9341 | 0.0103 | 0.9117 | 0.951 |
|  | Scale 5 | 0.9241 | 0.0113 | 0.8976 | 0.9433 |
|  | Scale 6 | 0.9209 | 0.0113 | 0.8953 | 0.9396 |
|  | Scale 7 | 0.9227 | 0.0106 | 0.9002 | 0.9407 |
|  | Scale 8 | 0.9246 | 0.0108 | 0.9006 | 0.942 |
|  | Scale 9 | 0.9206 | 0.0112 | 0.8951 | 0.9391 |
|  | Scale 10 | 0.9042 | 0.0145 | 0.8728 | 0.9283 |

Table S5. Comparison of leave-one-out cross validation (LOO) expected log pointwise predictive density (ELPD) across Multi-Scale Entropy models. (Note: Estimates calculated with chains = 2, and iterations = 2000). Standard error, SE.

| Model Formula | ELPD LOO (SE) |
| --- | --- |
| mvbind (Scale 1, Scale 2, … Scale 10) \| resp_trunc(lb = 0) ~ 1 + Responder * Timepoint * Task + (1 + Timepoint + Responder + Task \|p\| Subject) | 3313.056 (51.297) |
| mvbind (Scale 1, Scale 2, … Scale 10) \| resp_trunc(lb = 0) ~ 1 + Responder * Timepoint * Task + (1 + Timepoint + Responder\|p\| Subject) | 2831.846 (78.339) |
| mvbind (Scale 1, Scale 2, … Scale 10) \| resp_trunc(lb = 0) ~ 1 + Responder * Timepoint * Task + (1 \|p\| Subject) | 2757.517 (94.615) |
| mvbind (Scale 1, Scale 2, … Scale 10) \| resp_trunc(lb = 0) ~ 1 + Responder * Timepoint * Task + (1 + Timepoint \|p\| Subject) | 2731.215 (93.834) |
| mvbind (Scale 1, Scale 2, … Scale 10) \| resp_trunc(lb = 0) ~ 1 + Responder * Timepoint * Task | 1820.107 (77.324) |

Table S6. R-Squared values for channel-level Lempel-Ziv Complexity model.

|  | R^2^ | Est.Error | Q2.5 | Q97.5 |
| --- | --- | --- | --- | --- |
| Fp1\| resp_trunc(lb = 0) ~ 1 + Responder * Timepoint * Task + (1 + Timepoint + Responder + Task \|\| Subject) | 0.773 | 0.034 | 0.696 | 0.831 |
| AF3 \| resp_trunc(lb = 0) ~ 1 + Responder * Timepoint * Task + (1 + Timepoint + Responder + Task \|\| Subject) | 0.783 | 0.031 | 0.713 | 0.838 |
| FC1 \| resp_trunc(lb = 0) ~ 1 + Responder * Timepoint * Task + (1 + Timepoint + Responder + Task \|\| Subject) | 0.793 | 0.039 | 0.703 | 0.855 |
| F3\| resp_trunc(lb = 0) ~ 1 + Responder * Timepoint * Task + (1 + Timepoint + Responder + Task \|\| Subject) | 0.671 | 0.046 | 0.571 | 0.748 |

Table S7. Leave-one-out cross validation (LOO) expected log pointwise predictive density (ELPD) for channel-level Lempel-Ziv Complexity model. Standard error, SE.

|  | ELPD_LOO (SE) |
| --- | --- |
| mvbind (Fp1, AF3, FC1, F3)\| resp_trunc(lb = 0) ~ 1 + Responder * Timepoint * Task + (1 + Timepoint + Responder + Task \|\| Subject) | 937.987 (29.913) |

**Supplementary C. Prior predictive checks (Final Model Only)**

Figure S1. Histogram plots of prior predicted Lempel-Ziv Complexity values


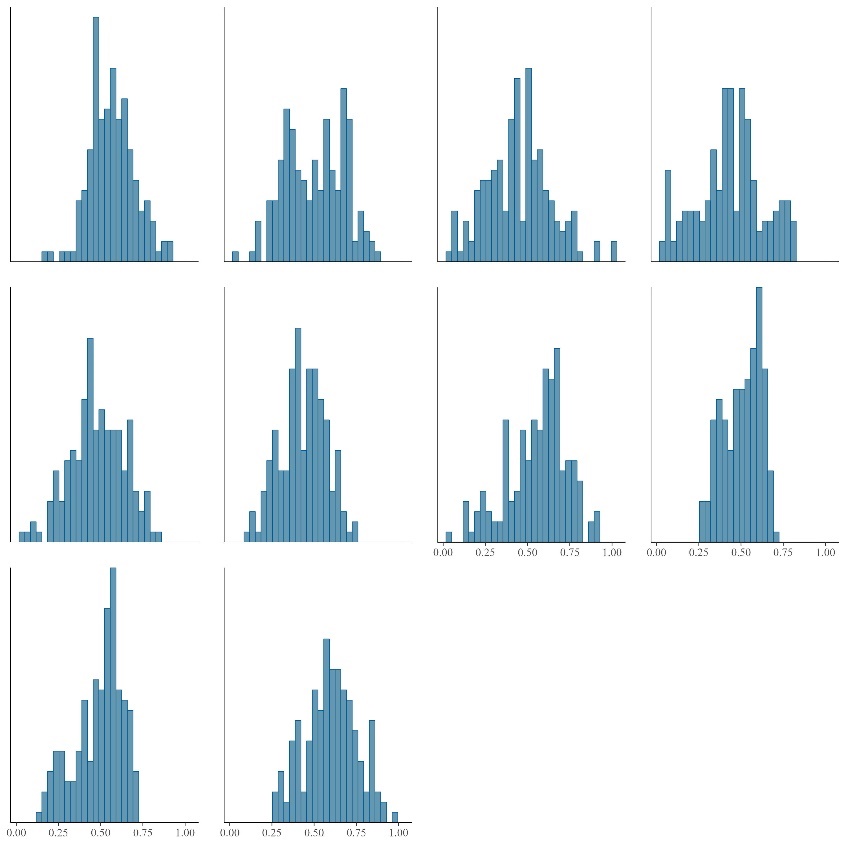


Figure S2. Histogram plots of prior predicted Multi-Scale Entropy values for Scale 1. Note: Plots for MSE scales 2-10 are available at GitHub link.


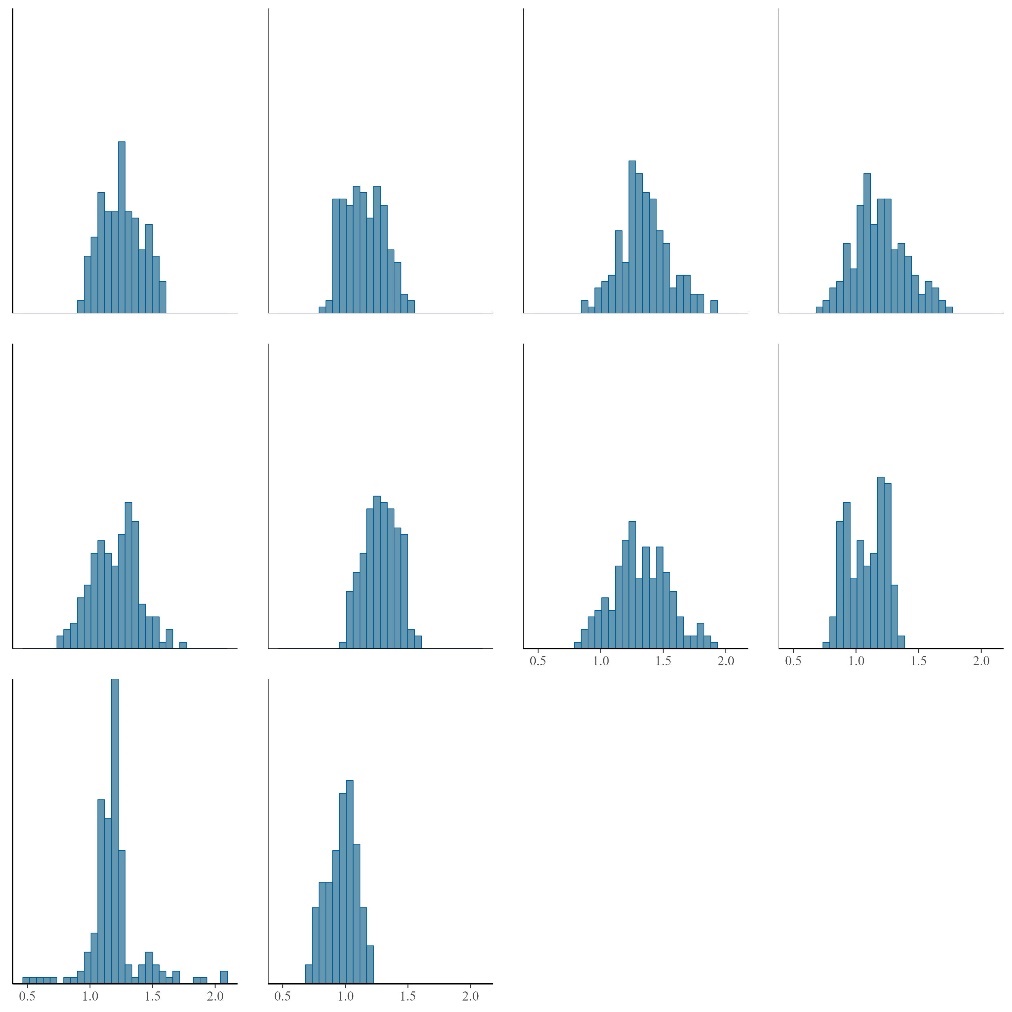


**Supplementary D. Model Performance and Convergence**

**Autocorrelation**


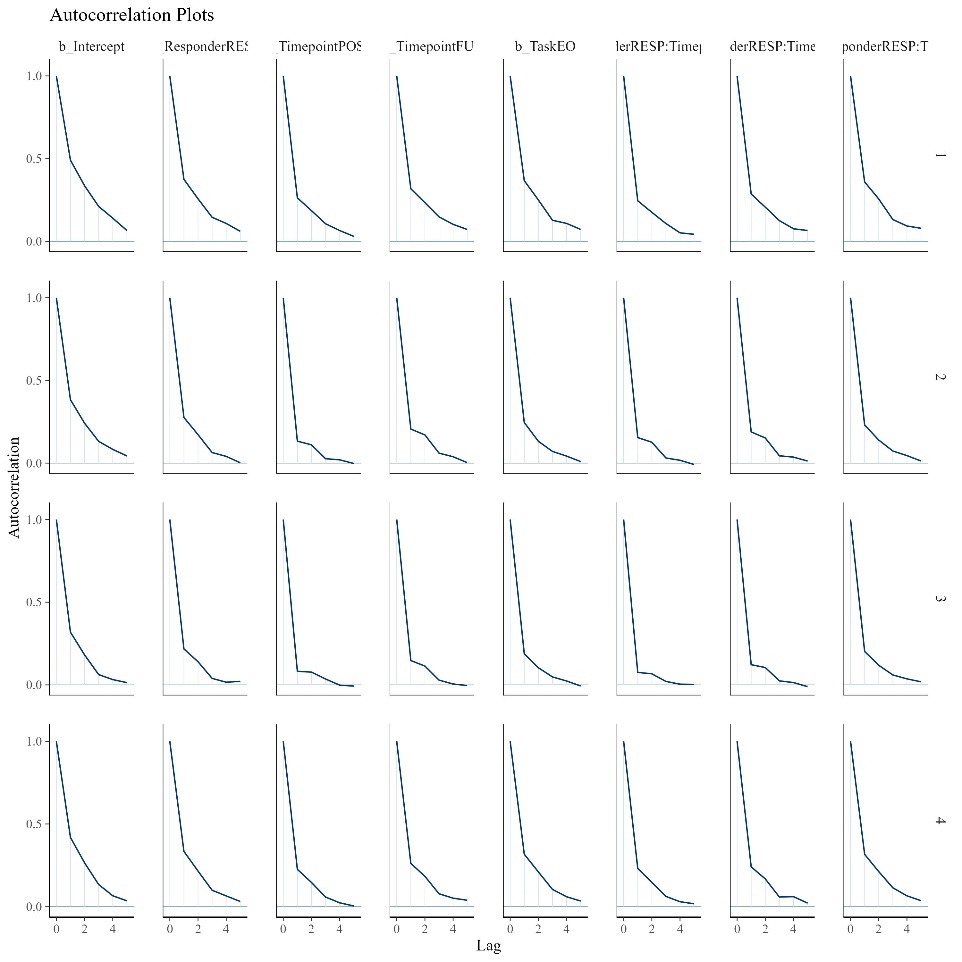
Figure S3a. Autocorrelation plots for Lempel-Ziv Complexity. Note: From left to right, the columns represent;

Figure S3b. Autocorrelation plots for Lempel-Ziv Complexity. Note: From left to right, the columns represent;


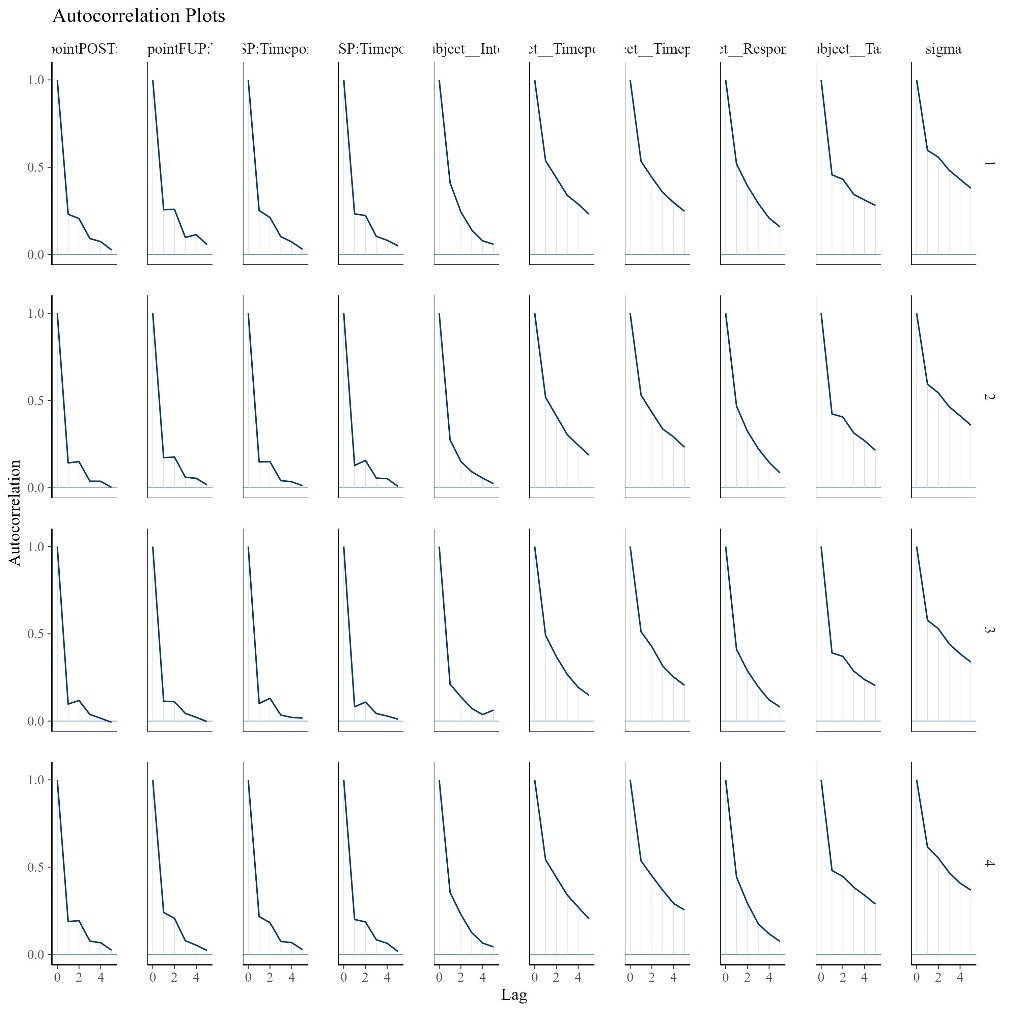


Figure S4a. Autocorrelation plots for Multi-Scale Entropy Intercept. Note: From left to right, the columns represent scales 1-10. Note: Plots for MSE scales 2-10 are available at GitHub link.


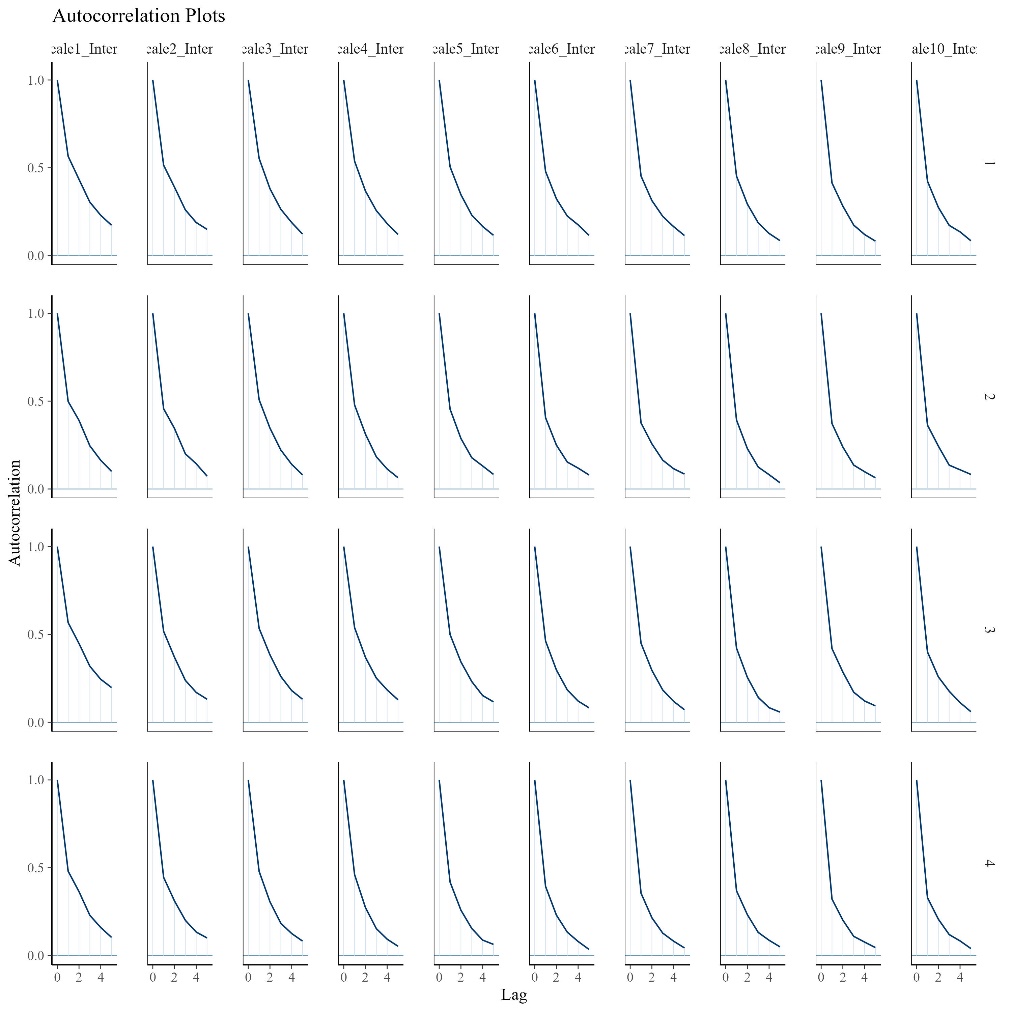


Figure S4b. Autocorrelation plots for Multi-Scale Entropy Scale 1. Note: From left to right, the columns represent scale 1 betas for fixed effects and interactions. Note: Plots for MSE scales 2-10 are available at GitHub link.


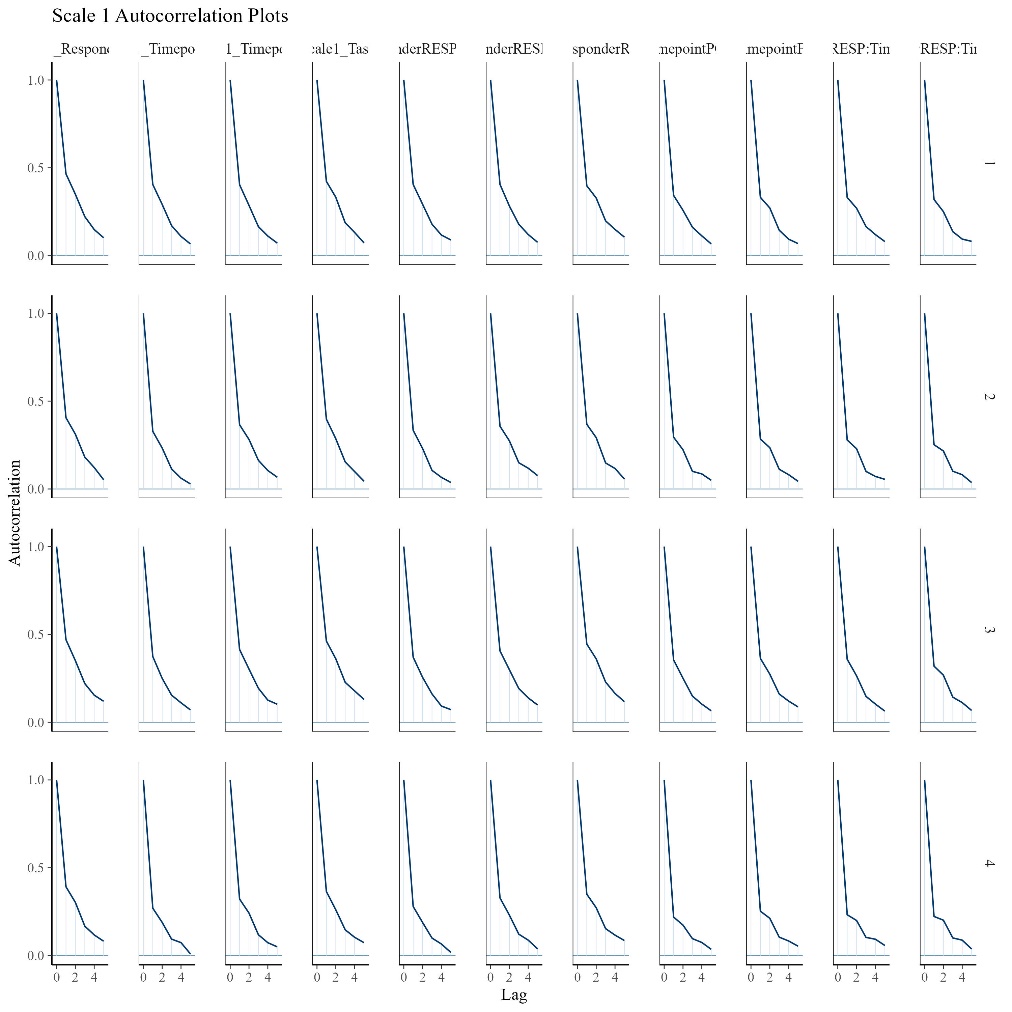


**R-hat Values**

Figure S5. Histogram plot of Lempel-Ziv Complexity model R-Hat values.


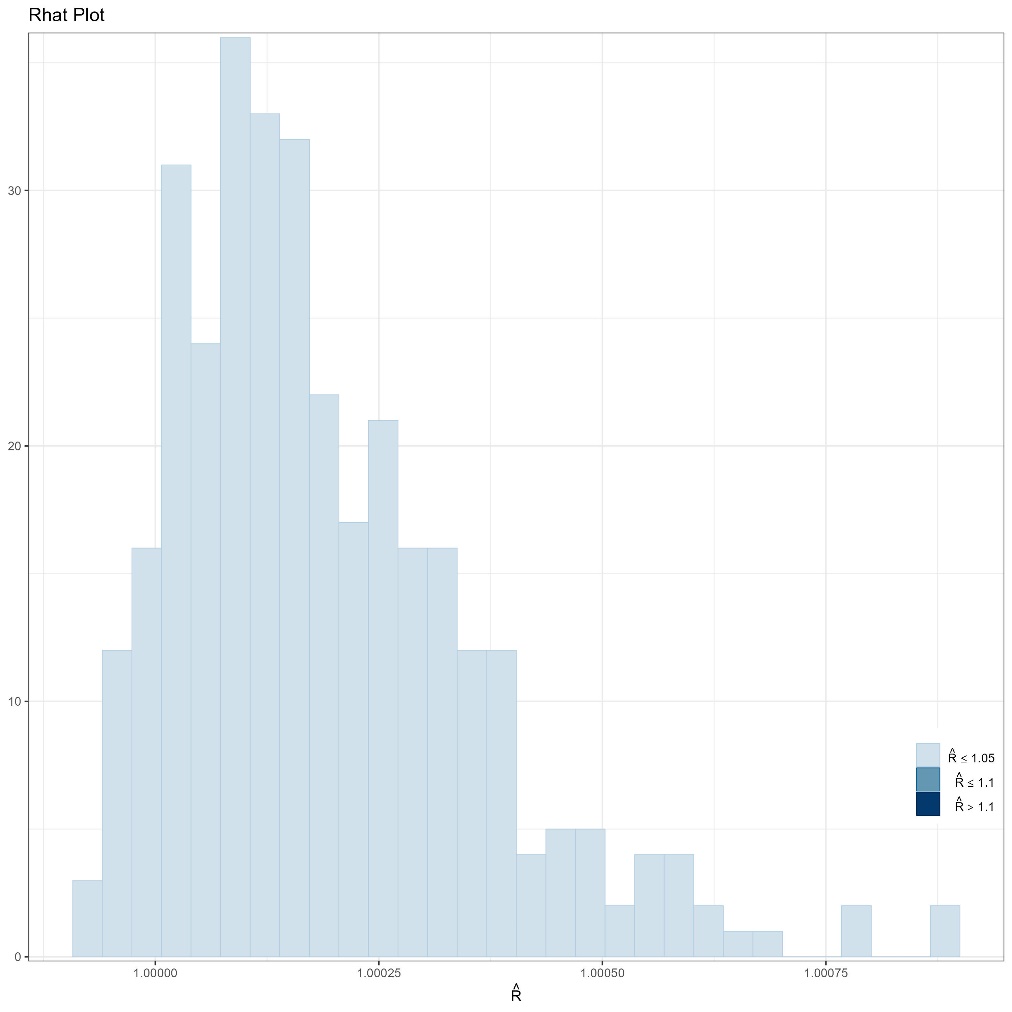


Figure S6. Histogram plot of Multi-Scale Entropy model R-Hat values.


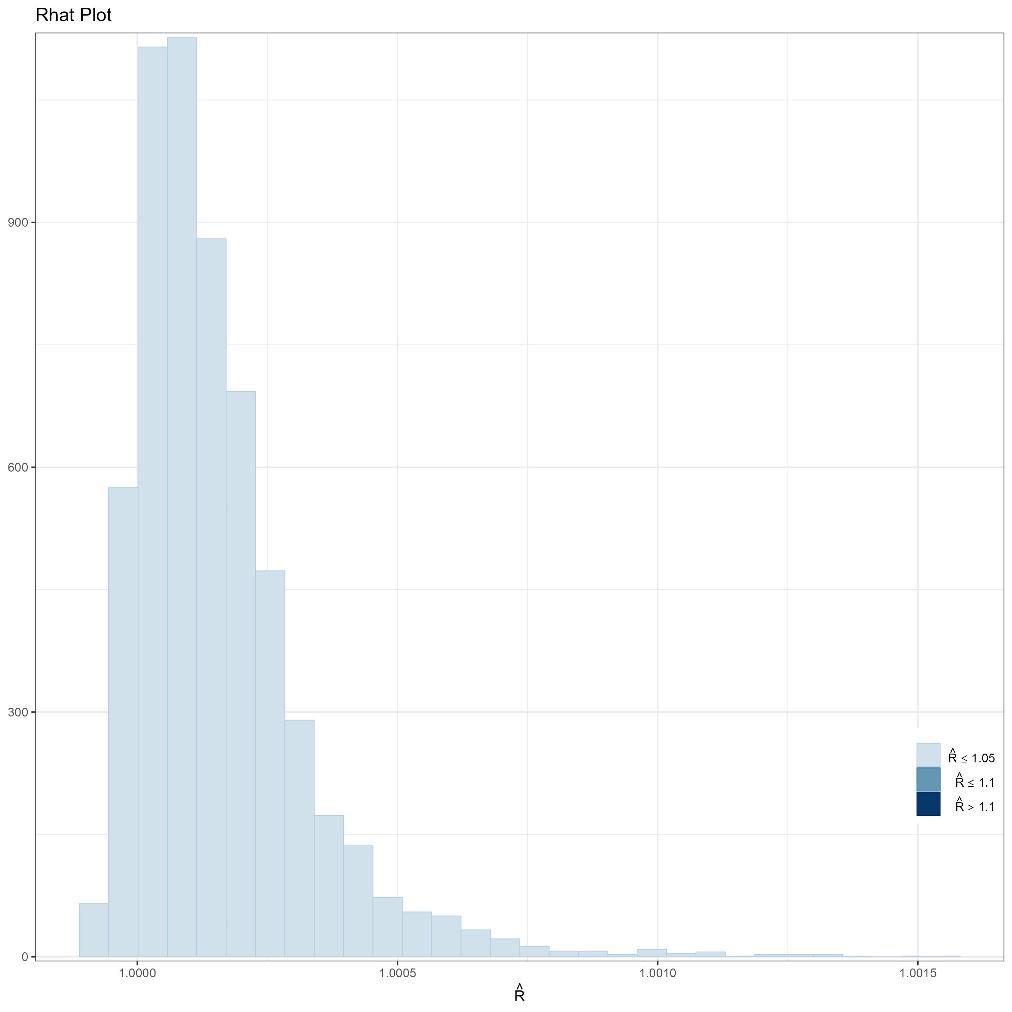


**Error Distribution**

Figure S7. Histogram plot of Lempel-Ziv Complexity model error distribution.


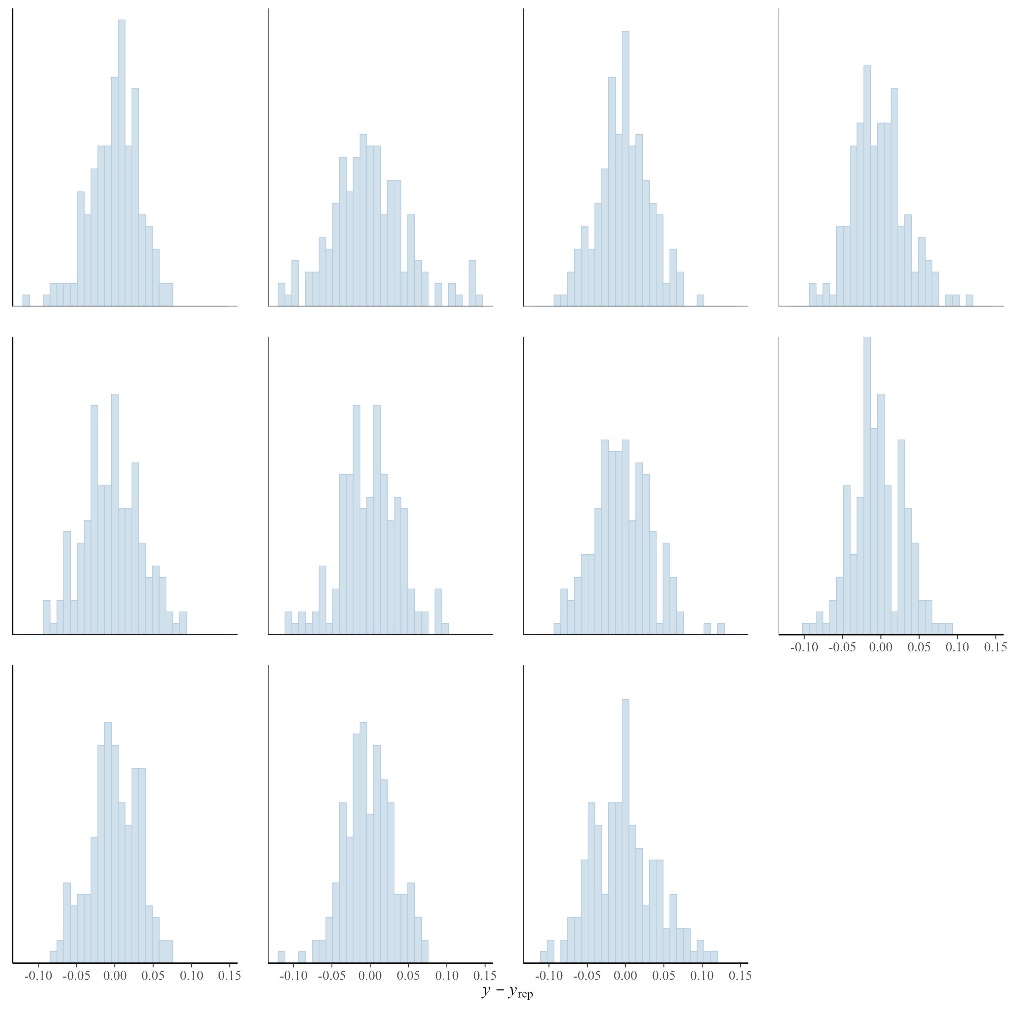


Figure S8. Histogram plot of Multi-Scale Entropy model error distribution for Scale 1. Note: Plots for MSE scales 2-10 available at GitHub link.


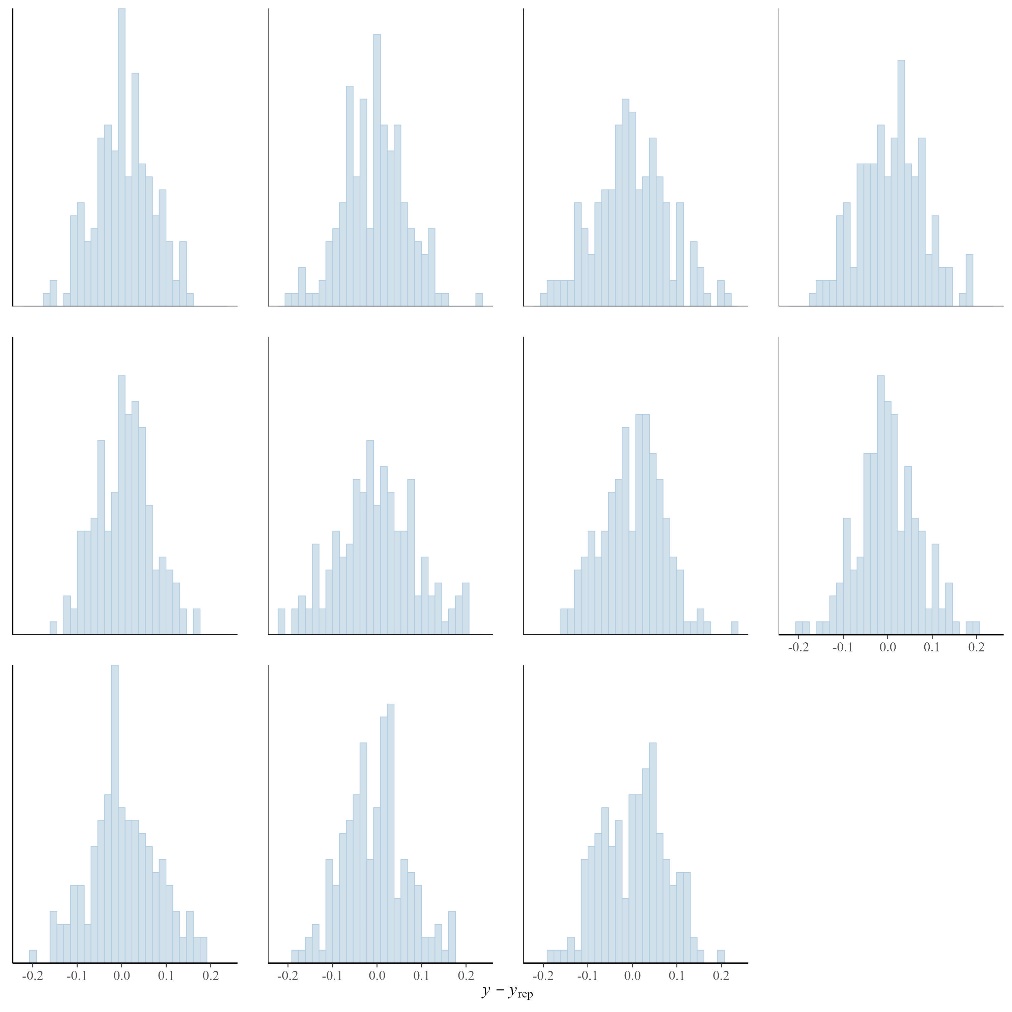


**Effective Sampling**

Figure S9. Histogram plot of Lempel-Ziv Complexity model effective sampling distribution.


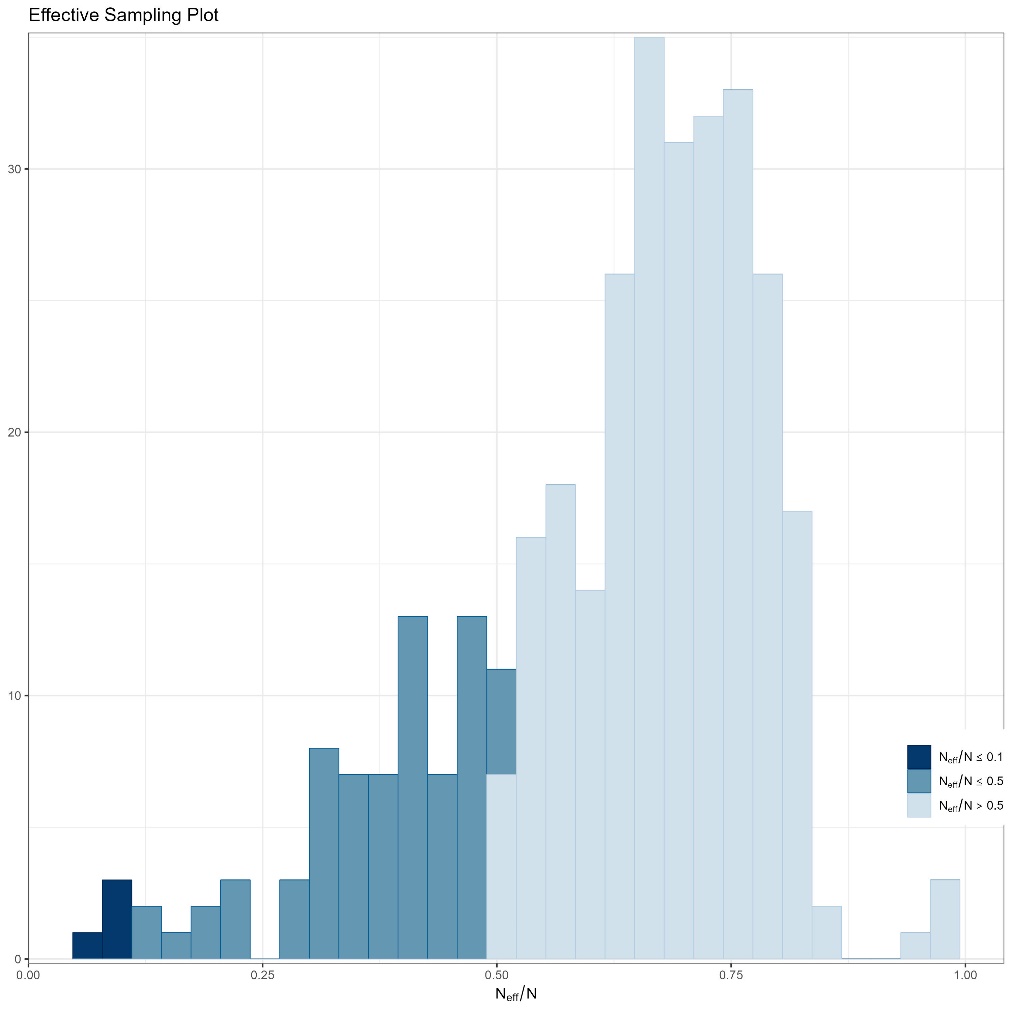


Figure S10. Histogram plot of Multi-Scale Entropy model effective sampling distribution.


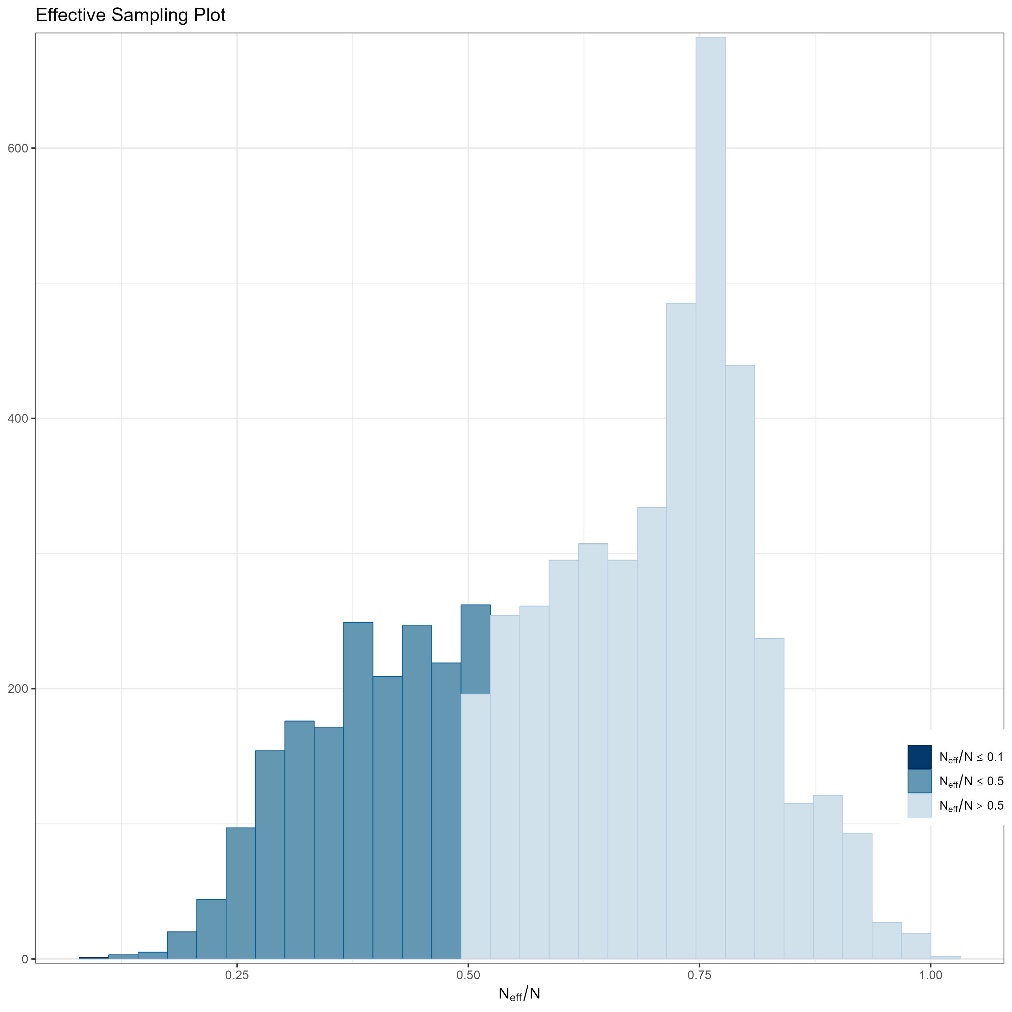


**Observed versus Predicted**

Figure S11. Scatter plot of observed versus predicted values for the Lempel-Ziv Complexity model.


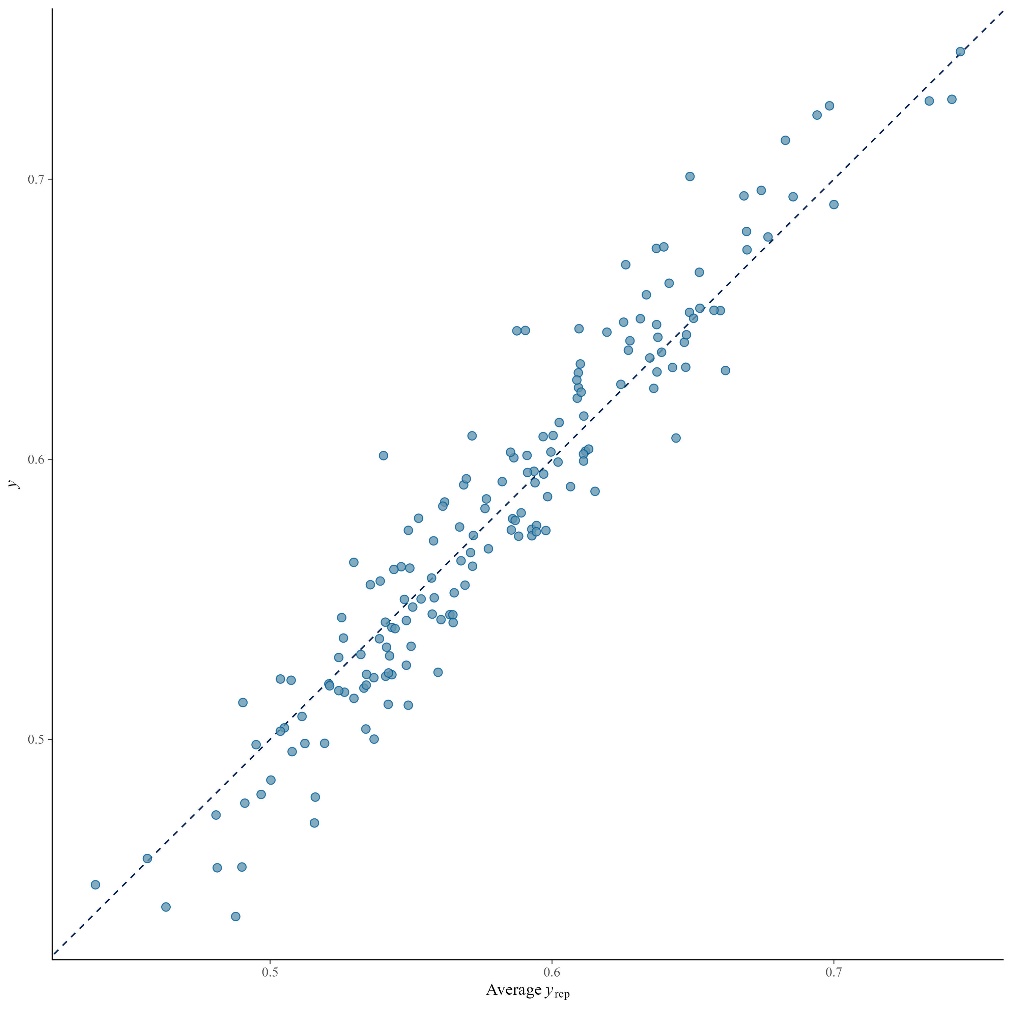


Figure S12.Scatter plot of observed versus predicted values for the Multi-Scale Entropy model for Scale 1. Note: Plots for MSE scales 2-10 available at GitHub link.


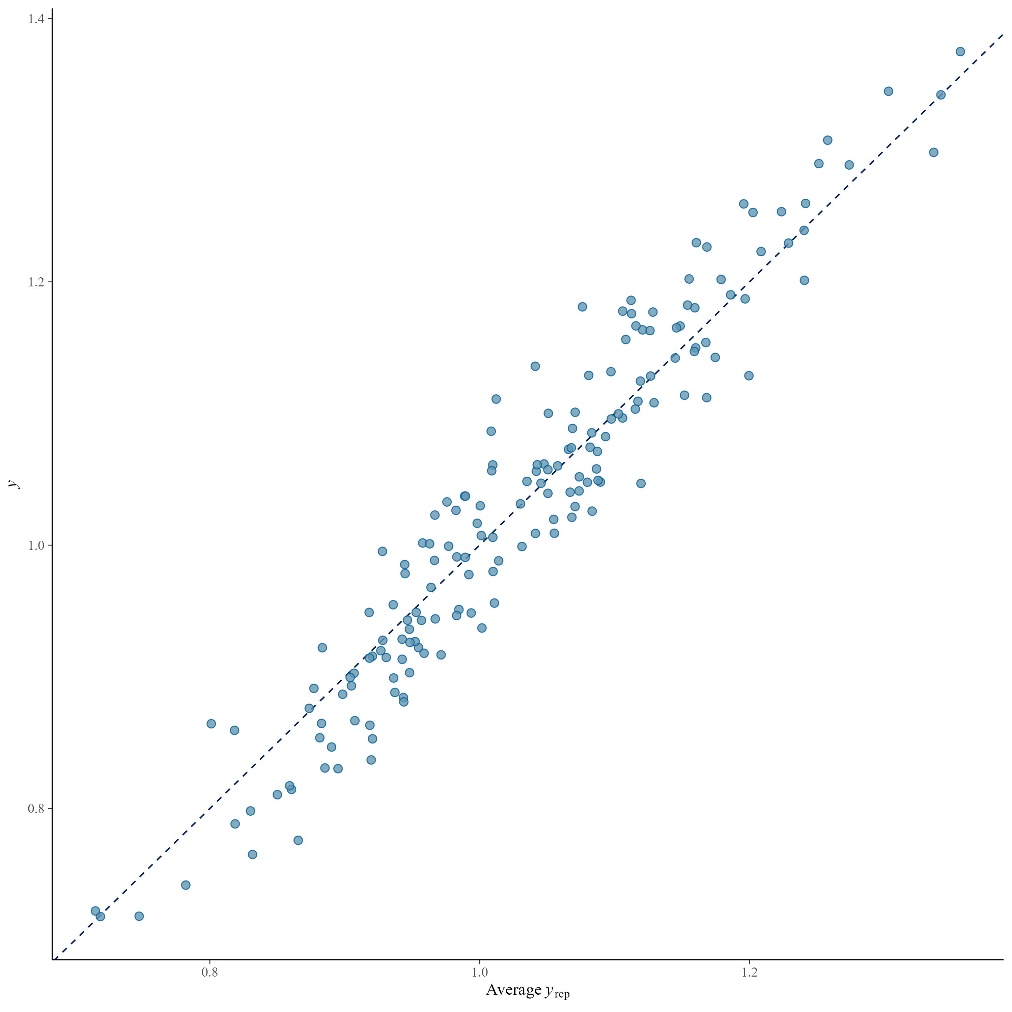


**Trace Plots**

Figure S13a. Trace plots of model chains for the Lempel-Ziv Complexity model.


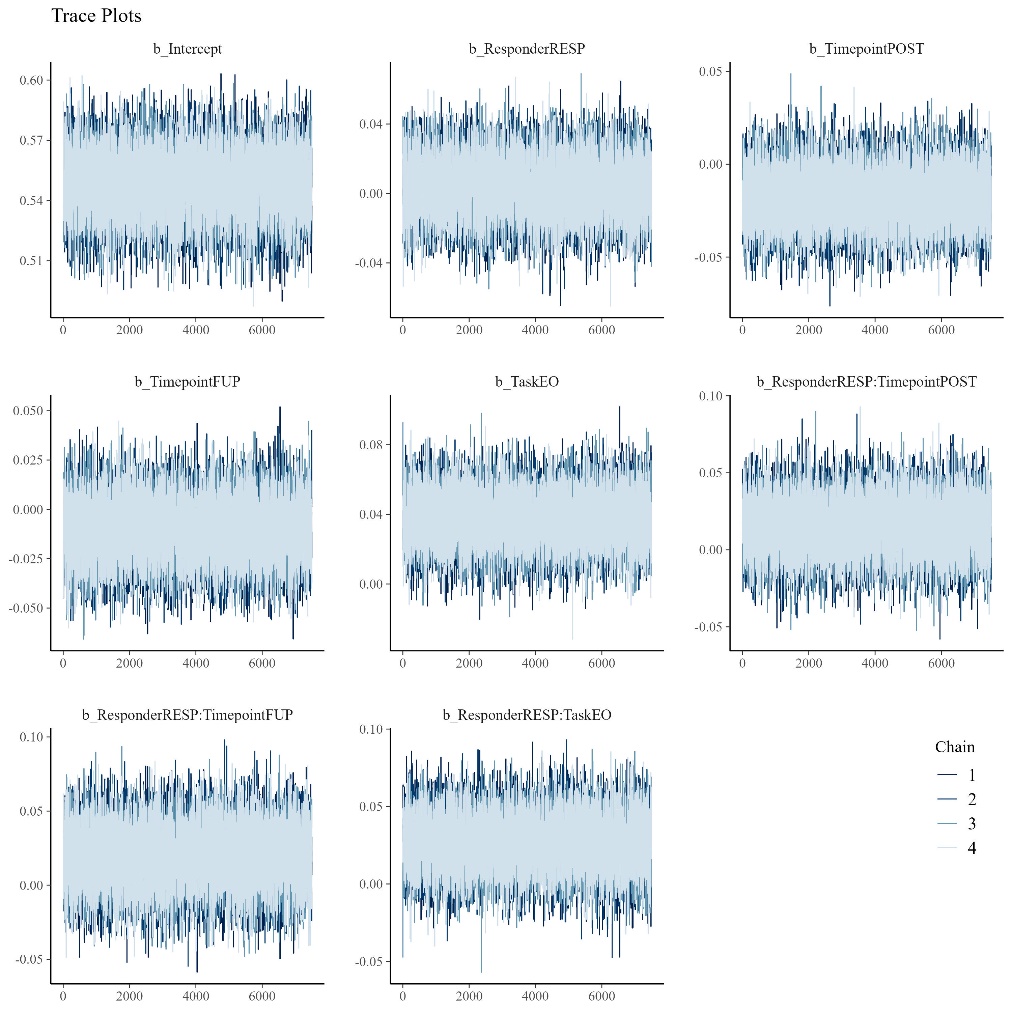


Figure 13b. Trace plots of model chains for the Lempel-Ziv Complexity model.


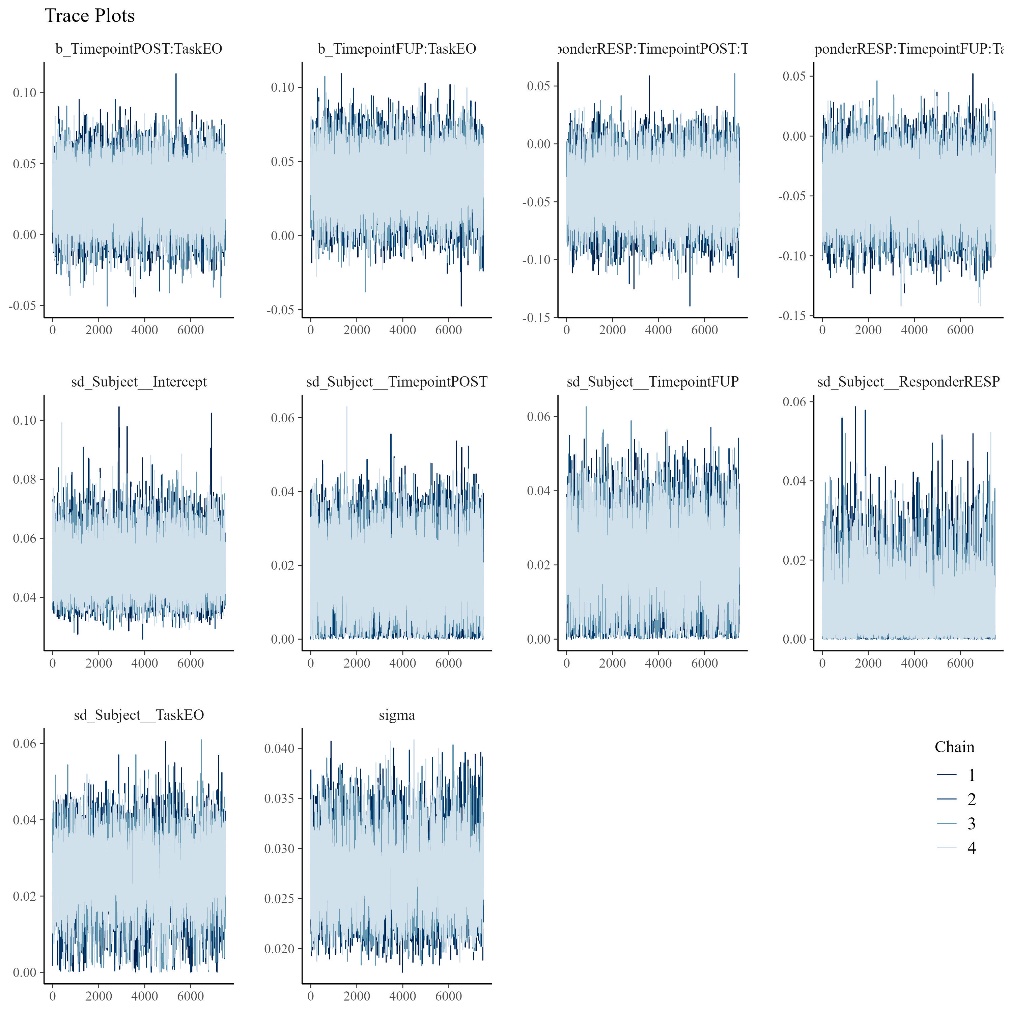


Figure S14a. Trace plots of model chains for the Multi-Scale Entropy model intercept estimates. Note: Plots for MSE scales 2-10 available at GitHub link.


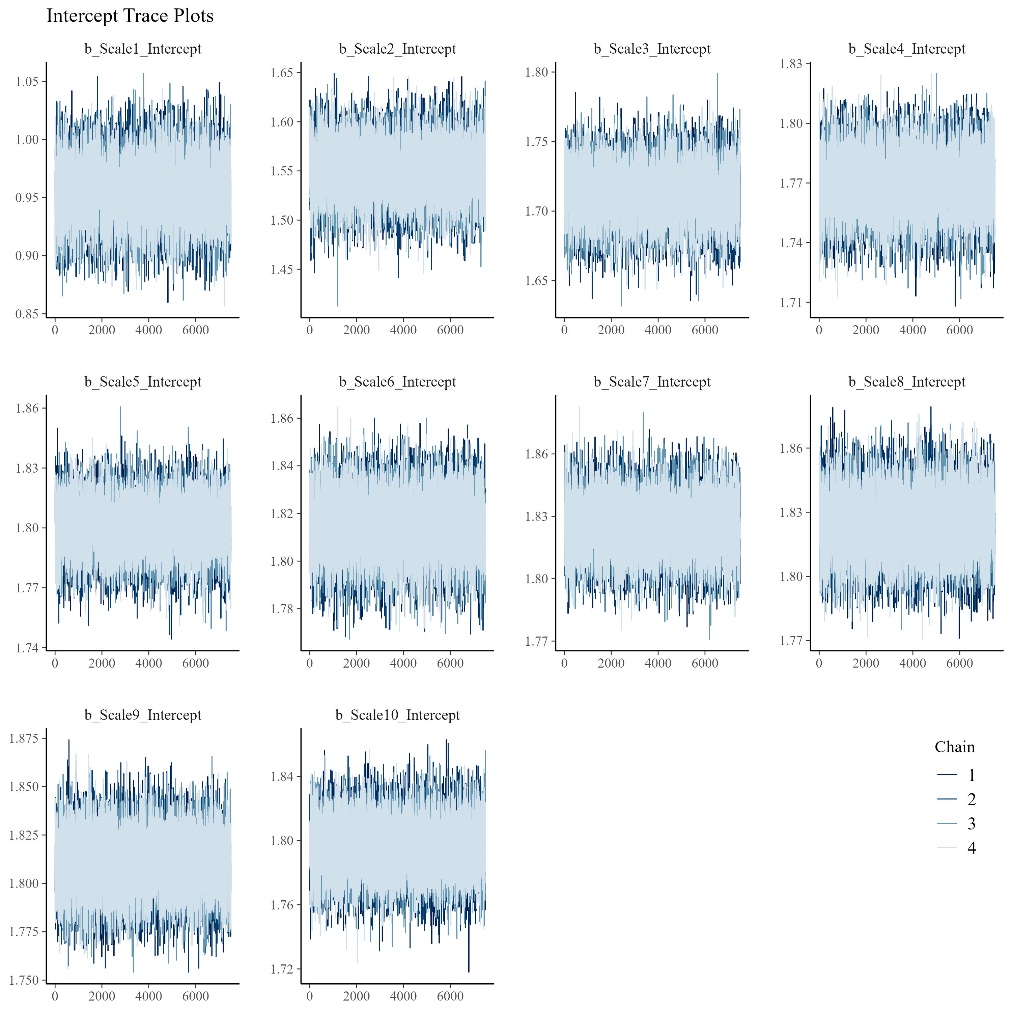


Figure S14b. Trace plots of model chains for the Multi-Scale Entropy model scale 1 estimates. Note: Plots for MSE scales 2-10 available at GitHub link.


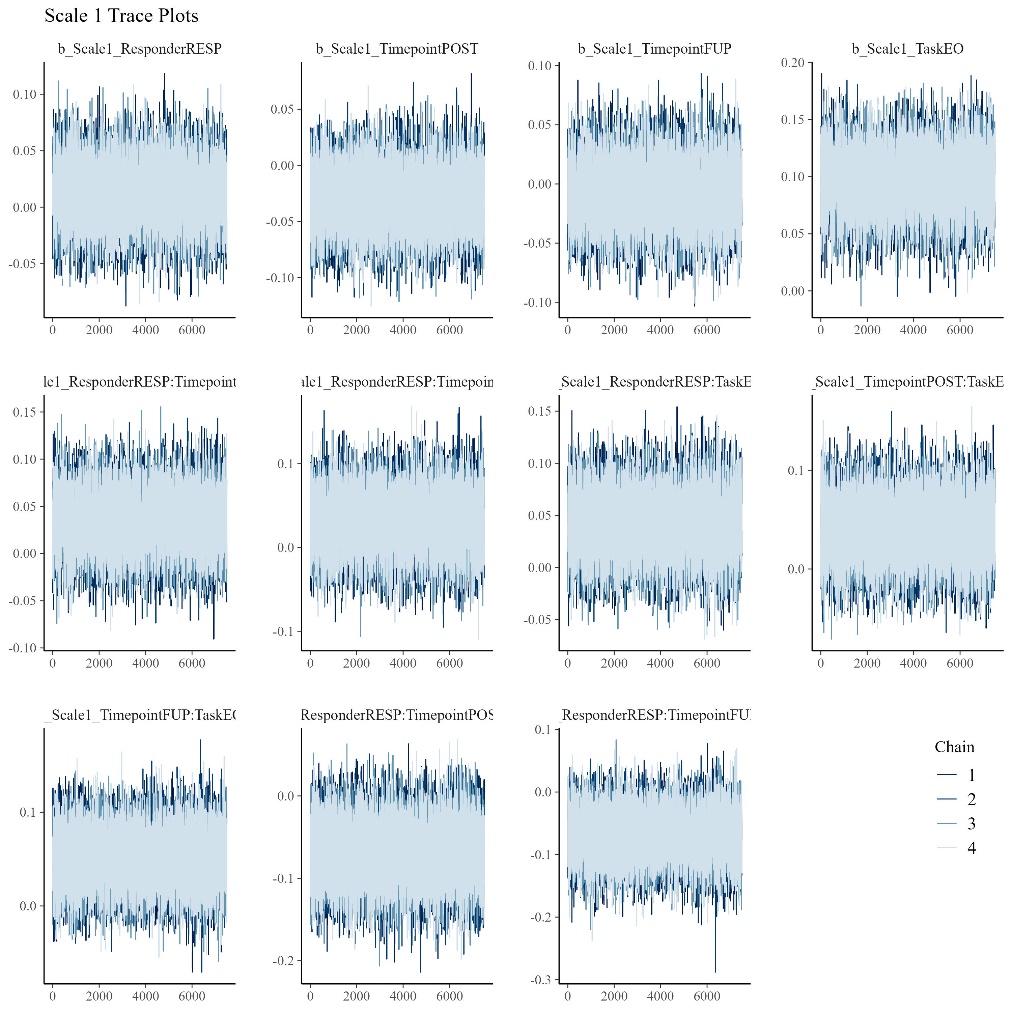


**Supplementary E. Posterior Predictive Checks**

**Overall**

Figure S15. Density overlay plot comparing observed versus simulated Lempel-Ziv Complexity values from the posterior predictive distribution.


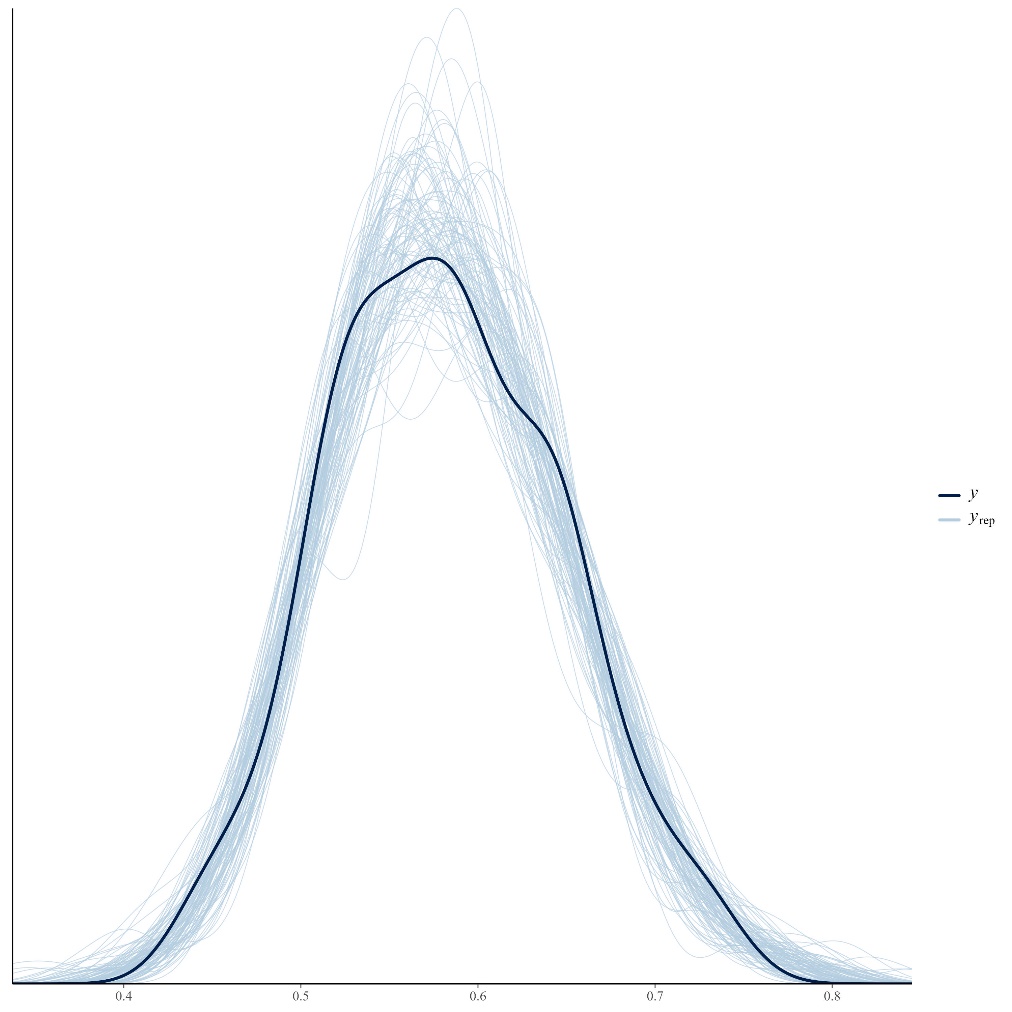


Figure S16. Density overlay plot comparing observed versus simulated Multi-Scale Entropy Scale 1 values from the posterior predictive distribution. Note: Plots for MSE scales 2-10 available at GitHub link.


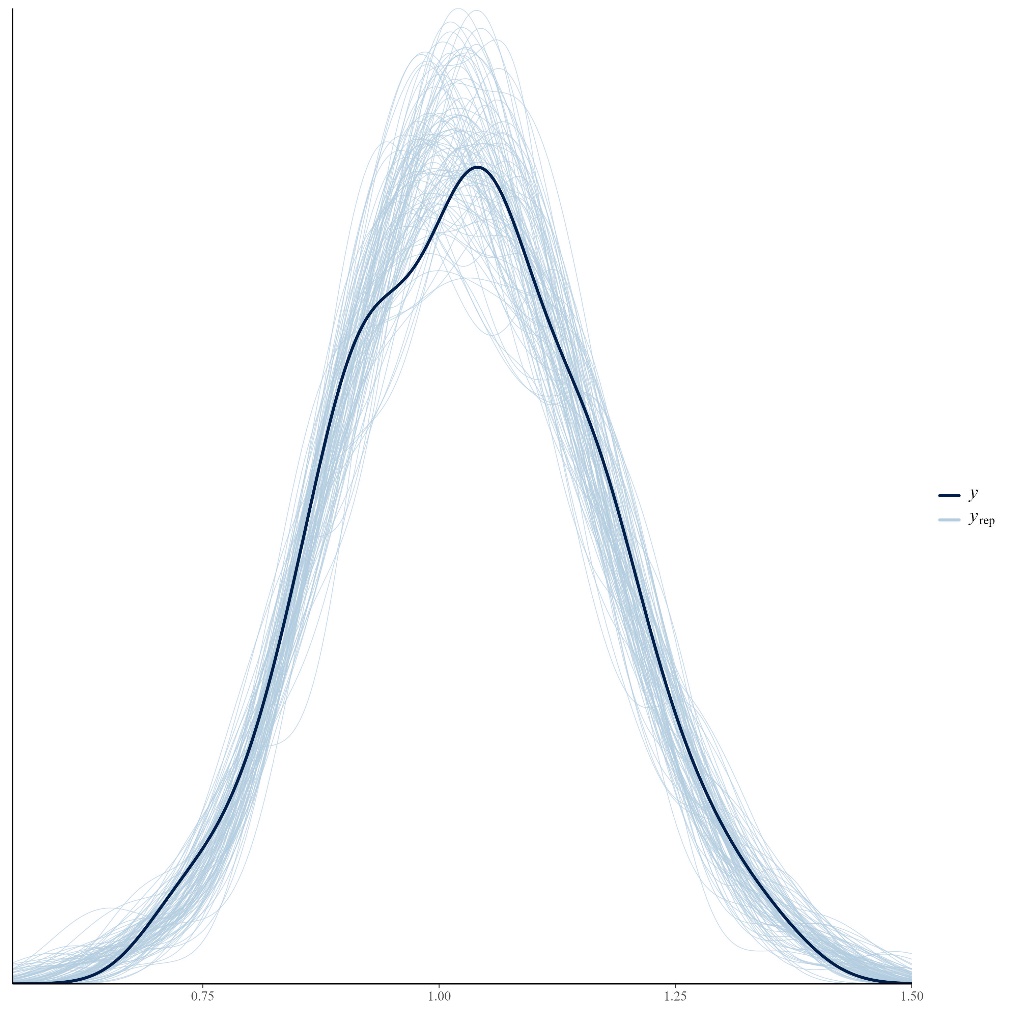


**Minimum Values**

Figure S17a. Histogram plot comparing observed versus simulated Lempel-Ziv Complexity minimum values across responders from the posterior predictive distribution.


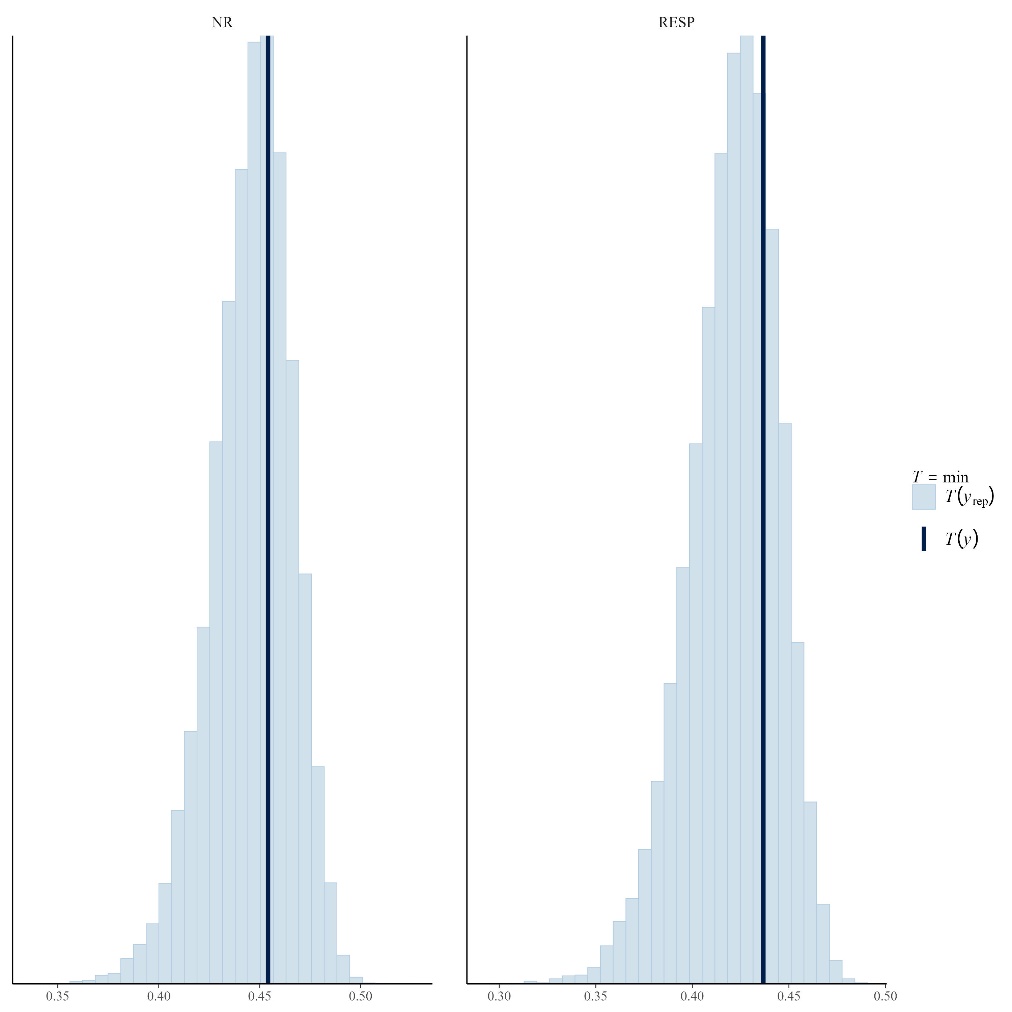


Figure S17b. Histogram plot comparing observed versus simulated Lempel-Ziv Complexity minimum values across tasks from the posterior predictive distribution.


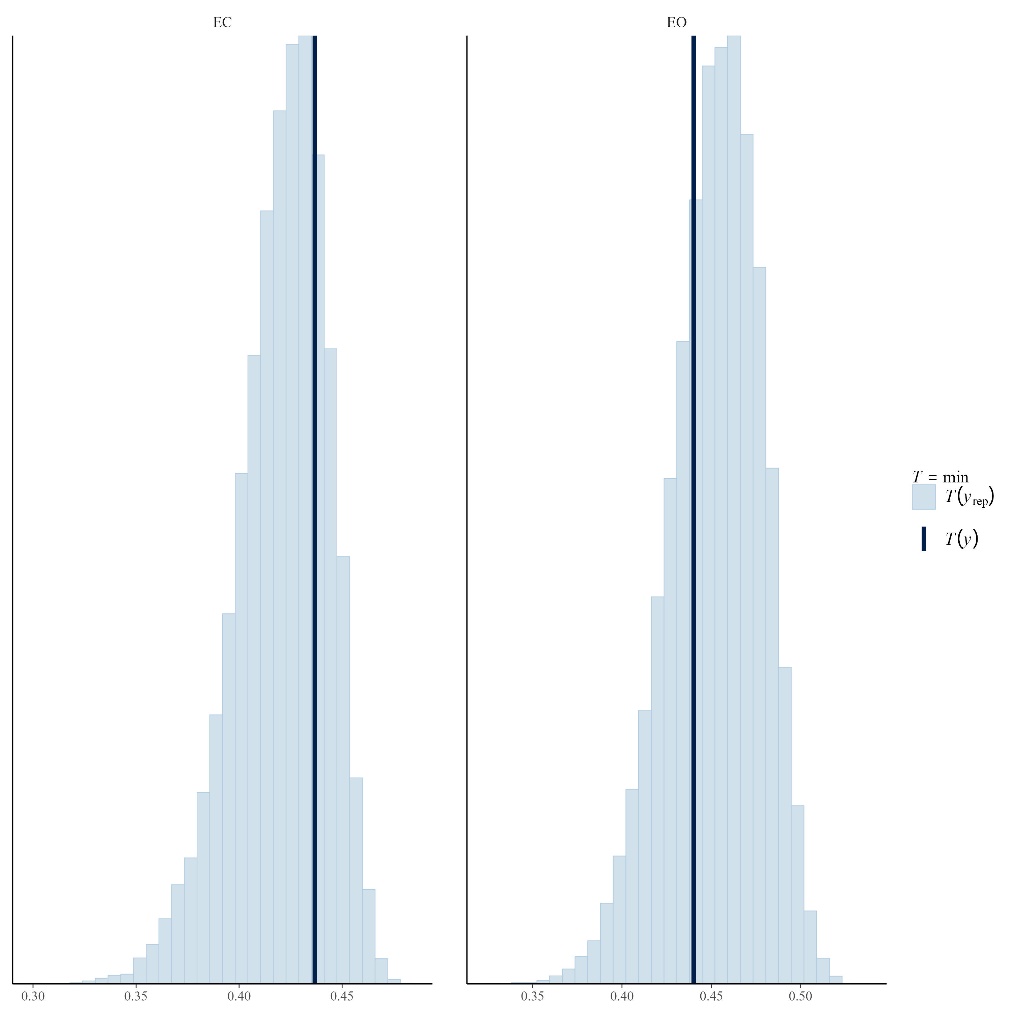


Figure S17c. Histogram plot comparing observed versus simulated Lempel-Ziv Complexity minimum values across timepoints from the posterior predictive distribution.


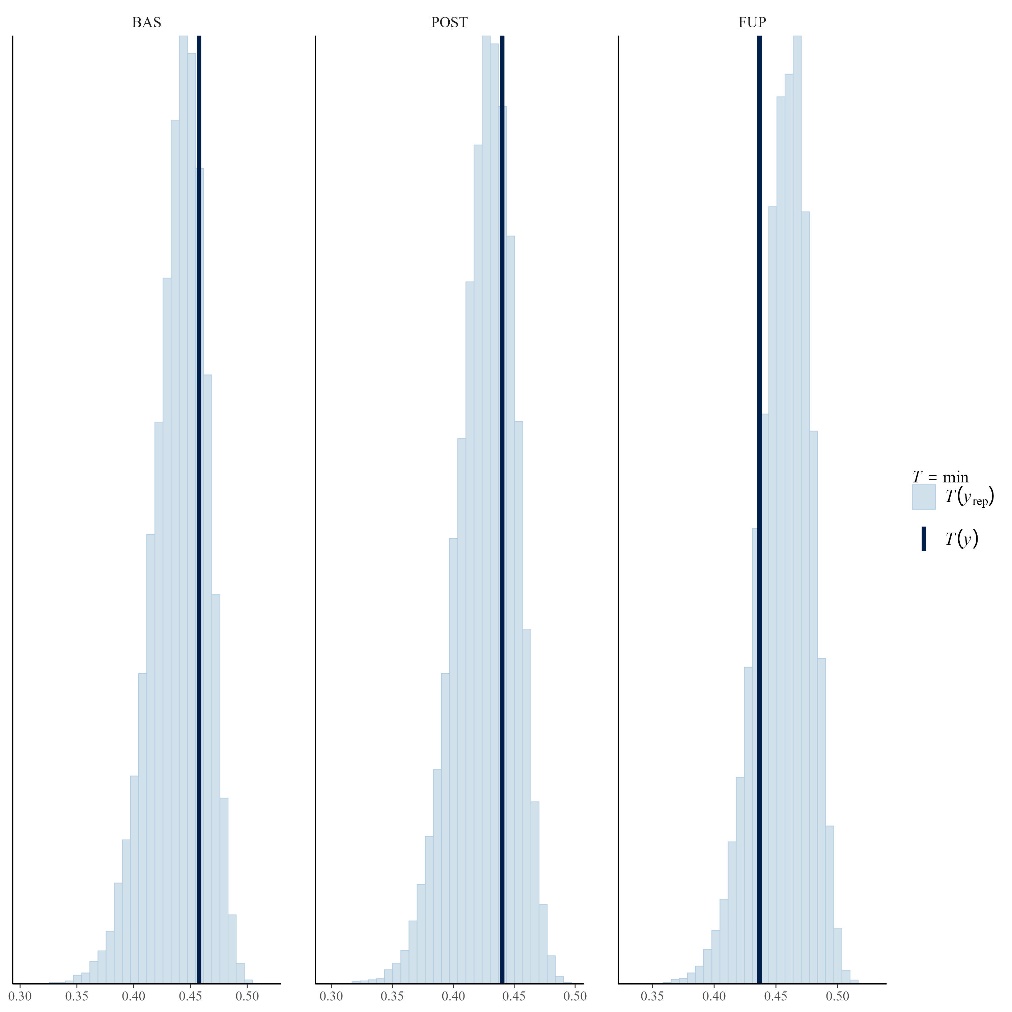


Figure S17d. Histogram plot comparing observed versus simulated Multi-Scale Entropy minimum values across responders from the posterior predictive distribution.


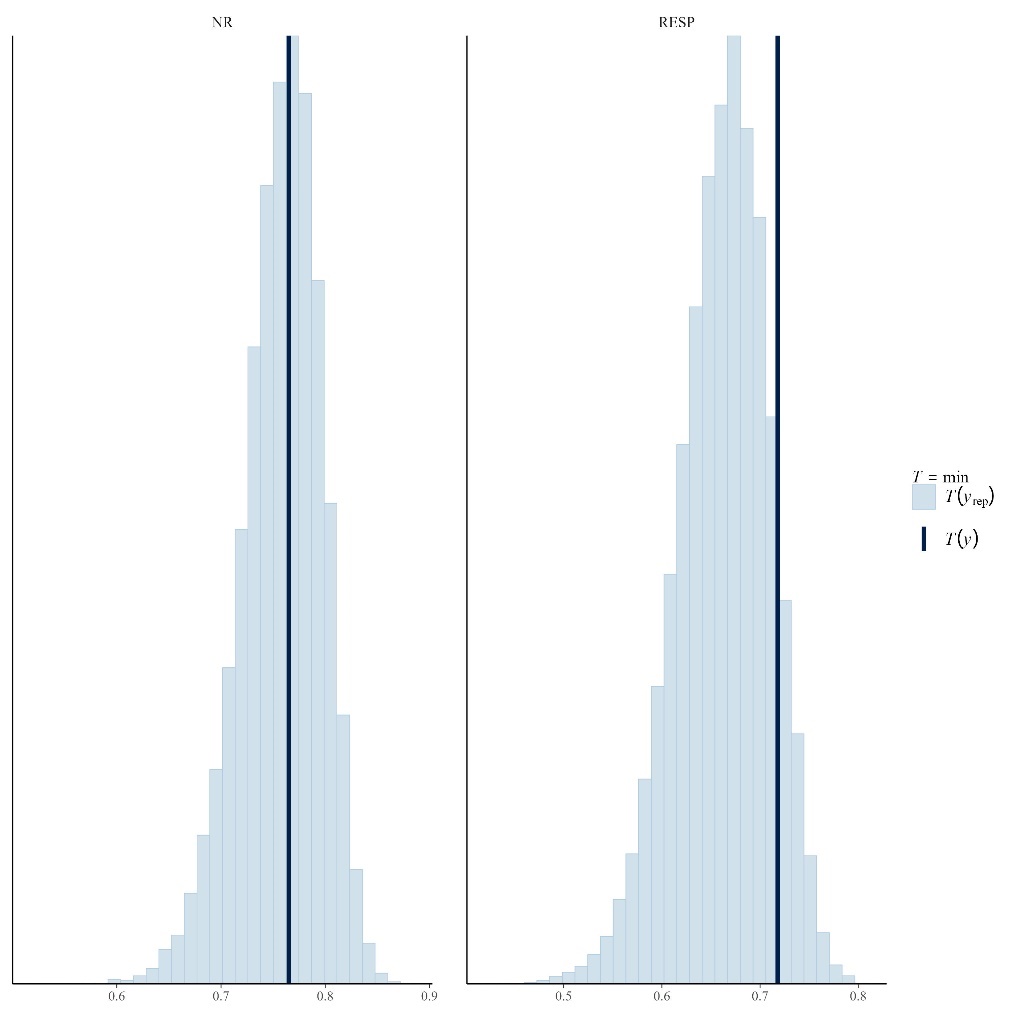


Figure S17e. Histogram plot comparing observed versus simulated Multi-Scale Entropy minimum values across tasks from the posterior predictive distribution.


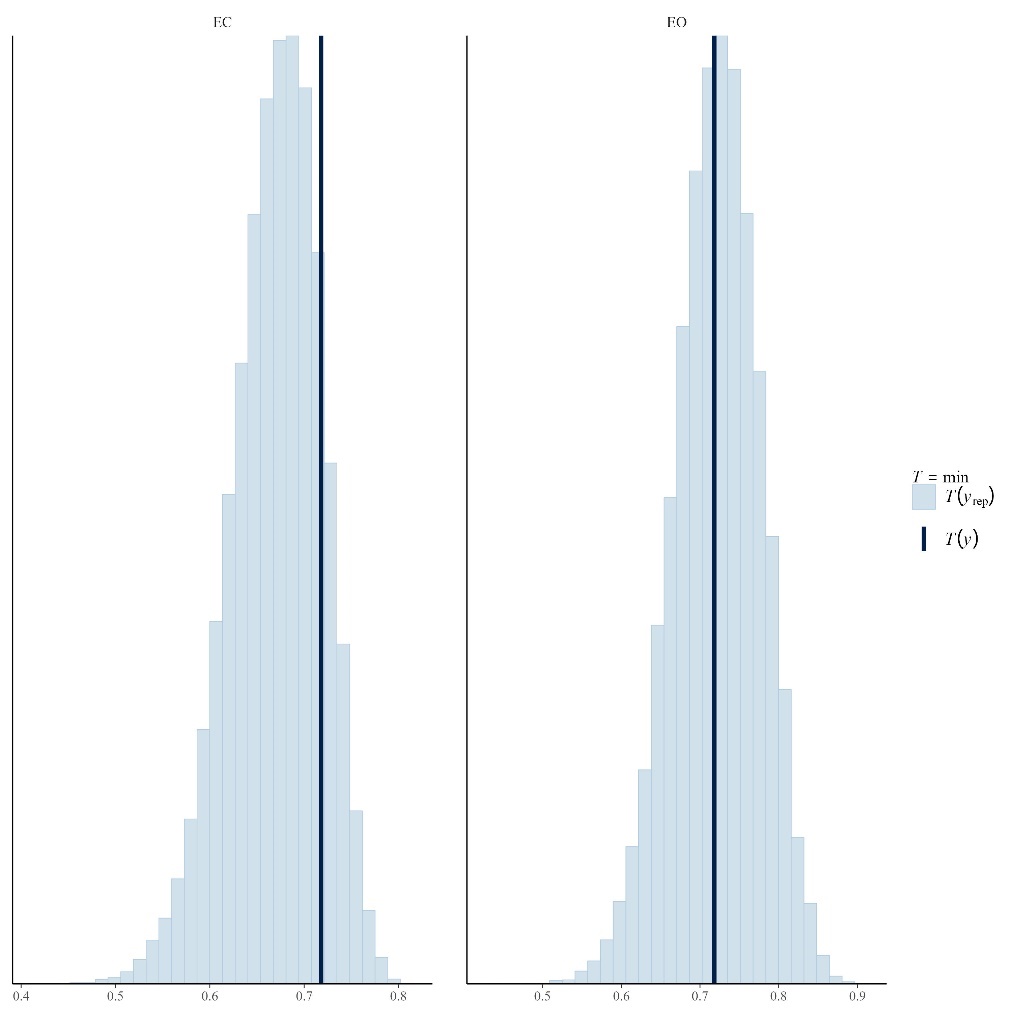


Figure S17f. Histogram plot comparing observed versus simulated Multi-Scale Entropy minimum values across timepoints from the posterior predictive distribution.


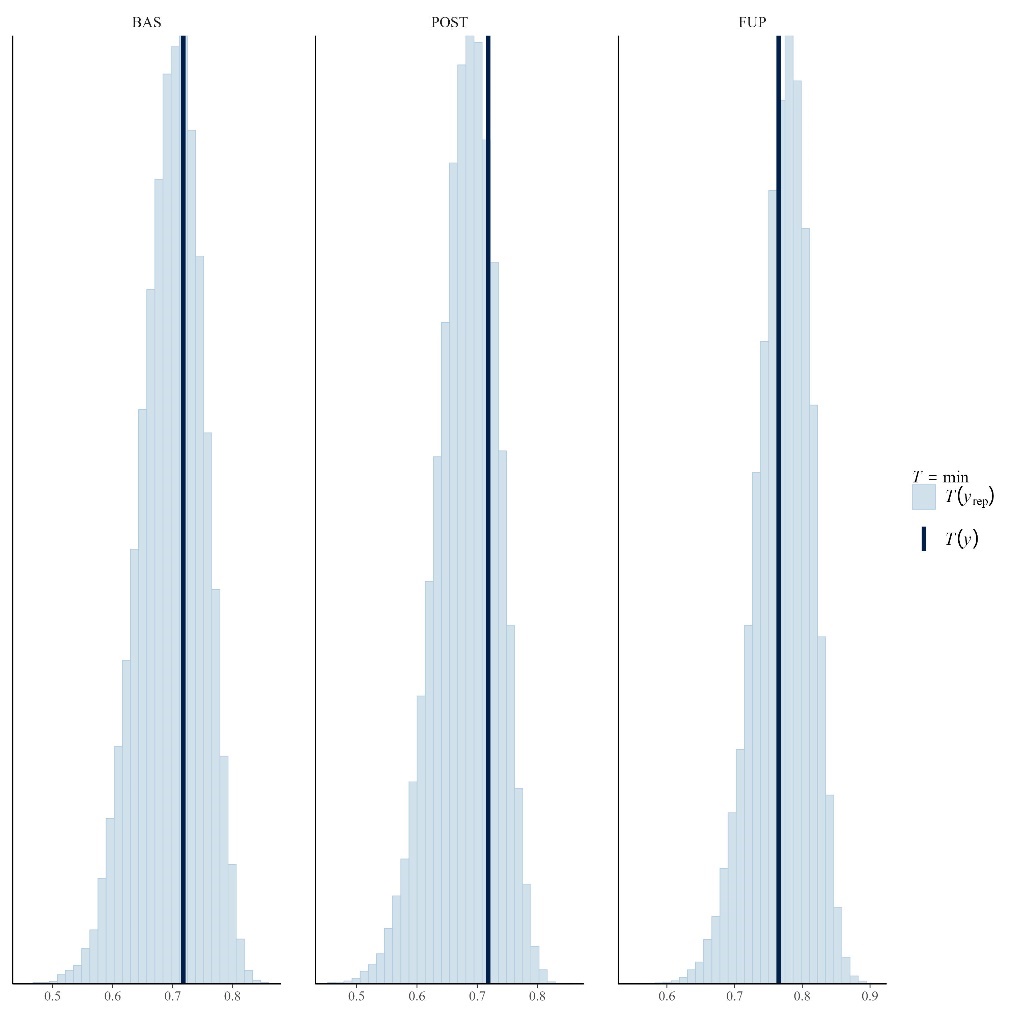


**Mean Values**

Figure S18a. Histogram plot comparing observed versus simulated Lempel-Ziv Complexity mean values across responders from the posterior predictive distribution.


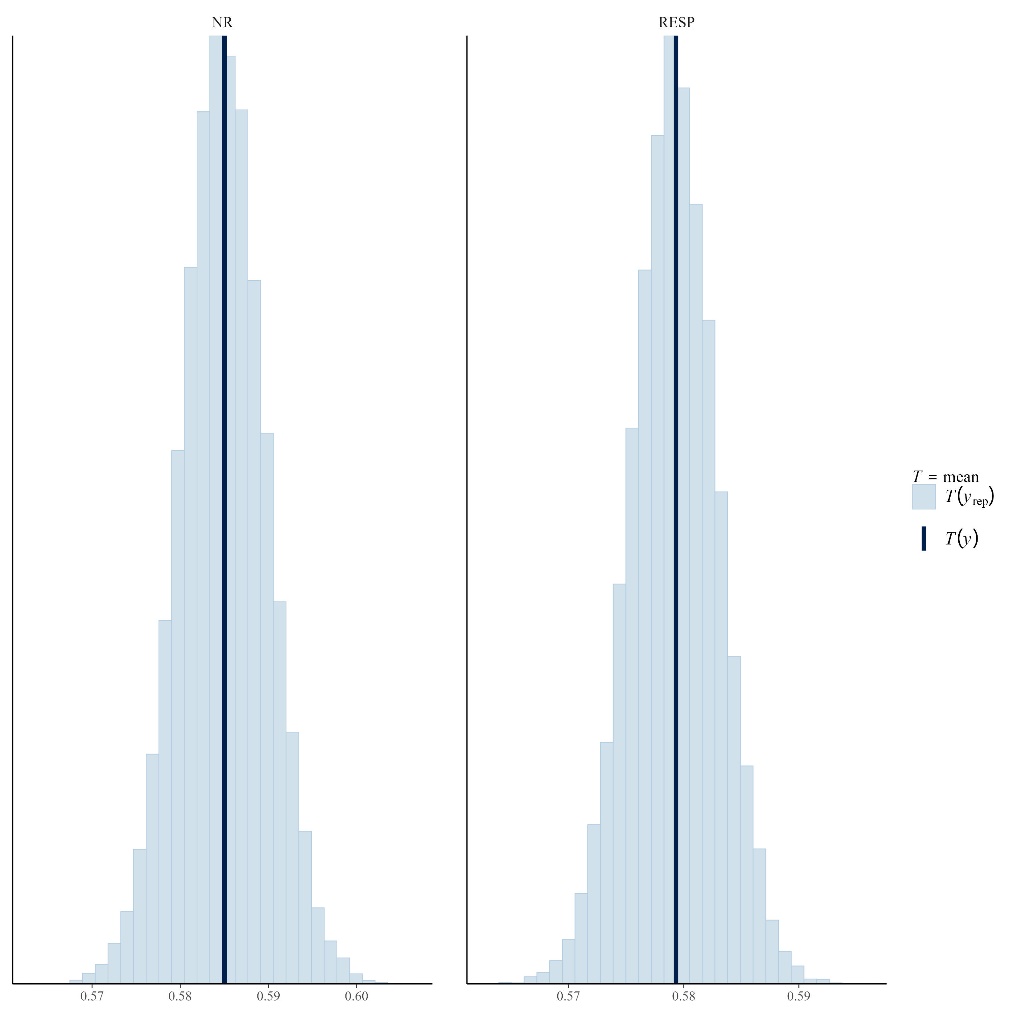


Figure S18b. Histogram plot comparing observed versus simulated Lempel-Ziv Complexity mean values across tasks from the posterior predictive distribution.


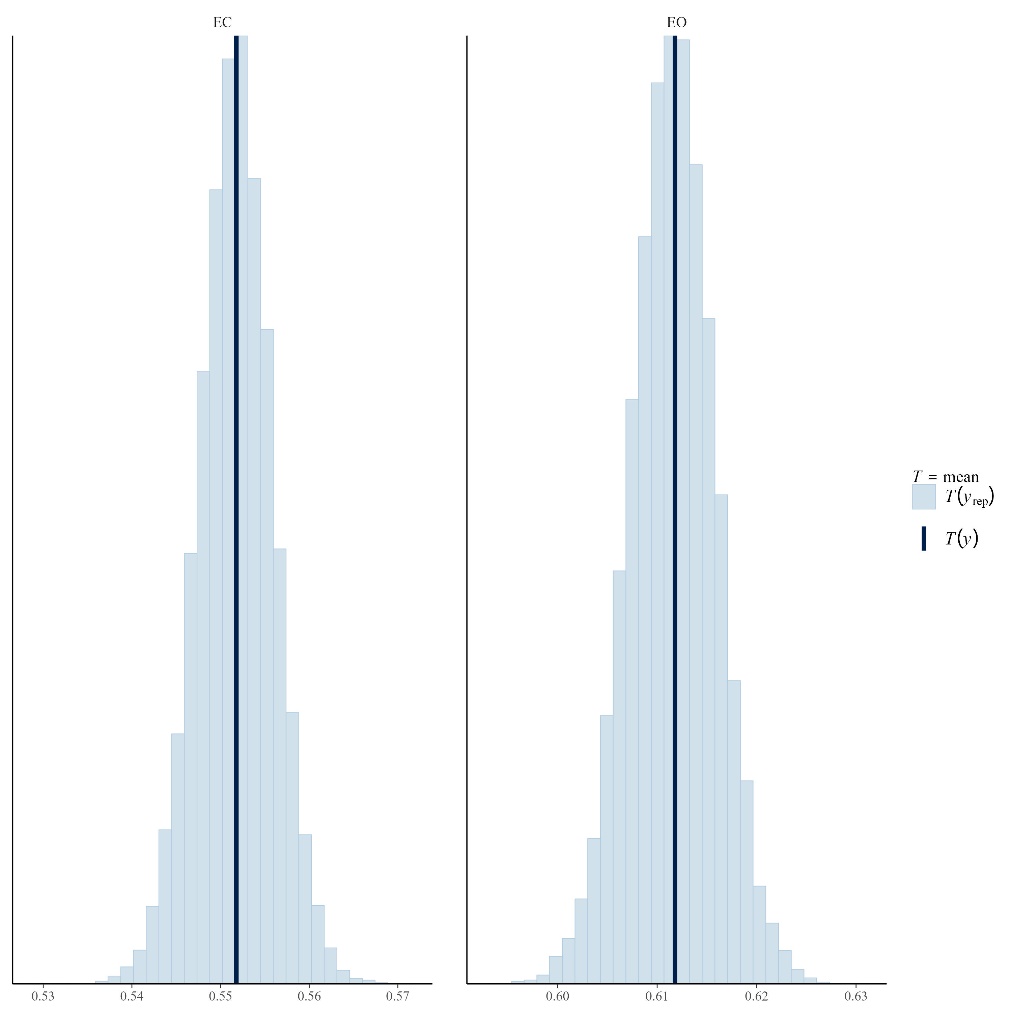


Figure S18c. Histogram plot comparing observed versus simulated Lempel-Ziv Complexity mean values across timepoints from the posterior predictive distribution.


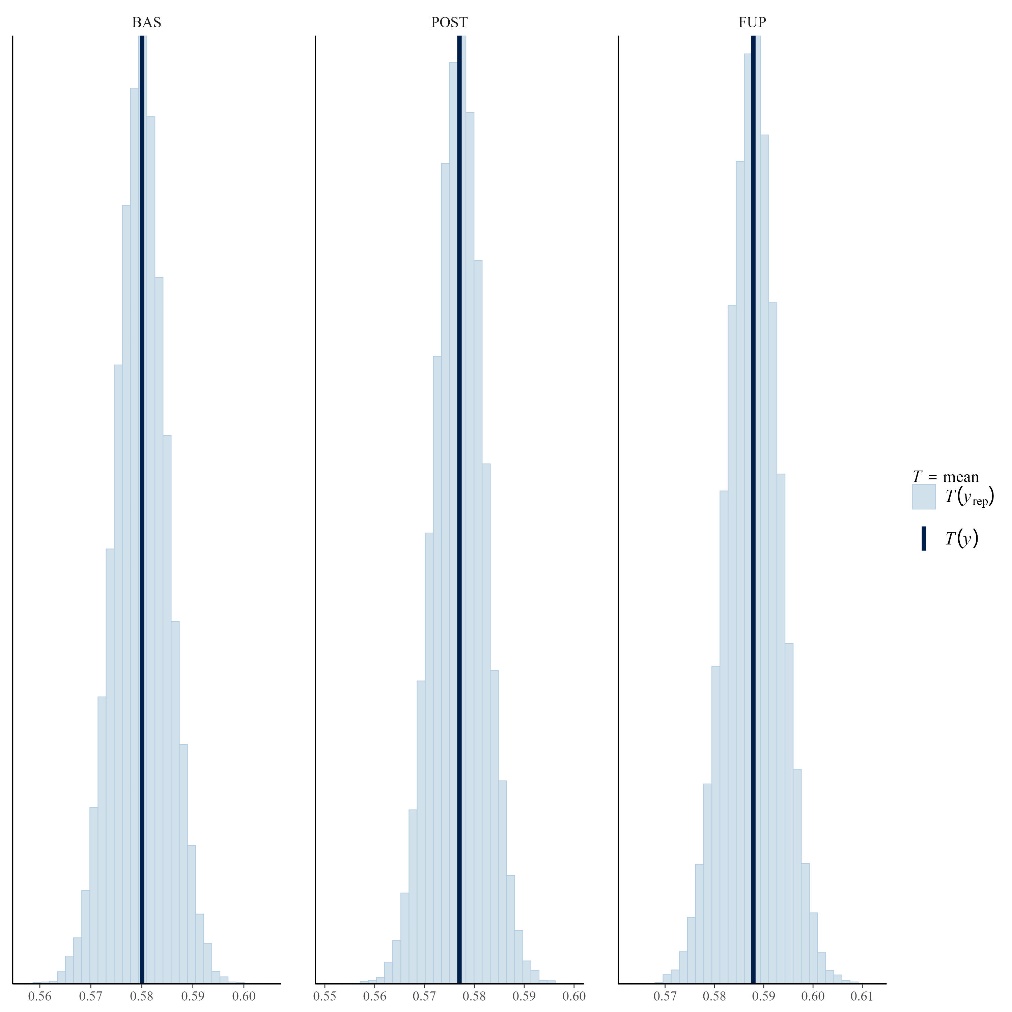


Figure S18d. Histogram plot comparing observed versus simulated Multi-Scale Entropy mean values across responders from the posterior predictive distribution.


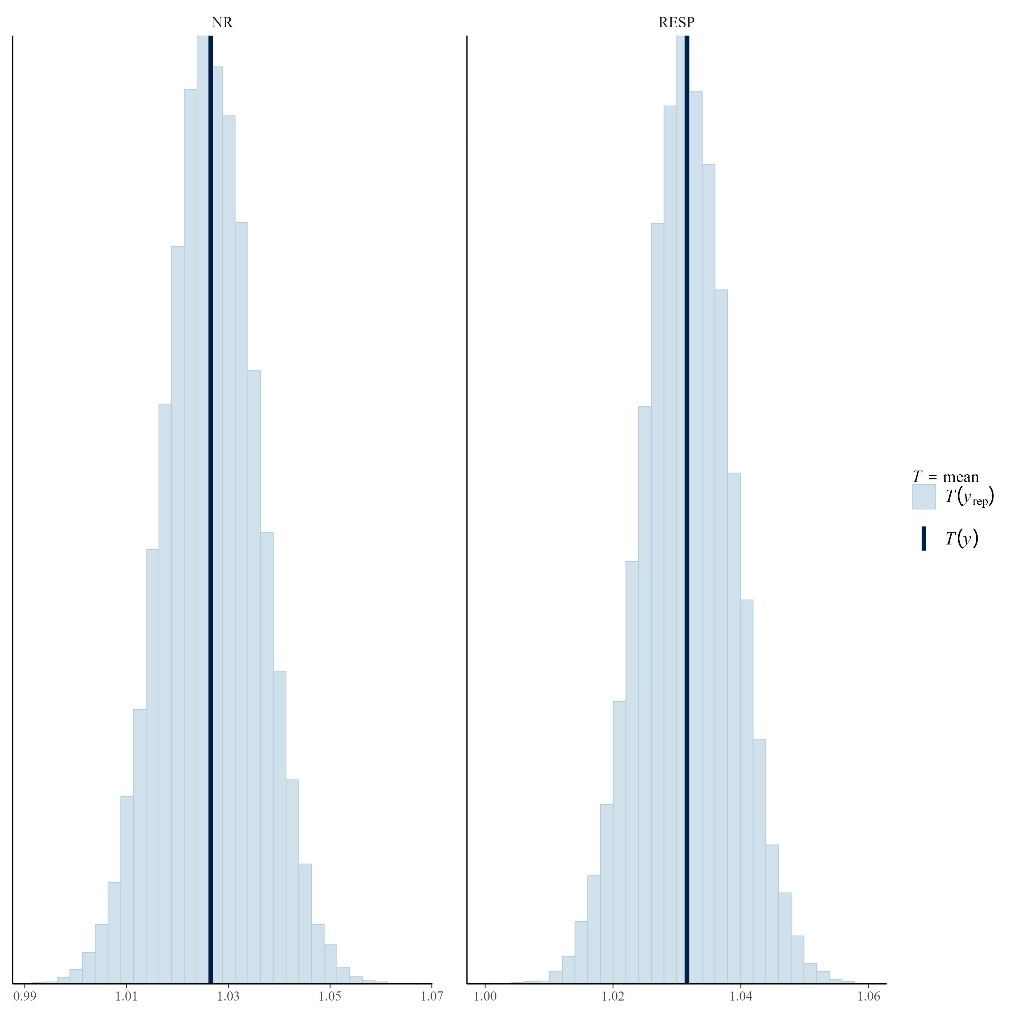


Figure S18e. Histogram plot comparing observed versus simulated Multi-Scale Entropy mean values across task from the posterior predictive distribution.


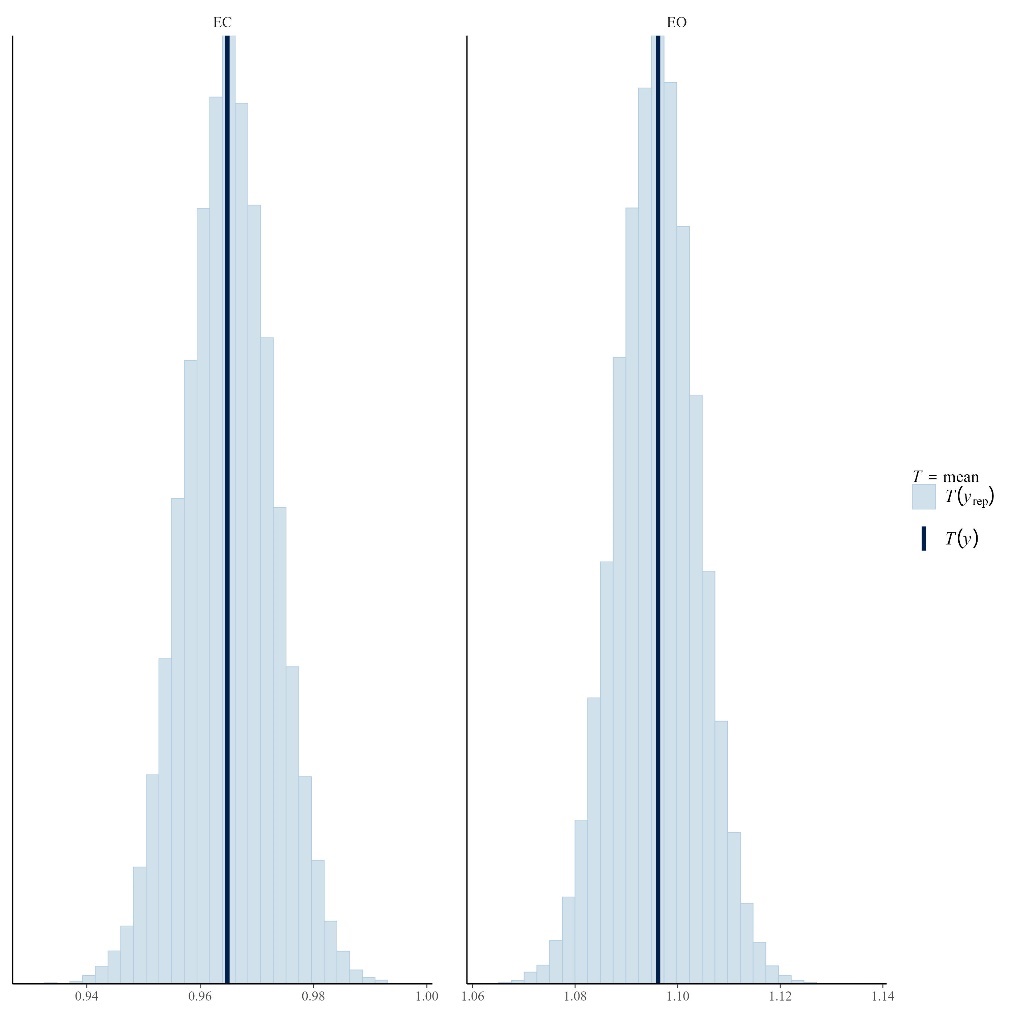


Figure S18f. Histogram plot comparing observed versus simulated Multi-Scale Entropy mean values across timepoint from the posterior predictive distribution.


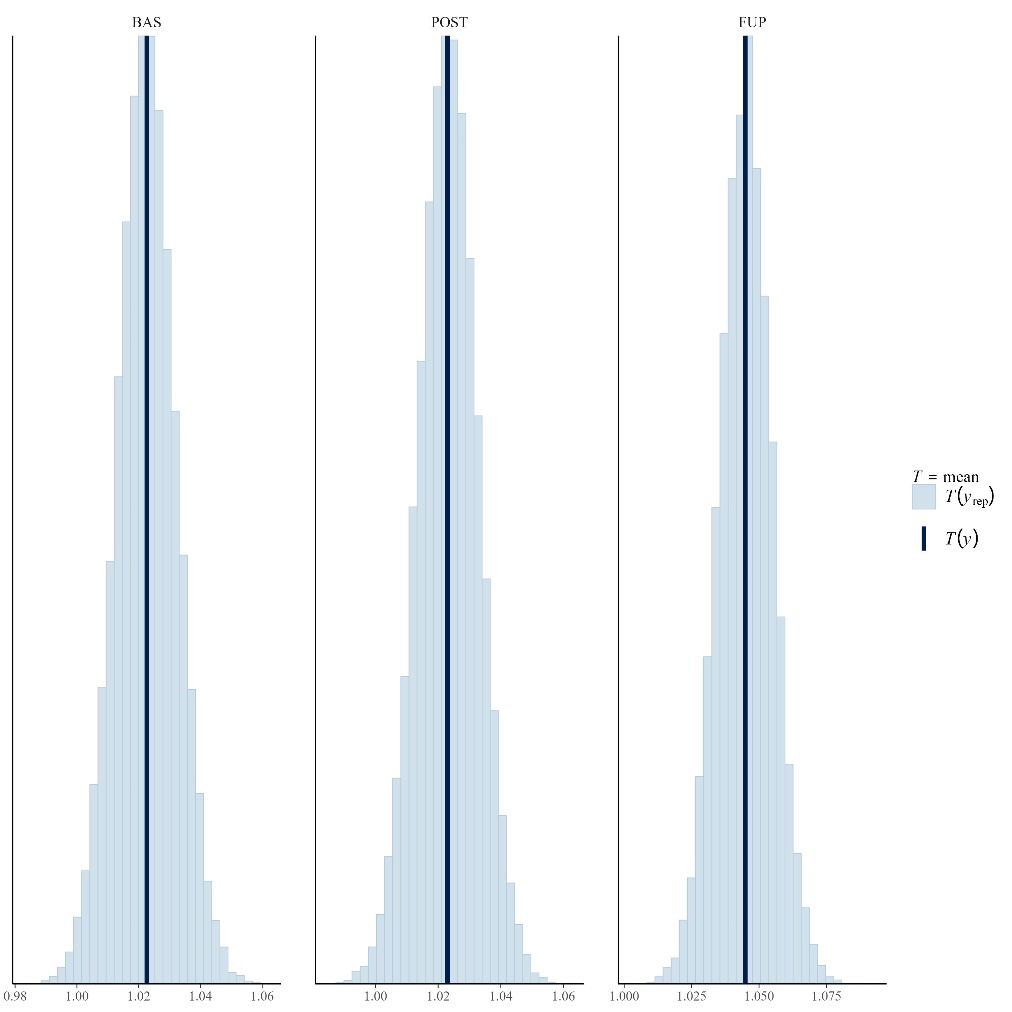


**Maximum Values**

Figure S19a. Histogram plot comparing observed versus simulated Lempel-Ziv Complexity maximum values across responders from the posterior predictive distribution.


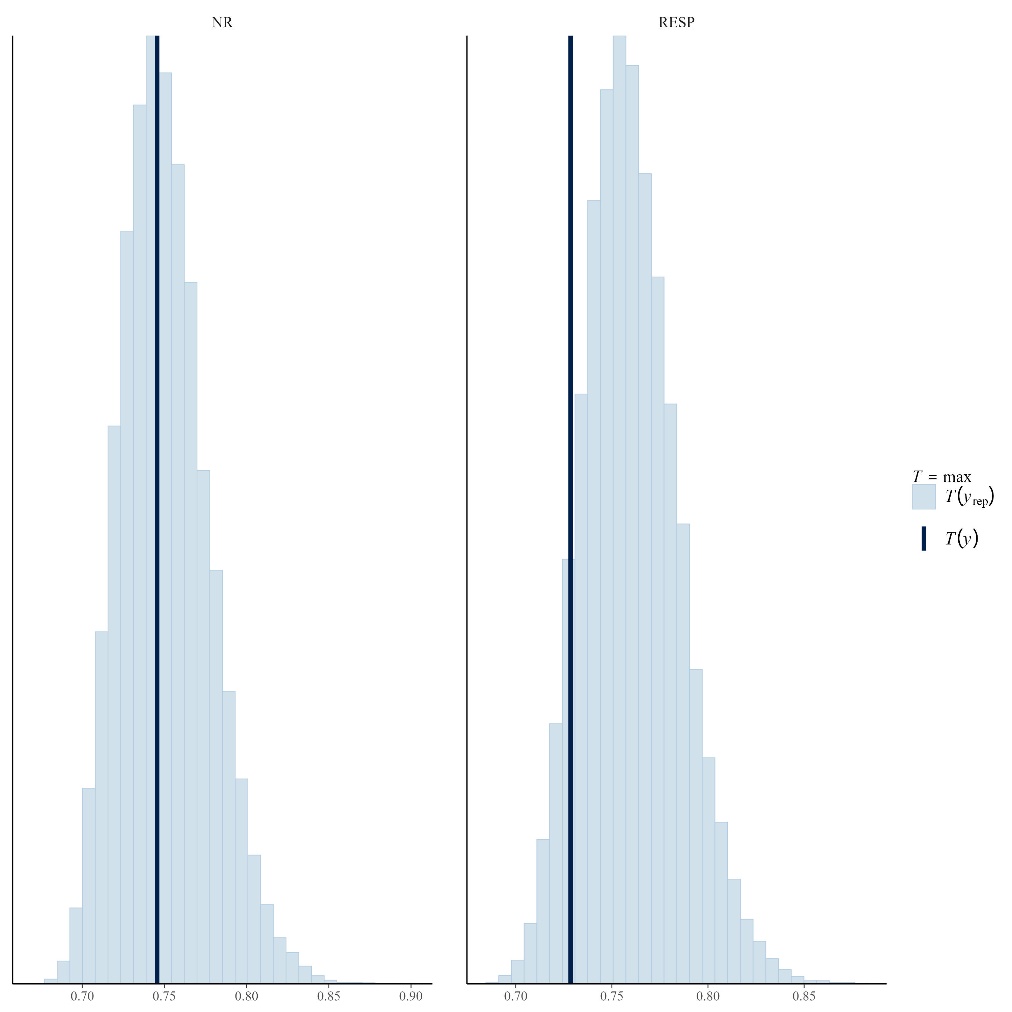


Figure S19b. Histogram plot comparing observed versus simulated Lempel-Ziv Complexity maximum values across tasks from the posterior predictive distribution.


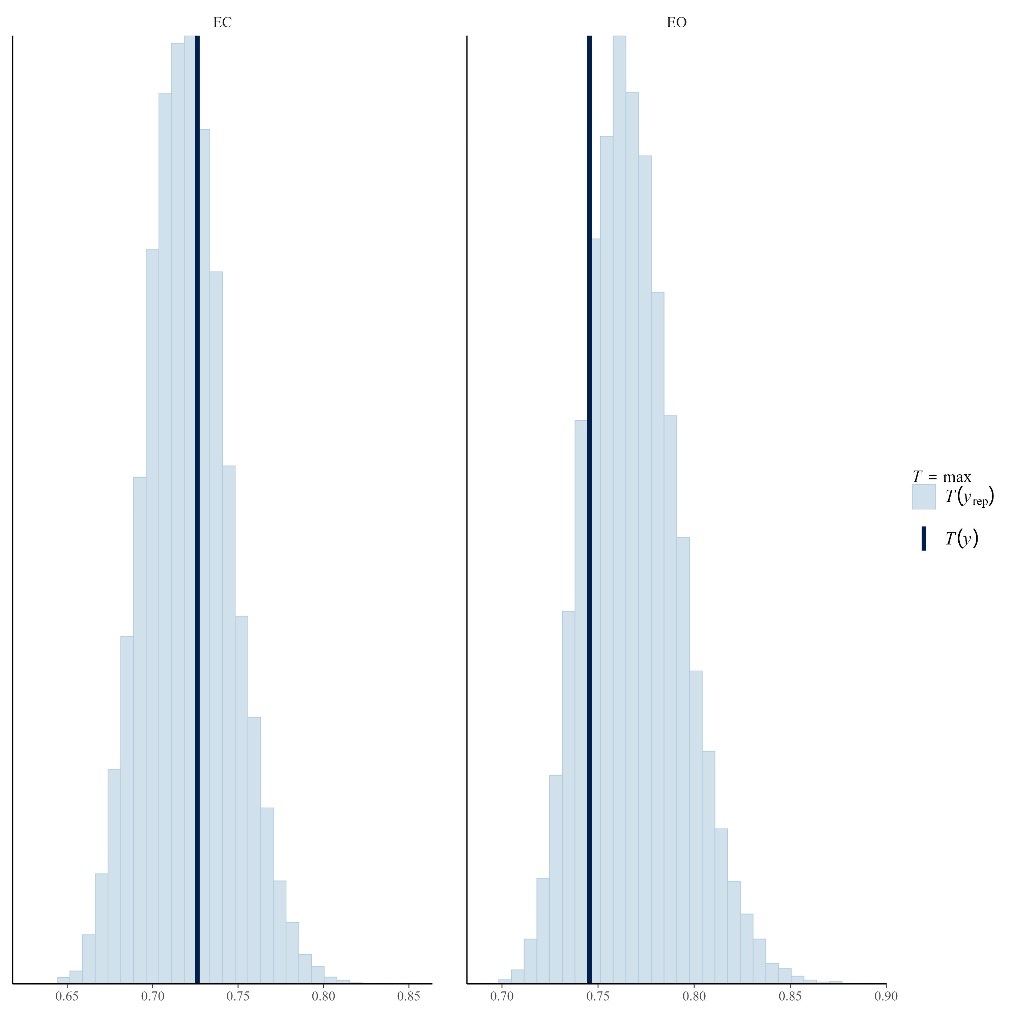


Figure S19c. Histogram plot comparing observed versus simulated Lempel-Ziv Complexity maximum values across timepoints from the posterior predictive distribution.


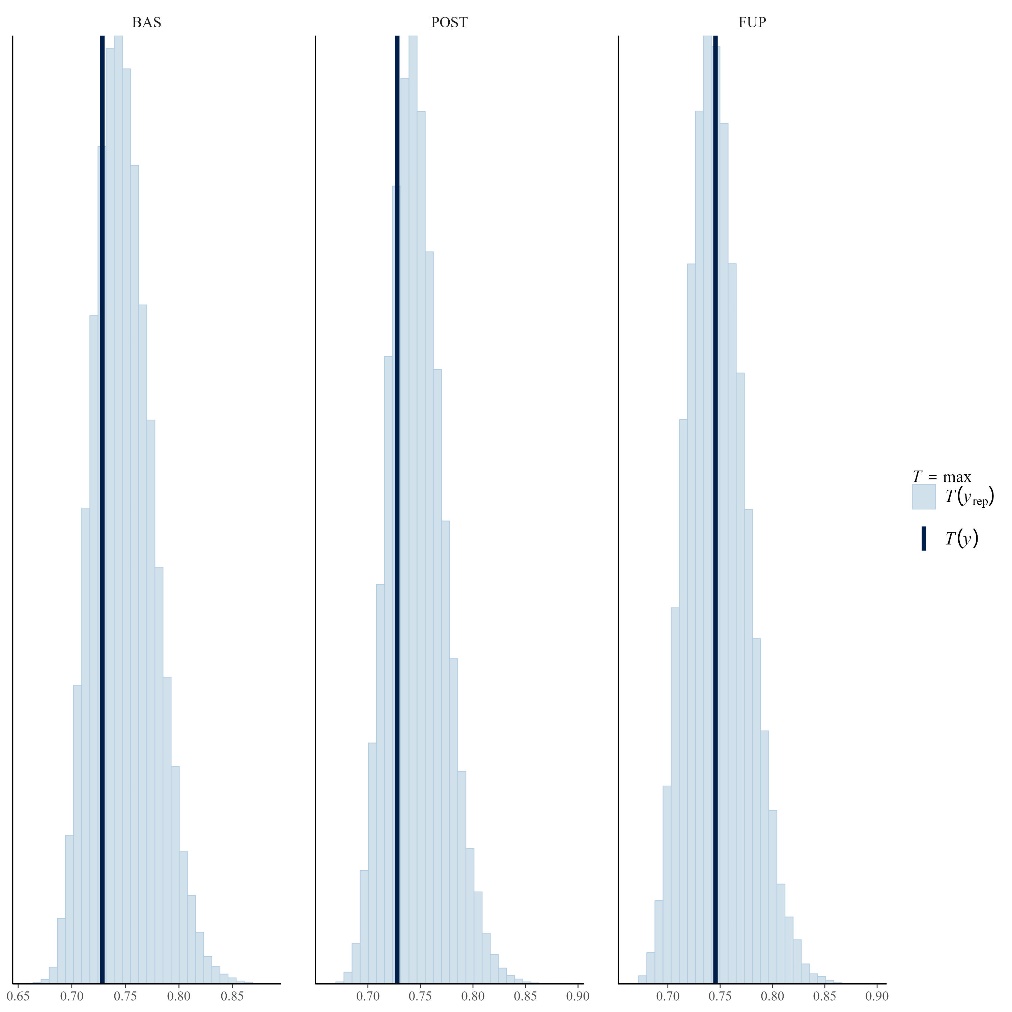


Figure S19d. Histogram plot comparing observed versus simulated Multi-Scale Entropy maximum values across responders from the posterior predictive distribution.


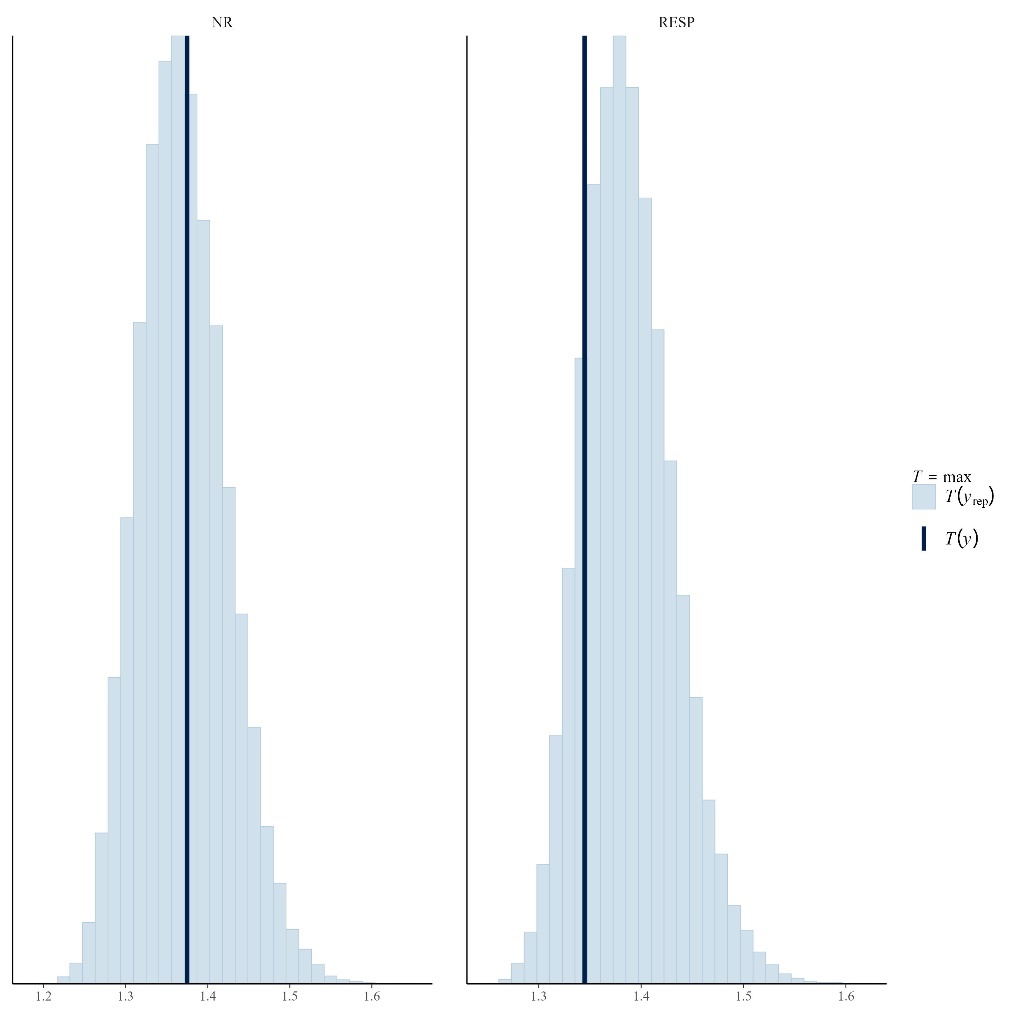


Figure S19e. Histogram plot comparing observed versus simulated Multi-Scale Entropy maximum values across task from the posterior predictive distribution.


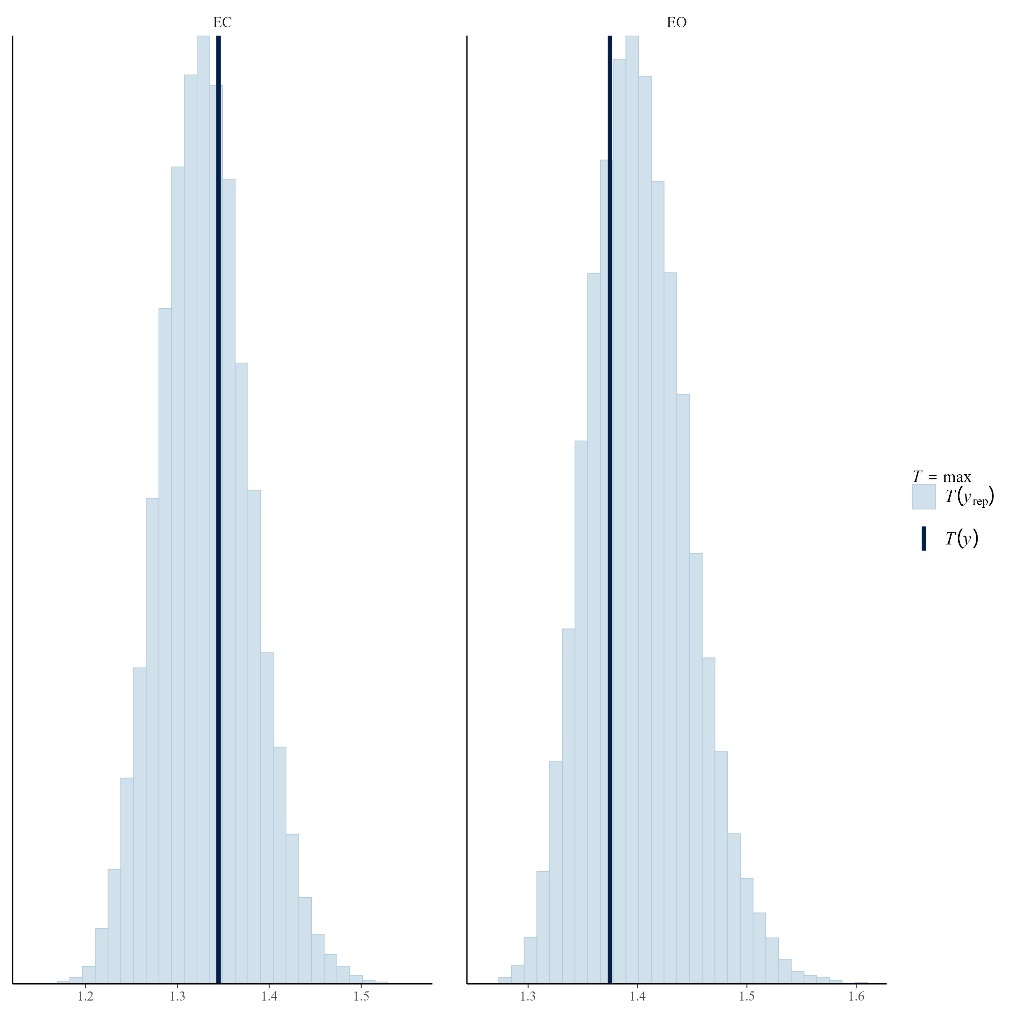


Figure S19f. Histogram plot comparing observed versus simulated Multi-Scale Entropy maximum values across timepoint from the posterior predictive distribution.


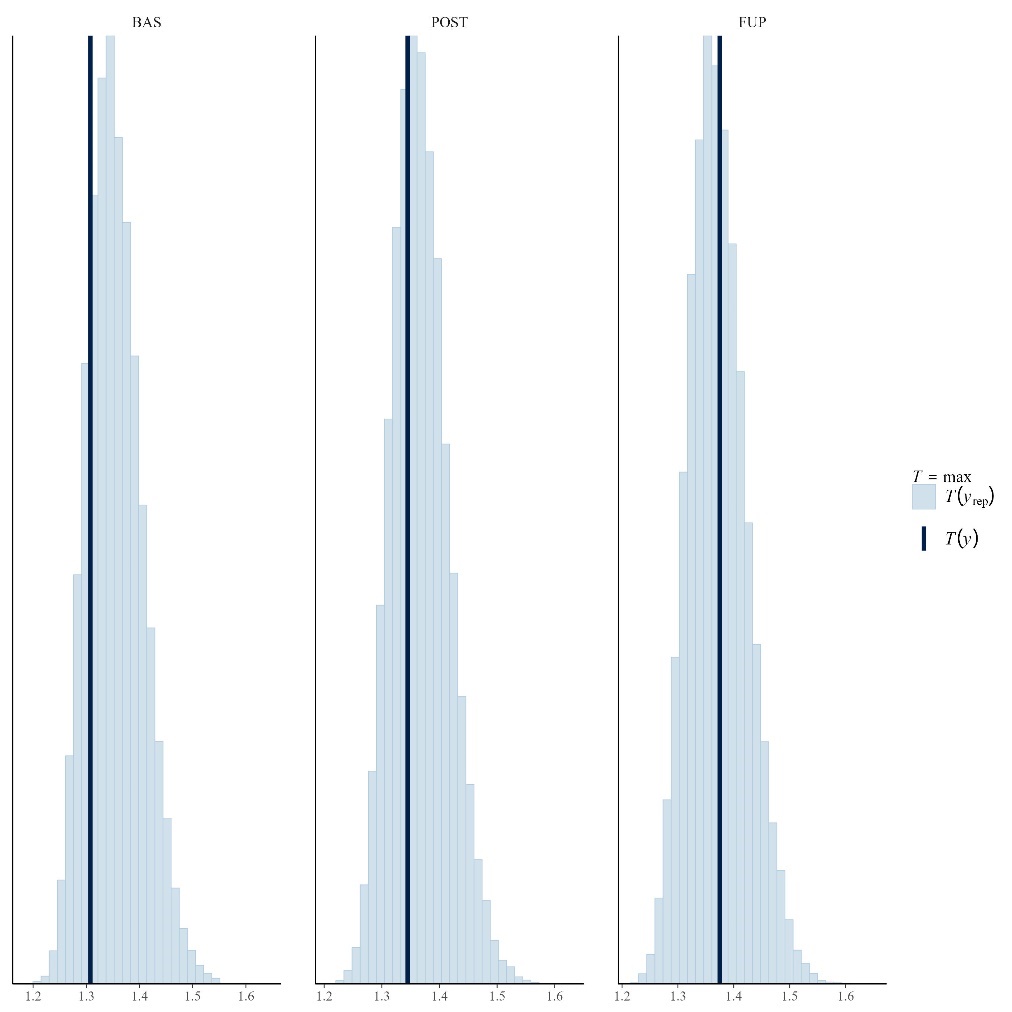


**Supplementary F. Effect Estimates**

Table S8. Posterior Distribution Summary for Lempel-Ziv Complexity. Note: Credible Intervals, CI; Probability of Direction, pd.

|  | Median | CI_low (2.5%) | CI_high (97.5%) | pd |
| --- | --- | --- | --- | --- |
| Intercept | 0.549 | 0.519 | 0.578 | 1 |
| ResponderRESP | 0.004 | -0.027 | 0.035 | 0.605 |
| TimepointPOST | -0.017 | -0.045 | 0.01 | 0.899 |
| TimepointFUP | -0.01 | -0.038 | 0.019 | 0.757 |
| TaskEO | 0.037 | 0.01 | 0.065 | 0.995 |
| ResponderRESP:TimepointPOST | 0.016 | -0.018 | 0.048 | 0.827 |
| ResponderRESP:TimepointFUP | 0.02 | -0.018 | 0.057 | 0.849 |
| ResponderRESP:TaskEO | 0.027 | -0.006 | 0.059 | 0.95 |
| TimepointPOST:TaskEO | 0.029 | -0.006 | 0.062 | 0.951 |
| TimepointFUP:TaskEO | 0.037 | 0.004 | 0.069 | 0.985 |
| ResponderRESP:TimepointPOST:TaskEO | -0.041 | -0.081 | 0.001 | 0.973 |
| ResponderRESP:TimepointFUP:TaskEO | -0.045 | -0.089 | -0.002 | 0.98 |

Table S9. Posterior Distribution Summary Multi-Scale Entropy. Note: Credible Intervals, CI; Probability of Direction, pd.

| Parameter | Median | CI_low (2.5%) | CI_high (97.5%) | pd |
| --- | --- | --- | --- | --- |
| Scale1_Intercept | 0.957 | 0.906 | 1.006 | 1 |
| Scale1_ResponderRESP | 0.013 | -0.037 | 0.063 | 0.689 |
| Scale1_TimepointPOST | -0.03 | -0.077 | 0.018 | 0.887 |
| Scale1_TimepointFUP | -0.007 | -0.054 | 0.042 | 0.617 |
| Scale1_TaskEO | 0.097 | 0.048 | 0.144 | 1 |
| Scale1_ResponderRESP:TimepointPOST | 0.037 | -0.021 | 0.091 | 0.895 |
| Scale1_ResponderRESP:TimepointFUP | 0.033 | -0.032 | 0.096 | 0.842 |
| Scale1_ResponderRESP:TaskEO | 0.042 | -0.013 | 0.097 | 0.932 |
| Scale1_TimepointPOST:TaskEO | 0.041 | -0.016 | 0.098 | 0.922 |
| Scale1_TimepointFUP:TaskEO | 0.051 | -0.004 | 0.106 | 0.966 |
| Scale1_ResponderRESP:TimepointPOST:TaskEO | -0.069 | -0.136 | 0 | 0.975 |
| Scale1_ResponderRESP:TimepointFUP:TaskEO | -0.071 | -0.143 | 0.002 | 0.972 |
| Scale2_Intercept | 1.551 | 1.499 | 1.602 | 1 |
| Scale2_ResponderRESP | 0.024 | -0.027 | 0.075 | 0.822 |
| Scale2_TimepointPOST | -0.027 | -0.074 | 0.02 | 0.868 |
| Scale2_TimepointFUP | -0.006 | -0.055 | 0.043 | 0.594 |
| Scale2_TaskEO | 0.105 | 0.058 | 0.153 | 1 |
| Scale2_ResponderRESP:TimepointPOST | 0.029 | -0.027 | 0.085 | 0.85 |
| Scale2_ResponderRESP:TimepointFUP | 0.026 | -0.04 | 0.09 | 0.776 |
| Scale2_ResponderRESP:TaskEO | 0.044 | -0.011 | 0.097 | 0.944 |
| Scale2_TimepointPOST:TaskEO | 0.046 | -0.009 | 0.1 | 0.95 |
| Scale2_TimepointFUP:TaskEO | 0.047 | -0.006 | 0.101 | 0.959 |
| Scale2_ResponderRESP:TimepointPOST:TaskEO | -0.078 | -0.143 | -0.012 | 0.99 |
| Scale2_ResponderRESP:TimepointFUP:TaskEO | -0.065 | -0.136 | 0.005 | 0.966 |
| Scale3_Intercept | 1.713 | 1.676 | 1.749 | 1 |
| Scale3_ResponderRESP | 0.026 | -0.011 | 0.063 | 0.92 |
| Scale3_TimepointPOST | -0.015 | -0.046 | 0.016 | 0.83 |
| Scale3_TimepointFUP | -0.001 | -0.037 | 0.033 | 0.53 |
| Scale3_TaskEO | 0.063 | 0.031 | 0.094 | 1 |
| Scale3_ResponderRESP:TimepointPOST | 0.009 | -0.027 | 0.046 | 0.693 |
| Scale3_ResponderRESP:TimepointFUP | 0.005 | -0.043 | 0.053 | 0.581 |
| Scale3_ResponderRESP:TaskEO | 0.024 | -0.011 | 0.059 | 0.912 |
| Scale3_TimepointPOST:TaskEO | 0.033 | -0.001 | 0.067 | 0.972 |
| Scale3_TimepointFUP:TaskEO | 0.022 | -0.011 | 0.055 | 0.902 |
| Scale3_ResponderRESP:TimepointPOST:TaskEO | -0.057 | -0.097 | -0.016 | 0.997 |
| Scale3_ResponderRESP:TimepointFUP:TaskEO | -0.032 | -0.077 | 0.013 | 0.922 |
| Scale4_Intercept | 1.77 | 1.742 | 1.798 | 1 |
| Scale4_ResponderRESP | 0.022 | -0.007 | 0.05 | 0.936 |
| Scale4_TimepointPOST | -0.001 | -0.024 | 0.022 | 0.537 |
| Scale4_TimepointFUP | 0.001 | -0.026 | 0.029 | 0.535 |
| Scale4_TaskEO | 0.028 | 0.007 | 0.05 | 0.995 |
| Scale4_ResponderRESP:TimepointPOST | -0.009 | -0.036 | 0.019 | 0.738 |
| Scale4_ResponderRESP:TimepointFUP | -0.009 | -0.046 | 0.028 | 0.691 |
| Scale4_ResponderRESP:TaskEO | 0.007 | -0.018 | 0.031 | 0.703 |
| Scale4_TimepointPOST:TaskEO | 0.019 | -0.004 | 0.041 | 0.947 |
| Scale4_TimepointFUP:TaskEO | 0.006 | -0.017 | 0.029 | 0.697 |
| Scale4_ResponderRESP:TimepointPOST:TaskEO | -0.034 | -0.061 | -0.006 | 0.99 |
| Scale4_ResponderRESP:TimepointFUP:TaskEO | -0.008 | -0.039 | 0.022 | 0.705 |
| Scale5_Intercept | 1.801 | 1.776 | 1.825 | 1 |
| Scale5_ResponderRESP | 0.018 | -0.007 | 0.044 | 0.923 |
| Scale5_TimepointPOST | 0.006 | -0.017 | 0.029 | 0.713 |
| Scale5_TimepointFUP | 0.004 | -0.021 | 0.028 | 0.611 |
| Scale5_TaskEO | -0.002 | -0.022 | 0.018 | 0.559 |
| Scale5_ResponderRESP:TimepointPOST | -0.019 | -0.046 | 0.008 | 0.921 |
| Scale5_ResponderRESP:TimepointFUP | -0.017 | -0.05 | 0.016 | 0.845 |
| Scale5_ResponderRESP:TaskEO | -0.007 | -0.029 | 0.016 | 0.713 |
| Scale5_TimepointPOST:TaskEO | 0.01 | -0.011 | 0.032 | 0.827 |
| Scale5_TimepointFUP:TaskEO | -0.005 | -0.026 | 0.017 | 0.664 |
| Scale5_ResponderRESP:TimepointPOST:TaskEO | -0.017 | -0.043 | 0.009 | 0.904 |
| Scale5_ResponderRESP:TimepointFUP:TaskEO | 0.004 | -0.025 | 0.032 | 0.602 |
| Scale6_Intercept | 1.815 | 1.79 | 1.838 | 1 |
| Scale6_ResponderRESP | 0.016 | -0.009 | 0.041 | 0.893 |
| Scale6_TimepointPOST | 0.014 | -0.01 | 0.039 | 0.875 |
| Scale6_TimepointFUP | 0.007 | -0.018 | 0.031 | 0.714 |
| Scale6_TaskEO | -0.022 | -0.043 | -0.002 | 0.982 |
| Scale6_ResponderRESP:TimepointPOST | -0.028 | -0.057 | 0 | 0.974 |
| Scale6_ResponderRESP:TimepointFUP | -0.021 | -0.055 | 0.012 | 0.9 |
| Scale6_ResponderRESP:TaskEO | -0.015 | -0.038 | 0.009 | 0.896 |
| Scale6_TimepointPOST:TaskEO | 0.004 | -0.019 | 0.028 | 0.651 |
| Scale6_TimepointFUP:TaskEO | -0.01 | -0.032 | 0.013 | 0.804 |
| Scale6_ResponderRESP:TimepointPOST:TaskEO | -0.006 | -0.034 | 0.022 | 0.671 |
| Scale6_ResponderRESP:TimepointFUP:TaskEO | 0.01 | -0.02 | 0.04 | 0.744 |
| Scale7_Intercept | 1.826 | 1.801 | 1.85 | 1 |
| Scale7_ResponderRESP | 0.012 | -0.014 | 0.038 | 0.819 |
| Scale7_TimepointPOST | 0.013 | -0.013 | 0.039 | 0.834 |
| Scale7_TimepointFUP | 0.007 | -0.018 | 0.033 | 0.706 |
| Scale7_TaskEO | -0.041 | -0.063 | -0.02 | 1 |
| Scale7_ResponderRESP:TimepointPOST | -0.028 | -0.058 | 0.003 | 0.962 |
| Scale7_ResponderRESP:TimepointFUP | -0.026 | -0.061 | 0.009 | 0.93 |
| Scale7_ResponderRESP:TaskEO | -0.018 | -0.043 | 0.007 | 0.923 |
| Scale7_TimepointPOST:TaskEO | 0.005 | -0.02 | 0.03 | 0.646 |
| Scale7_TimepointFUP:TaskEO | -0.008 | -0.032 | 0.016 | 0.748 |
| Scale7_ResponderRESP:TimepointPOST:TaskEO | -0.004 | -0.035 | 0.026 | 0.611 |
| Scale7_ResponderRESP:TimepointFUP:TaskEO | 0.011 | -0.022 | 0.042 | 0.74 |
| Scale8_Intercept | 1.825 | 1.798 | 1.852 | 1 |
| Scale8_ResponderRESP | 0.008 | -0.02 | 0.036 | 0.717 |
| Scale8_TimepointPOST | 0.016 | -0.013 | 0.044 | 0.861 |
| Scale8_TimepointFUP | 0.012 | -0.015 | 0.04 | 0.804 |
| Scale8_TaskEO | -0.052 | -0.076 | -0.028 | 1 |
| Scale8_ResponderRESP:TimepointPOST | -0.028 | -0.061 | 0.006 | 0.949 |
| Scale8_ResponderRESP:TimepointFUP | -0.029 | -0.066 | 0.009 | 0.933 |
| Scale8_ResponderRESP:TaskEO | -0.022 | -0.049 | 0.006 | 0.939 |
| Scale8_TimepointPOST:TaskEO | 0.003 | -0.025 | 0.03 | 0.585 |
| Scale8_TimepointFUP:TaskEO | -0.017 | -0.044 | 0.01 | 0.891 |
| Scale8_ResponderRESP:TimepointPOST:TaskEO | -0.001 | -0.034 | 0.033 | 0.522 |
| Scale8_ResponderRESP:TimepointFUP:TaskEO | 0.014 | -0.022 | 0.049 | 0.779 |
| Scale9_Intercept | 1.81 | 1.782 | 1.839 | 1 |
| Scale9_ResponderRESP | 0.013 | -0.018 | 0.042 | 0.798 |
| Scale9_TimepointPOST | 0.021 | -0.009 | 0.05 | 0.913 |
| Scale9_TimepointFUP | 0.02 | -0.009 | 0.049 | 0.91 |
| Scale9_TaskEO | -0.05 | -0.076 | -0.024 | 1 |
| Scale9_ResponderRESP:TimepointPOST | -0.03 | -0.065 | 0.006 | 0.953 |
| Scale9_ResponderRESP:TimepointFUP | -0.036 | -0.075 | 0.003 | 0.966 |
| Scale9_ResponderRESP:TaskEO | -0.036 | -0.066 | -0.006 | 0.99 |
| Scale9_TimepointPOST:TaskEO | -0.007 | -0.037 | 0.023 | 0.675 |
| Scale9_TimepointFUP:TaskEO | -0.027 | -0.056 | 0.002 | 0.967 |
| Scale9_ResponderRESP:TimepointPOST:TaskEO | 0.011 | -0.025 | 0.047 | 0.724 |
| Scale9_ResponderRESP:TimepointFUP:TaskEO | 0.027 | -0.012 | 0.065 | 0.915 |
| Scale10_Intercept | 1.796 | 1.764 | 1.828 | 1 |
| Scale10_ResponderRESP | 0.006 | -0.028 | 0.039 | 0.635 |
| Scale10_TimepointPOST | 0.023 | -0.009 | 0.054 | 0.92 |
| Scale10_TimepointFUP | 0.019 | -0.013 | 0.05 | 0.881 |
| Scale10_TaskEO | -0.055 | -0.083 | -0.026 | 1 |
| Scale10_ResponderRESP:TimepointPOST | -0.029 | -0.067 | 0.008 | 0.939 |
| Scale10_ResponderRESP:TimepointFUP | -0.035 | -0.077 | 0.008 | 0.946 |
| Scale10_ResponderRESP:TaskEO | -0.028 | -0.061 | 0.005 | 0.953 |
| Scale10_TimepointPOST:TaskEO | -0.008 | -0.041 | 0.026 | 0.668 |
| Scale10_TimepointFUP:TaskEO | -0.027 | -0.059 | 0.006 | 0.946 |
| Scale10_ResponderRESP:TimepointPOST:TaskEO | 0.01 | -0.031 | 0.05 | 0.68 |
| Scale10_ResponderRESP:TimepointFUP:TaskEO | 0.024 | -0.019 | 0.067 | 0.862 |

Table S10. Posterior Distribution Summary for Channel-Level Lempel-Ziv Complexity. Note: Credible Intervals, 95% CI; Probability of Direction, pd.

| Parameter | Median | CI_low (2.5%) | CI_high (97.5%) | pd |
| --- | --- | --- | --- | --- |
| Scale1_Intercept | 0.957 | 0.906 | 1.006 | 1 |
| Scale1_ResponderRESP | 0.013 | -0.037 | 0.063 | 0.689 |
| Scale1_TimepointPOST | -0.03 | -0.077 | 0.018 | 0.887 |
| Scale1_TimepointFUP | -0.007 | -0.054 | 0.042 | 0.617 |
| Scale1_TaskEO | 0.097 | 0.048 | 0.144 | 1 |
| Scale1_ResponderRESP:TimepointPOST | 0.037 | -0.021 | 0.091 | 0.895 |
| Scale1_ResponderRESP:TimepointFUP | 0.033 | -0.032 | 0.096 | 0.842 |
| Scale1_ResponderRESP:TaskEO | 0.042 | -0.013 | 0.097 | 0.932 |
| Scale1_TimepointPOST:TaskEO | 0.041 | -0.016 | 0.098 | 0.922 |
| Scale1_TimepointFUP:TaskEO | 0.051 | -0.004 | 0.106 | 0.966 |
| Scale1_ResponderRESP:TimepointPOST:TaskEO | -0.069 | -0.136 | 0 | 0.975 |
| Scale1_ResponderRESP:TimepointFUP:TaskEO | -0.071 | -0.143 | 0.002 | 0.972 |
| Scale2_Intercept | 1.551 | 1.499 | 1.602 | 1 |
| Scale2_ResponderRESP | 0.024 | -0.027 | 0.075 | 0.822 |
| Scale2_TimepointPOST | -0.027 | -0.074 | 0.02 | 0.868 |
| Scale2_TimepointFUP | -0.006 | -0.055 | 0.043 | 0.594 |
| Scale2_TaskEO | 0.105 | 0.058 | 0.153 | 1 |
| Scale2_ResponderRESP:TimepointPOST | 0.029 | -0.027 | 0.085 | 0.85 |
| Scale2_ResponderRESP:TimepointFUP | 0.026 | -0.04 | 0.09 | 0.776 |
| Scale2_ResponderRESP:TaskEO | 0.044 | -0.011 | 0.097 | 0.944 |
| Scale2_TimepointPOST:TaskEO | 0.046 | -0.009 | 0.1 | 0.95 |
| Scale2_TimepointFUP:TaskEO | 0.047 | -0.006 | 0.101 | 0.959 |
| Scale2_ResponderRESP:TimepointPOST:TaskEO | -0.078 | -0.143 | -0.012 | 0.99 |
| Scale2_ResponderRESP:TimepointFUP:TaskEO | -0.065 | -0.136 | 0.005 | 0.966 |
| Scale3_Intercept | 1.713 | 1.676 | 1.749 | 1 |
| Scale3_ResponderRESP | 0.026 | -0.011 | 0.063 | 0.92 |
| Scale3_TimepointPOST | -0.015 | -0.046 | 0.016 | 0.83 |
| Scale3_TimepointFUP | -0.001 | -0.037 | 0.033 | 0.53 |
| Scale3_TaskEO | 0.063 | 0.031 | 0.094 | 1 |
| Scale3_ResponderRESP:TimepointPOST | 0.009 | -0.027 | 0.046 | 0.693 |
| Scale3_ResponderRESP:TimepointFUP | 0.005 | -0.043 | 0.053 | 0.581 |
| Scale3_ResponderRESP:TaskEO | 0.024 | -0.011 | 0.059 | 0.912 |
| Scale3_TimepointPOST:TaskEO | 0.033 | -0.001 | 0.067 | 0.972 |
| Scale3_TimepointFUP:TaskEO | 0.022 | -0.011 | 0.055 | 0.902 |
| Scale3_ResponderRESP:TimepointPOST:TaskEO | -0.057 | -0.097 | -0.016 | 0.997 |
| Scale3_ResponderRESP:TimepointFUP:TaskEO | -0.032 | -0.077 | 0.013 | 0.922 |
| Scale4_Intercept | 1.77 | 1.742 | 1.798 | 1 |
| Scale4_ResponderRESP | 0.022 | -0.007 | 0.05 | 0.936 |
| Scale4_TimepointPOST | -0.001 | -0.024 | 0.022 | 0.537 |
| Scale4_TimepointFUP | 0.001 | -0.026 | 0.029 | 0.535 |
| Scale4_TaskEO | 0.028 | 0.007 | 0.05 | 0.995 |
| Scale4_ResponderRESP:TimepointPOST | -0.009 | -0.036 | 0.019 | 0.738 |
| Scale4_ResponderRESP:TimepointFUP | -0.009 | -0.046 | 0.028 | 0.691 |
| Scale4_ResponderRESP:TaskEO | 0.007 | -0.018 | 0.031 | 0.703 |
| Scale4_TimepointPOST:TaskEO | 0.019 | -0.004 | 0.041 | 0.947 |
| Scale4_TimepointFUP:TaskEO | 0.006 | -0.017 | 0.029 | 0.697 |
| Scale4_ResponderRESP:TimepointPOST:TaskEO | -0.034 | -0.061 | -0.006 | 0.99 |
| Scale4_ResponderRESP:TimepointFUP:TaskEO | -0.008 | -0.039 | 0.022 | 0.705 |
| Scale5_Intercept | 1.801 | 1.776 | 1.825 | 1 |
| Scale5_ResponderRESP | 0.018 | -0.007 | 0.044 | 0.923 |
| Scale5_TimepointPOST | 0.006 | -0.017 | 0.029 | 0.713 |
| Scale5_TimepointFUP | 0.004 | -0.021 | 0.028 | 0.611 |
| Scale5_TaskEO | -0.002 | -0.022 | 0.018 | 0.559 |
| Scale5_ResponderRESP:TimepointPOST | -0.019 | -0.046 | 0.008 | 0.921 |
| Scale5_ResponderRESP:TimepointFUP | -0.017 | -0.05 | 0.016 | 0.845 |
| Scale5_ResponderRESP:TaskEO | -0.007 | -0.029 | 0.016 | 0.713 |
| Scale5_TimepointPOST:TaskEO | 0.01 | -0.011 | 0.032 | 0.827 |
| Scale5_TimepointFUP:TaskEO | -0.005 | -0.026 | 0.017 | 0.664 |
| Scale5_ResponderRESP:TimepointPOST:TaskEO | -0.017 | -0.043 | 0.009 | 0.904 |
| Scale5_ResponderRESP:TimepointFUP:TaskEO | 0.004 | -0.025 | 0.032 | 0.602 |
| Scale6_Intercept | 1.815 | 1.79 | 1.838 | 1 |
| Scale6_ResponderRESP | 0.016 | -0.009 | 0.041 | 0.893 |
| Scale6_TimepointPOST | 0.014 | -0.01 | 0.039 | 0.875 |
| Scale6_TimepointFUP | 0.007 | -0.018 | 0.031 | 0.714 |
| Scale6_TaskEO | -0.022 | -0.043 | -0.002 | 0.982 |
| Scale6_ResponderRESP:TimepointPOST | -0.028 | -0.057 | 0 | 0.974 |
| Scale6_ResponderRESP:TimepointFUP | -0.021 | -0.055 | 0.012 | 0.9 |
| Scale6_ResponderRESP:TaskEO | -0.015 | -0.038 | 0.009 | 0.896 |
| Scale6_TimepointPOST:TaskEO | 0.004 | -0.019 | 0.028 | 0.651 |
| Scale6_TimepointFUP:TaskEO | -0.01 | -0.032 | 0.013 | 0.804 |
| Scale6_ResponderRESP:TimepointPOST:TaskEO | -0.006 | -0.034 | 0.022 | 0.671 |
| Scale6_ResponderRESP:TimepointFUP:TaskEO | 0.01 | -0.02 | 0.04 | 0.744 |
| Scale7_Intercept | 1.826 | 1.801 | 1.85 | 1 |
| Scale7_ResponderRESP | 0.012 | -0.014 | 0.038 | 0.819 |
| Scale7_TimepointPOST | 0.013 | -0.013 | 0.039 | 0.834 |
| Scale7_TimepointFUP | 0.007 | -0.018 | 0.033 | 0.706 |
| Scale7_TaskEO | -0.041 | -0.063 | -0.02 | 1 |
| Scale7_ResponderRESP:TimepointPOST | -0.028 | -0.058 | 0.003 | 0.962 |
| Scale7_ResponderRESP:TimepointFUP | -0.026 | -0.061 | 0.009 | 0.93 |
| Scale7_ResponderRESP:TaskEO | -0.018 | -0.043 | 0.007 | 0.923 |
| Scale7_TimepointPOST:TaskEO | 0.005 | -0.02 | 0.03 | 0.646 |
| Scale7_TimepointFUP:TaskEO | -0.008 | -0.032 | 0.016 | 0.748 |
| Scale7_ResponderRESP:TimepointPOST:TaskEO | -0.004 | -0.035 | 0.026 | 0.611 |
| Scale7_ResponderRESP:TimepointFUP:TaskEO | 0.011 | -0.022 | 0.042 | 0.74 |
| Scale8_Intercept | 1.825 | 1.798 | 1.852 | 1 |
| Scale8_ResponderRESP | 0.008 | -0.02 | 0.036 | 0.717 |
| Scale8_TimepointPOST | 0.016 | -0.013 | 0.044 | 0.861 |
| Scale8_TimepointFUP | 0.012 | -0.015 | 0.04 | 0.804 |
| Scale8_TaskEO | -0.052 | -0.076 | -0.028 | 1 |
| Scale8_ResponderRESP:TimepointPOST | -0.028 | -0.061 | 0.006 | 0.949 |
| Scale8_ResponderRESP:TimepointFUP | -0.029 | -0.066 | 0.009 | 0.933 |
| Scale8_ResponderRESP:TaskEO | -0.022 | -0.049 | 0.006 | 0.939 |
| Scale8_TimepointPOST:TaskEO | 0.003 | -0.025 | 0.03 | 0.585 |
| Scale8_TimepointFUP:TaskEO | -0.017 | -0.044 | 0.01 | 0.891 |
| Scale8_ResponderRESP:TimepointPOST:TaskEO | -0.001 | -0.034 | 0.033 | 0.522 |
| Scale8_ResponderRESP:TimepointFUP:TaskEO | 0.014 | -0.022 | 0.049 | 0.779 |
| Scale9_Intercept | 1.81 | 1.782 | 1.839 | 1 |
| Scale9_ResponderRESP | 0.013 | -0.018 | 0.042 | 0.798 |
| Scale9_TimepointPOST | 0.021 | -0.009 | 0.05 | 0.913 |
| Scale9_TimepointFUP | 0.02 | -0.009 | 0.049 | 0.91 |
| Scale9_TaskEO | -0.05 | -0.076 | -0.024 | 1 |
| Scale9_ResponderRESP:TimepointPOST | -0.03 | -0.065 | 0.006 | 0.953 |
| Scale9_ResponderRESP:TimepointFUP | -0.036 | -0.075 | 0.003 | 0.966 |
| Scale9_ResponderRESP:TaskEO | -0.036 | -0.066 | -0.006 | 0.99 |
| Scale9_TimepointPOST:TaskEO | -0.007 | -0.037 | 0.023 | 0.675 |
| Scale9_TimepointFUP:TaskEO | -0.027 | -0.056 | 0.002 | 0.967 |
| Scale9_ResponderRESP:TimepointPOST:TaskEO | 0.011 | -0.025 | 0.047 | 0.724 |
| Scale9_ResponderRESP:TimepointFUP:TaskEO | 0.027 | -0.012 | 0.065 | 0.915 |
| Scale10_Intercept | 1.796 | 1.764 | 1.828 | 1 |
| Scale10_ResponderRESP | 0.006 | -0.028 | 0.039 | 0.635 |
| Scale10_TimepointPOST | 0.023 | -0.009 | 0.054 | 0.92 |
| Scale10_TimepointFUP | 0.019 | -0.013 | 0.05 | 0.881 |
| Scale10_TaskEO | -0.055 | -0.083 | -0.026 | 1 |
| Scale10_ResponderRESP:TimepointPOST | -0.029 | -0.067 | 0.008 | 0.939 |
| Scale10_ResponderRESP:TimepointFUP | -0.035 | -0.077 | 0.008 | 0.946 |
| Scale10_ResponderRESP:TaskEO | -0.028 | -0.061 | 0.005 | 0.953 |
| Scale10_TimepointPOST:TaskEO | -0.008 | -0.041 | 0.026 | 0.668 |
| Scale10_TimepointFUP:TaskEO | -0.027 | -0.059 | 0.006 | 0.946 |
| Scale10_ResponderRESP:TimepointPOST:TaskEO | 0.01 | -0.031 | 0.05 | 0.68 |
| Scale10_ResponderRESP:TimepointFUP:TaskEO | 0.024 | -0.019 | 0.067 | 0.862 |

**Supplementary G. Frequentist Statistics**

Table S11. Frequentist statistics for Lempel-Ziv Complexity. Note: F-Ratio Statistic, F-Statistic.

| Effect | F-Statistic | P-value |
| --- | --- | --- |
| Responder | 1.791 | 0.1808 |
| Timepoint | 1.125 | 0.3247 |
| Task | 77.584 | <.001 |
| Responder:Timepoint | 0.077 | 0.926 |
| Responder:Task | 0.025 | 0.8735 |
| Timepoint:Task | 0.841 | 0.4313 |
| Responder:Timepoint:Task | 2.869 | 0.0568 |

Table S12. Frequentist statistics for Multi-Scale Entropy. Note: F-Ratio Statistic, F-Statistic; rep.meas, response measures (i.e. Scale 1, Scale 2, … Scale 10).

| Effect | F-Statistic | P-value |
| --- | --- | --- |
| Responder | 0.239 | 0.6248 |
| Timepoint | 0.131 | 0.8776 |
| Task | 5.484 | 0.0192 |
| rep.meas | 226.114 | <.001 |
| Responder:Timepoint | 6.716 | 0.0012 |
| Responder:Task | 7.909 | 0.0049 |
| Responder:rep.meas | 1.549 | 0.1243 |
| Timepoint:Task | 0.45 | 0.6378 |
| Timepoint:rep.meas | 0.44 | 0.9798 |
| Task:rep.meas | 43.551 | <.001 |
| Responder:Timepoint:Task | 6.441 | 0.0016 |
| Responder:Timepoint:rep.meas | 0.126 | 1 |
| Responder:Task:rep.meas | 0.231 | 0.9902 |
| Timepoint:Task:rep.meas | 0.347 | 0.9951 |
| Responder:Timepoint:Task:rep.meas | 1.218 | 0.2354 |

Table S13. Frequentist statistics for channel-level Lempel-Ziv Complexity. Note: F-Ratio Statistic, F-Statistic; rep.meas, response measures (i.e. Fp1, AF3, FC1, F3).

| Effect | F-Statistic | P-value |
| --- | --- | --- |
| Responder | 1.421 | 0.2333 |
| Timepoint | 2.018 | 0.1329 |
| Task | 106.046 | <.001 |
| rep.meas | 13.218 | <.001 |
| Responder:Timepoint | 8.702 | 0.0002 |
| Responder:Task | 0.756 | 0.3846 |
| Responder:rep.meas | 0.179 | 0.9107 |
| Timepoint:Task | 0.852 | 0.4265 |
| Timepoint:rep.meas | 0.579 | 0.7477 |
| Task:rep.meas | 8.179 | <.001 |
| Responder:Timepoint:Task | 14.304 | <.001 |
| Responder:Timepoint:rep.meas | 0.537 | 0.7804 |
| Responder:Task:rep.meas | 0.138 | 0.9373 |
| Timepoint:Task:rep.meas | 0.576 | 0.7496 |
| Responder:Timepoint:Task:rep.meas | 0.303 | 0.9354 |

**Supplementary H. Marginal Means**

Table S14. Estimated marginal means for Lempel-Ziv Complexity across timepoint, task, response status, and the interaction. Note: 95% Highest Posterior Density; 95% HPD.

| Task | Timepoint | Responder | Marginal Means | Lower (2.5%) HPD | Upper (97.5%) HPD |
| --- | --- | --- | --- | --- | --- |
| EC |  |  | 0.548 | 0.528 | 0.567 |
| EO |  |  | 0.606 | 0.584 | 0.627 |
|  | BAS |  | 0.576 | 0.555 | 0.596 |
|  | POST |  | 0.571 | 0.549 | 0.592 |
|  | FUP |  | 0.583 | 0.562 | 0.605 |
|  |  | NR | 0.569 | 0.546 | 0.593 |
|  |  | RESP | 0.584 | 0.563 | 0.604 |
| EC | BAS | NR | 0.549 | 0.519 | 0.577 |
|  |  | RESP | 0.553 | 0.53 | 0.575 |
|  | POST | NR | 0.531 | 0.499 | 0.564 |
|  |  | RESP | 0.551 | 0.527 | 0.575 |
|  | FUP | NR | 0.539 | 0.512 | 0.566 |
|  |  | RESP | 0.562 | 0.535 | 0.59 |
| EO | BAS | NR | 0.586 | 0.554 | 0.617 |
|  |  | RESP | 0.617 | 0.593 | 0.642 |
|  | POST | NR | 0.597 | 0.563 | 0.631 |
|  |  | RESP | 0.603 | 0.576 | 0.627 |
|  | FUP | NR | 0.613 | 0.583 | 0.642 |
|  |  | RESP | 0.618 | 0.589 | 0.647 |

Table S15. Estimated marginal means for Multi-Scale Entropy for each scale across timepoint, task, response status. Note: 95% Highest Posterior Density; 95% HPD.

| Response Measure | Task | Timepoint | Responder | Marginal Mean | Lower (2.5%) HPD | Upper (97.5%) HPD |
| --- | --- | --- | --- | --- | --- | --- |
| Scale1 | EC |  |  | 0.962 | 0.928 | 0.996 |
|  | EO |  |  | 1.087 | 1.05 | 1.126 |
| Scale2 | EC |  |  | 1.561 | 1.525 | 1.596 |
|  | EO |  |  | 1.695 | 1.655 | 1.735 |
| Scale3 | EC |  |  | 1.723 | 1.698 | 1.748 |
|  | EO |  |  | 1.8 | 1.773 | 1.828 |
| Scale4 | EC |  |  | 1.778 | 1.758 | 1.797 |
|  | EO |  |  | 1.811 | 1.79 | 1.831 |
| Scale5 | EC |  |  | 1.807 | 1.79 | 1.823 |
|  | EO |  |  | 1.802 | 1.783 | 1.82 |
| Scale6 | EC |  |  | 1.821 | 1.805 | 1.837 |
|  | EO |  |  | 1.79 | 1.773 | 1.808 |
| Scale7 | EC |  |  | 1.829 | 1.813 | 1.845 |
|  | EO |  |  | 1.778 | 1.76 | 1.796 |
| Scale8 | EC |  |  | 1.829 | 1.811 | 1.846 |
|  | EO |  |  | 1.764 | 1.744 | 1.783 |
| Scale9 | EC |  |  | 1.819 | 1.8 | 1.838 |
|  | EO |  |  | 1.746 | 1.726 | 1.767 |
| Scale10 | EC |  |  | 1.802 | 1.781 | 1.824 |
|  | EO |  |  | 1.728 | 1.704 | 1.75 |
| Scale1 |  | BAS |  | 1.022 | 0.986 | 1.057 |
|  |  | POST |  | 1.014 | 0.976 | 1.053 |
|  |  | FUP |  | 1.039 | 1.001 | 1.078 |
| Scale2 |  | BAS |  | 1.626 | 1.588 | 1.663 |
|  |  | POST |  | 1.617 | 1.577 | 1.658 |
|  |  | FUP |  | 1.64 | 1.598 | 1.68 |
| Scale3 |  | BAS |  | 1.763 | 1.738 | 1.79 |
|  |  | POST |  | 1.755 | 1.728 | 1.783 |
|  |  | FUP |  | 1.767 | 1.738 | 1.796 |
| Scale4 |  | BAS |  | 1.797 | 1.777 | 1.817 |
|  |  | POST |  | 1.793 | 1.77 | 1.813 |
|  |  | FUP |  | 1.794 | 1.772 | 1.817 |
| Scale5 |  | BAS |  | 1.807 | 1.789 | 1.824 |
|  |  | POST |  | 1.805 | 1.785 | 1.823 |
|  |  | FUP |  | 1.801 | 1.781 | 1.821 |
| Scale6 |  | BAS |  | 1.808 | 1.791 | 1.825 |
|  |  | POST |  | 1.808 | 1.789 | 1.827 |
|  |  | FUP |  | 1.802 | 1.782 | 1.821 |
| Scale7 |  | BAS |  | 1.806 | 1.789 | 1.823 |
|  |  | POST |  | 1.807 | 1.787 | 1.827 |
|  |  | FUP |  | 1.799 | 1.78 | 1.818 |
| Scale8 |  | BAS |  | 1.798 | 1.78 | 1.816 |
|  |  | POST |  | 1.801 | 1.779 | 1.822 |
|  |  | FUP |  | 1.791 | 1.77 | 1.811 |
| Scale9 |  | BAS |  | 1.783 | 1.763 | 1.802 |
|  |  | POST |  | 1.788 | 1.765 | 1.811 |
|  |  | FUP |  | 1.777 | 1.756 | 1.798 |
| Scale10 |  | BAS |  | 1.765 | 1.744 | 1.787 |
|  |  | POST |  | 1.771 | 1.746 | 1.797 |
|  |  | FUP |  | 1.759 | 1.734 | 1.782 |
| Scale1 |  |  | NR | 1.008 | 0.968 | 1.049 |
|  |  |  | RESP | 1.041 | 1.006 | 1.077 |
| Scale2 |  |  | NR | 1.608 | 1.565 | 1.651 |
|  |  |  | RESP | 1.648 | 1.609 | 1.685 |
| Scale3 |  |  | NR | 1.748 | 1.717 | 1.778 |
|  |  |  | RESP | 1.775 | 1.75 | 1.802 |
| Scale4 |  |  | NR | 1.788 | 1.765 | 1.812 |
|  |  |  | RESP | 1.801 | 1.78 | 1.821 |
| Scale5 |  |  | NR | 1.804 | 1.783 | 1.825 |
|  |  |  | RESP | 1.805 | 1.786 | 1.822 |
| Scale6 |  |  | NR | 1.81 | 1.79 | 1.83 |
|  |  |  | RESP | 1.802 | 1.785 | 1.82 |
| Scale7 |  |  | NR | 1.811 | 1.791 | 1.831 |
|  |  |  | RESP | 1.797 | 1.78 | 1.815 |
| Scale8 |  |  | NR | 1.806 | 1.784 | 1.828 |
|  |  |  | RESP | 1.787 | 1.767 | 1.805 |
| Scale9 |  |  | NR | 1.793 | 1.77 | 1.816 |
|  |  |  | RESP | 1.772 | 1.751 | 1.792 |
| Scale10 |  |  | NR | 1.777 | 1.752 | 1.804 |
|  |  |  | RESP | 1.753 | 1.731 | 1.776 |

Table S16. Estimated marginal means for Multi-Scale Entropy for each scale and the interaction of timepoint, task, and response status interaction. Note: 95% Highest Posterior Density; 95% HPD.

| Response Measure | Task | Timepoint | Responder | Marginal Mean | Lower (2.5%) HPD | Upper (97.5%) HPD |
| --- | --- | --- | --- | --- | --- | --- |
| Scale1 | EC | BAS | NR | 0.957 | 0.907 | 1.007 |
|  |  |  | RESP | 0.969 | 0.931 | 1.009 |
|  |  | POST | NR | 0.927 | 0.871 | 0.984 |
|  |  |  | RESP | 0.976 | 0.934 | 1.019 |
|  |  | FUP | NR | 0.949 | 0.902 | 0.999 |
|  |  |  | RESP | 0.995 | 0.947 | 1.044 |
|  | EO | BAS | NR | 1.053 | 0.999 | 1.107 |
|  |  |  | RESP | 1.108 | 1.066 | 1.153 |
|  |  | POST | NR | 1.065 | 1.002 | 1.124 |
|  |  |  | RESP | 1.087 | 1.042 | 1.133 |
|  |  | FUP | NR | 1.097 | 1.046 | 1.151 |
|  |  |  | RESP | 1.114 | 1.062 | 1.167 |
| Scale2 | EC | BAS | NR | 1.551 | 1.499 | 1.602 |
|  |  |  | RESP | 1.575 | 1.535 | 1.617 |
|  |  | POST | NR | 1.524 | 1.465 | 1.582 |
|  |  |  | RESP | 1.577 | 1.532 | 1.621 |
|  |  | FUP | NR | 1.545 | 1.494 | 1.595 |
|  |  |  | RESP | 1.594 | 1.542 | 1.646 |
|  | EO | BAS | NR | 1.656 | 1.597 | 1.712 |
|  |  |  | RESP | 1.724 | 1.677 | 1.767 |
|  |  | POST | NR | 1.675 | 1.612 | 1.737 |
|  |  |  | RESP | 1.693 | 1.645 | 1.74 |
|  |  | FUP | NR | 1.698 | 1.642 | 1.753 |
|  |  |  | RESP | 1.725 | 1.669 | 1.78 |
| Scale3 | EC | BAS | NR | 1.713 | 1.677 | 1.749 |
|  |  |  | RESP | 1.739 | 1.711 | 1.766 |
|  |  | POST | NR | 1.698 | 1.656 | 1.738 |
|  |  |  | RESP | 1.733 | 1.702 | 1.763 |
|  |  | FUP | NR | 1.711 | 1.676 | 1.747 |
|  |  |  | RESP | 1.742 | 1.706 | 1.779 |
|  | EO | BAS | NR | 1.775 | 1.734 | 1.813 |
|  |  |  | RESP | 1.825 | 1.794 | 1.855 |
|  |  | POST | NR | 1.793 | 1.75 | 1.836 |
|  |  |  | RESP | 1.796 | 1.763 | 1.826 |
|  |  | FUP | NR | 1.796 | 1.756 | 1.834 |
|  |  |  | RESP | 1.818 | 1.78 | 1.856 |
| Scale4 | EC | BAS | NR | 1.77 | 1.743 | 1.799 |
|  |  |  | RESP | 1.792 | 1.771 | 1.814 |
|  |  | POST | NR | 1.769 | 1.738 | 1.801 |
|  |  |  | RESP | 1.782 | 1.758 | 1.805 |
|  |  | FUP | NR | 1.771 | 1.744 | 1.799 |
|  |  |  | RESP | 1.784 | 1.756 | 1.812 |
|  | EO | BAS | NR | 1.798 | 1.769 | 1.829 |
|  |  |  | RESP | 1.827 | 1.804 | 1.851 |
|  |  | POST | NR | 1.816 | 1.783 | 1.848 |
|  |  |  | RESP | 1.802 | 1.778 | 1.826 |
|  |  | FUP | NR | 1.806 | 1.777 | 1.835 |
|  |  |  | RESP | 1.816 | 1.787 | 1.846 |
| Scale5 | EC | BAS | NR | 1.801 | 1.776 | 1.825 |
|  |  |  | RESP | 1.819 | 1.8 | 1.837 |
|  |  | POST | NR | 1.807 | 1.779 | 1.835 |
|  |  |  | RESP | 1.806 | 1.785 | 1.827 |
|  |  | FUP | NR | 1.804 | 1.779 | 1.828 |
|  |  |  | RESP | 1.805 | 1.782 | 1.831 |
|  | EO | BAS | NR | 1.799 | 1.773 | 1.825 |
|  |  |  | RESP | 1.811 | 1.79 | 1.83 |
|  |  | POST | NR | 1.816 | 1.786 | 1.845 |
|  |  |  | RESP | 1.791 | 1.769 | 1.813 |
|  |  | FUP | NR | 1.798 | 1.772 | 1.823 |
|  |  |  | RESP | 1.796 | 1.77 | 1.822 |
| Scale6 | EC | BAS | NR | 1.815 | 1.79 | 1.838 |
|  |  |  | RESP | 1.83 | 1.812 | 1.849 |
|  |  | POST | NR | 1.829 | 1.8 | 1.856 |
|  |  |  | RESP | 1.816 | 1.796 | 1.837 |
|  |  | FUP | NR | 1.822 | 1.797 | 1.844 |
|  |  |  | RESP | 1.816 | 1.792 | 1.84 |
|  | EO | BAS | NR | 1.793 | 1.767 | 1.819 |
|  |  |  | RESP | 1.793 | 1.774 | 1.814 |
|  |  | POST | NR | 1.811 | 1.782 | 1.841 |
|  |  |  | RESP | 1.777 | 1.755 | 1.799 |
|  |  | FUP | NR | 1.79 | 1.764 | 1.815 |
|  |  |  | RESP | 1.779 | 1.754 | 1.805 |
| Scale7 | EC | BAS | NR | 1.826 | 1.801 | 1.85 |
|  |  |  | RESP | 1.838 | 1.819 | 1.856 |
|  |  | POST | NR | 1.838 | 1.809 | 1.868 |
|  |  |  | RESP | 1.822 | 1.801 | 1.844 |
|  |  | FUP | NR | 1.833 | 1.809 | 1.857 |
|  |  |  | RESP | 1.818 | 1.794 | 1.844 |
|  | EO | BAS | NR | 1.784 | 1.758 | 1.81 |
|  |  |  | RESP | 1.778 | 1.757 | 1.798 |
|  |  | POST | NR | 1.802 | 1.771 | 1.833 |
|  |  |  | RESP | 1.763 | 1.741 | 1.787 |
|  |  | FUP | NR | 1.783 | 1.758 | 1.809 |
|  |  |  | RESP | 1.761 | 1.735 | 1.787 |
| Scale8 | EC | BAS | NR | 1.825 | 1.799 | 1.852 |
|  |  |  | RESP | 1.833 | 1.813 | 1.853 |
|  |  | POST | NR | 1.84 | 1.809 | 1.873 |
|  |  |  | RESP | 1.821 | 1.797 | 1.844 |
|  |  | FUP | NR | 1.837 | 1.812 | 1.863 |
|  |  |  | RESP | 1.816 | 1.79 | 1.843 |
|  | EO | BAS | NR | 1.773 | 1.744 | 1.802 |
|  |  |  | RESP | 1.76 | 1.737 | 1.781 |
|  |  | POST | NR | 1.792 | 1.757 | 1.825 |
|  |  |  | RESP | 1.749 | 1.724 | 1.774 |
|  |  | FUP | NR | 1.769 | 1.741 | 1.796 |
|  |  |  | RESP | 1.74 | 1.713 | 1.768 |
| Scale9 | EC | BAS | NR | 1.81 | 1.783 | 1.839 |
|  |  |  | RESP | 1.823 | 1.802 | 1.844 |
|  |  | POST | NR | 1.831 | 1.797 | 1.864 |
|  |  |  | RESP | 1.814 | 1.788 | 1.839 |
|  |  | FUP | NR | 1.83 | 1.803 | 1.857 |
|  |  |  | RESP | 1.806 | 1.778 | 1.834 |
|  | EO | BAS | NR | 1.76 | 1.729 | 1.79 |
|  |  |  | RESP | 1.737 | 1.714 | 1.761 |
|  |  | POST | NR | 1.774 | 1.737 | 1.81 |
|  |  |  | RESP | 1.732 | 1.705 | 1.759 |
|  |  | FUP | NR | 1.753 | 1.724 | 1.782 |
|  |  |  | RESP | 1.72 | 1.689 | 1.748 |
| Scale10 | EC | BAS | NR | 1.796 | 1.764 | 1.828 |
|  |  |  | RESP | 1.802 | 1.777 | 1.826 |
|  |  | POST | NR | 1.819 | 1.781 | 1.855 |
|  |  |  | RESP | 1.795 | 1.767 | 1.823 |
|  |  | FUP | NR | 1.815 | 1.785 | 1.846 |
|  |  |  | RESP | 1.786 | 1.754 | 1.816 |
|  | EO | BAS | NR | 1.741 | 1.708 | 1.775 |
|  |  |  | RESP | 1.719 | 1.693 | 1.746 |
|  |  | POST | NR | 1.757 | 1.717 | 1.796 |
|  |  |  | RESP | 1.715 | 1.685 | 1.743 |
|  |  | FUP | NR | 1.734 | 1.702 | 1.766 |
|  |  |  | RESP | 1.701 | 1.669 | 1.732 |

Table S17. Estimated marginal means Lempel-Ziv Complexity at each channel across timepoint, task, response status, and their interaction. Note: 95% Highest Posterior Density; 95% HPD.

| Response Measure | Task | Timepoint | Responder | Marginal Mean | Lower (2.5%) HPD | Upper (97.5%) HPD |
| --- | --- | --- | --- | --- | --- | --- |
| Fp1 | EC |  |  | 0.551 | 0.527 | 0.574 |
|  | EO |  |  | 0.645 | 0.616 | 0.675 |
| AF3 | EC |  |  | 0.552 | 0.528 | 0.577 |
|  | EO |  |  | 0.644 | 0.613 | 0.673 |
| FC1 | EC |  |  | 0.529 | 0.511 | 0.547 |
|  | EO |  |  | 0.566 | 0.547 | 0.586 |
| F3 | EC |  |  | 0.575 | 0.552 | 0.598 |
|  | EO |  |  | 0.654 | 0.628 | 0.679 |
| Fp1 |  | BAS |  | 0.589 | 0.564 | 0.615 |
|  |  | POST |  | 0.593 | 0.565 | 0.62 |
| Fp1 |  | FUP |  | 0.612 | 0.581 | 0.643 |
|  |  | BAS |  | 0.589 | 0.562 | 0.615 |
| AF3 |  | POST |  | 0.593 | 0.565 | 0.62 |
|  |  | FUP |  | 0.611 | 0.581 | 0.64 |
| FC1 |  | BAS |  | 0.547 | 0.529 | 0.563 |
|  |  | POST |  | 0.551 | 0.527 | 0.574 |
| FC1 |  | FUP |  | 0.545 | 0.52 | 0.569 |
|  |  | BAS |  | 0.61 | 0.585 | 0.635 |
| F3 |  | POST |  | 0.605 | 0.58 | 0.632 |
|  |  | FUP |  | 0.628 | 0.598 | 0.657 |
| Fp1 |  |  | NR | 0.587 | 0.549 | 0.624 |
|  |  |  | RESP | 0.609 | 0.583 | 0.634 |
| AF3 |  |  | NR | 0.583 | 0.542 | 0.621 |
|  |  |  | RESP | 0.612 | 0.585 | 0.638 |
| FC1 |  |  | NR | 0.54 | 0.511 | 0.568 |
|  |  |  | RESP | 0.555 | 0.535 | 0.575 |
| F3 |  |  | NR | 0.608 | 0.572 | 0.643 |
|  |  |  | RESP | 0.621 | 0.597 | 0.645 |
|  |  |  |  |  |  |  |
| Fp1 | EC | BAS | NR | 0.537 | 0.493 | 0.582 |
| AF3 |  |  |  | 0.531 | 0.484 | 0.577 |
| FC1 |  |  |  | 0.525 | 0.495 | 0.555 |
| F3 |  |  |  | 0.571 | 0.526 | 0.614 |
| Fp1 |  |  | RESP | 0.554 | 0.523 | 0.585 |
| AF3 |  |  |  | 0.559 | 0.526 | 0.59 |
| FC1 |  |  |  | 0.537 | 0.515 | 0.558 |
| F3 |  |  |  | 0.58 | 0.549 | 0.611 |
| Fp1 | EO |  | NR | 0.591 | 0.537 | 0.646 |
| AF3 |  |  |  | 0.582 | 0.528 | 0.638 |
| FC1 |  |  |  | 0.533 | 0.5 | 0.566 |
| F3 |  |  |  | 0.61 | 0.562 | 0.661 |
| Fp1 |  |  | RESP | 0.675 | 0.637 | 0.714 |
| AF3 |  |  |  | 0.683 | 0.646 | 0.721 |
| FC1 |  |  |  | 0.592 | 0.567 | 0.615 |
| F3 |  |  |  | 0.68 | 0.645 | 0.714 |
| Fp1 | EC | POST | NR | 0.544 | 0.494 | 0.593 |
| AF3 |  |  |  | 0.545 | 0.494 | 0.593 |
| FC1 |  |  |  | 0.525 | 0.484 | 0.566 |
| F3 |  |  |  | 0.548 | 0.502 | 0.597 |
| Fp1 |  |  | RESP | 0.556 | 0.522 | 0.59 |
| AF3 |  |  |  | 0.559 | 0.525 | 0.592 |
| FC1 |  |  |  | 0.531 | 0.502 | 0.56 |
| F3 |  |  |  | 0.577 | 0.545 | 0.61 |
| Fp1 | EO |  | NR | 0.629 | 0.572 | 0.688 |
| AF3 |  |  |  | 0.632 | 0.576 | 0.69 |
| FC1 |  |  |  | 0.573 | 0.53 | 0.615 |
| F3 |  |  |  | 0.64 | 0.585 | 0.69 |
| Fp1 |  |  | RESP | 0.643 | 0.603 | 0.681 |
| AF3 |  |  |  | 0.636 | 0.597 | 0.673 |
| FC1 |  |  |  | 0.576 | 0.546 | 0.604 |
| F3 |  |  |  | 0.657 | 0.621 | 0.691 |
| Fp1 | EC | FUP | NR | 0.557 | 0.503 | 0.611 |
| AF3 |  |  |  | 0.546 | 0.493 | 0.597 |
| FC1 |  |  |  | 0.523 | 0.479 | 0.564 |
| F3 |  |  |  | 0.589 | 0.535 | 0.641 |
| Fp1 |  |  | RESP | 0.558 | 0.52 | 0.595 |
| AF3 |  |  |  | 0.572 | 0.537 | 0.609 |
| FC1 |  |  |  | 0.534 | 0.504 | 0.563 |
| F3 |  |  |  | 0.588 | 0.551 | 0.625 |
| Fp1 | EO |  | NR | 0.666 | 0.603 | 0.728 |
| AF3 |  |  |  | 0.663 | 0.604 | 0.725 |
| FC1 |  |  |  | 0.561 | 0.516 | 0.608 |
| F3 |  |  |  | 0.689 | 0.631 | 0.747 |
| Fp1 |  |  | RESP | 0.666 | 0.621 | 0.706 |
| AF3 |  |  |  | 0.665 | 0.624 | 0.706 |
| FC1 |  |  |  | 0.563 | 0.532 | 0.596 |
| F3 |  |  |  | 0.645 | 0.604 | 0.683 |

**Supplementary I. JASP Output**

Table S18. Paired Samples T-Test of baseline Lempel-Ziv Complexity by task, and channel. Note: Eyes Closed, EC; Eyes Open, EC; Bayes Factor, BF_10_; Credible Intervals, CIs; Standard Deviation, SD; Sample size, N.

| Channel | Task | N | Mean (SD) | 95% CIs [Lower; Upper] | BF₁₀ (error %) |
| --- | --- | --- | --- | --- | --- |
| Fp1 | EC | 30 | 0.557 (0.084) | [0.525; 0.588] | 742.923 (9.437×10-9) |
|  | EO | 28 | 0.657 (0.117) | [0.611; 0.702] |  |
| AF3 | EC | 30 | 0.56 (0.096) | [0.524; 0.596] | 1152.64 (6.123×10-9) |
|  | EO | 28 | 0.66 (0.125) | [0.611; 0.708] |  |
| F7 | EC | 30 | 0.584 (0.097) | [0.548; 0.62] | 104.058 (1.386×10-4) |
|  | EO | 28 | 0.664 (0.086) | [0.63; 0.697] |  |
| F3 | EC | 30 | 0.586 (0.088) | [0.554; 0.619] | 302.039 (1.003×10-4) |
|  | EO | 28 | 0.665 (0.099) | [0.626; 0.703] |  |
| FC1 | EC | 30 | 0.537 (0.055) | [0.516; 0.558] | 11.531 (4.152×10-8) |
|  | EO | 28 | 0.578 (0.068) | [0.551; 0.604] |  |
| FC5 | EC | 30 | 0.601 (0.106) | [0.562; 0.641] | 96.982 (1.792×10-4) |
|  | EO | 28 | 0.685 (0.1) | [0.646; 0.723] |  |
| T7 | EC | 30 | 0.577 (0.09) | [0.543; 0.61] | 2501.139 (3.778×10-9) |
|  | EO | 28 | 0.671 (0.093) | [0.635; 0.707] |  |
| C3 | EC | 30 | 0.566 (0.09) | [0.533; 0.6] | 3.525 (6.134×10-7) |
|  | EO | 28 | 0.606 (0.084) | [0.573; 0.638] |  |
| CP1 | EC | 30 | 0.512 (0.06) | [0.49; 0.535] | 6.023 (2.240×10-7) |
|  | EO | 28 | 0.535 (0.056) | [0.513; 0.556] |  |
| CP5 | EC | 30 | 0.569 (0.089) | [0.536; 0.603] | 7.533 (1.342×10-7) |
|  | EO | 28 | 0.62 (0.084) | [0.588; 0.653] |  |
| P7 | EC | 30 | 0.546 (0.082) | [0.516; 0.577] | 7502.38 (2.524×10-9) |
|  | EO | 28 | 0.633 (0.081) | [0.602; 0.665] |  |
| P3 | EC | 30 | 0.504 (0.065) | [0.48; 0.528] | 6.824 (1.699×10-7) |
|  | EO | 28 | 0.548 (0.067) | [0.522; 0.574] |  |
| Pz | EC | 30 | 0.488 (0.057) | [0.467; 0.509] | 4.481 (4.031×10-7) |
|  | EO | 28 | 0.514 (0.038) | [0.499; 0.528] |  |
| PO3 | EC | 30 | 0.501 (0.059) | [0.479; 0.523] | 120.536 (7.086×10-5) |
|  | EO | 28 | 0.544 (0.06) | [0.521; 0.567] |  |
| O1 | EC | 30 | 0.545 (0.09) | [0.512; 0.579] | 229.426 (4.968×10-5) |
|  | EO | 28 | 0.608 (0.084) | [0.575; 0.64] |  |
| Oz | EC | 30 | 0.544 (0.089) | [0.511; 0.577] | 528.419 (1.351×10-8) |
|  | EO | 28 | 0.594 (0.084) | [0.562; 0.627] |  |
| O2 | EC | 30 | 0.555 (0.095) | [0.519; 0.591] | 66.789 (4.963×10-4) |
|  | EO | 28 | 0.607 (0.082) | [0.575; 0.639] |  |
| PO4 | EC | 30 | 0.512 (0.081) | [0.482; 0.542] | 7.316 (1.442×10-7) |
|  | EO | 28 | 0.554 (0.056) | [0.533; 0.576] |  |
| P4 | EC | 30 | 0.51 (0.078) | [0.48; 0.539] | 2.499 (1.049×10-6) |
|  | EO | 28 | 0.539 (0.052) | [0.519; 0.559] |  |
| P8 | EC | 30 | 0.553 (0.087) | [0.52; 0.585] | 312.754 (1.062×10-4) |
|  | EO | 28 | 0.623 (0.083) | [0.591; 0.655] |  |
| CP6 | EC | 30 | 0.588 (0.085) | [0.556; 0.619] | 3.566 (6.016×10-7) |
|  | EO | 28 | 0.623 (0.085) | [0.59; 0.656] |  |
| CP2 | EC | 30 | 0.528 (0.069) | [0.502; 0.554] | 0.528 (0.028) |
|  | EO | 28 | 0.546 (0.05) | [0.526; 0.565] |  |
| C4 | EC | 30 | 0.567 (0.065) | [0.542; 0.591] | 33.742 (4.280×10-8) |
|  | EO | 28 | 0.611 (0.072) | [0.583; 0.639] |  |
| T8 | EC | 30 | 0.595 (0.102) | [0.557; 0.634] | 2.776 (8.969×10-7) |
|  | EO | 28 | 0.639 (0.085) | [0.606; 0.672] |  |
| FC6 | EC | 30 | 0.589 (0.097) | [0.552; 0.625] | 1298.428 (5.529×10-9) |
|  | EO | 28 | 0.668 (0.097) | [0.63; 0.705] |  |
| FC2 | EC | 30 | 0.536 (0.058) | [0.514; 0.557] | 3.745 (5.540×10-7) |
|  | EO | 28 | 0.561 (0.061) | [0.538; 0.585] |  |
| F4 | EC | 30 | 0.593 (0.079) | [0.564; 0.623] | 4519.35 (3.524×10-9) |
|  | EO | 28 | 0.667 (0.085) | [0.634; 0.7] |  |
| F8 | EC | 30 | 0.599 (0.107) | [0.559; 0.639] | 32.898 (4.201×10-8) |
|  | EO | 28 | 0.664 (0.084) | [0.631; 0.696] |  |
| AF4 | EC | 30 | 0.572 (0.089) | [0.539; 0.605] | 605.027 (1.171×10-8) |
|  | EO | 28 | 0.651 (0.106) | [0.61; 0.692] |  |
| Fp2 | EC | 30 | 0.568 (0.093) | [0.534; 0.603] | 578.716 (1.228×10-8) |
|  | EO | 28 | 0.656 (0.097) | [0.618; 0.694] |  |
| Fz | EC | 30 | 0.526 (0.067) | [0.501; 0.551] | 6.021 (2.242×10-7) |
|  | EO | 28 | 0.546 (0.065) | [0.521; 0.571] |  |
| Cz | EC | 30 | 0.533 (0.047) | [0.515; 0.551] | 0.847 (0.025) |
|  | EO | 28 | 0.548 (0.037) | [0.533; 0.562] |  |

Table S19. Independent Samples T-test of Baseline Lempel-Ziv Complexity by Response Status, Task, and Channel. Note: Eyes Closed, EC; Eyes Open, EC; Bayes Factor, BF_10_; Credible Intervals, CIs; Non-Responder, NR; Responder, RESP; Standard Deviation, SD; Sample Size, N.

| Channel | Task | Group | N | Mean (SD) | 95% CIs [Lower; Upper] | BF₁₀ (error %) |
| --- | --- | --- | --- | --- | --- | --- |
| Fp1 | EC | NR | 9 | 0.549 (0.096) | [0.475; 0.623] | 0.383 (0.002) |
|  |  | RESP | 21 | 0.56 (0.081) | [0.524; 0.597] |  |
|  | EO | NR | 9 | 0.595 (0.142) | [0.486; 0.704] | 1.581 (0.004) |
|  |  | RESP | 19 | 0.686 (0.094) | [0.641; 0.732] |  |
| AF3 | EC | NR | 9 | 0.546 (0.092) | [0.476; 0.617] | 0.403 (0.002) |
|  |  | RESP | 21 | 0.566 (0.1) | [0.52; 0.611] |  |
|  | EO | NR | 9 | 0.583 (0.151) | [0.467; 0.699] | 2.812 (0.006) |
|  |  | RESP | 19 | 0.696 (0.094) | [0.65; 0.741] |  |
| F7 | EC | NR | 9 | 0.582 (0.114) | [0.494; 0.669] | 0.368 (0.002) |
|  |  | RESP | 21 | 0.585 (0.092) | [0.543; 0.626] |  |
|  | EO | NR | 9 | 0.629 (0.066) | [0.578; 0.679] | 0.844 (0.003) |
|  |  | RESP | 19 | 0.68 (0.091) | [0.636; 0.724] |  |
| F3 | EC | NR | 9 | 0.588 (0.084) | [0.524; 0.653] | 0.368 (0.002) |
|  |  | RESP | 21 | 0.586 (0.092) | [0.544; 0.627] |  |
|  | EO | NR | 9 | 0.609 (0.115) | [0.52; 0.697] | 1.998 (0.004) |
|  |  | RESP | 19 | 0.691 (0.081) | [0.652; 0.73] |  |
| FC1 | EC | NR | 9 | 0.531 (0.054) | [0.489; 0.572] | 0.392 (0.002) |
|  |  | RESP | 21 | 0.54 (0.057) | [0.514; 0.566] |  |
|  | EO | NR | 9 | 0.535 (0.079) | [0.474; 0.596] | 3.34 (0.008) |
|  |  | RESP | 19 | 0.598 (0.053) | [0.573; 0.624] |  |
| FC5 | EC | NR | 9 | 0.602 (0.138) | [0.496; 0.708] | 0.367 (0.002) |
|  |  | RESP | 21 | 0.601 (0.093) | [0.559; 0.643] |  |
|  | EO | NR | 9 | 0.654 (0.101) | [0.577; 0.732] | 0.573 (0.002) |
|  |  | RESP | 19 | 0.699 (0.1) | [0.651; 0.747] |  |
| T7 | EC | NR | 9 | 0.594 (0.113) | [0.507; 0.681] | 0.437 (0.002) |
|  |  | RESP | 21 | 0.569 (0.079) | [0.533; 0.605] |  |
|  | EO | NR | 9 | 0.648 (0.107) | [0.566; 0.731] | 0.496 (0.002) |
|  |  | RESP | 19 | 0.682 (0.086) | [0.64; 0.724] |  |
| C3 | EC | NR | 9 | 0.576 (0.132) | [0.474; 0.677] | 0.384 (0.002) |
|  |  | RESP | 21 | 0.563 (0.068) | [0.531; 0.594] |  |
|  | EO | NR | 9 | 0.572 (0.106) | [0.49; 0.653] | 0.84 (0.003) |
|  |  | RESP | 19 | 0.622 (0.068) | [0.589; 0.655] |  |
| CP1 | EC | NR | 9 | 0.509 (0.075) | [0.451; 0.566] | 0.372 (0.002) |
|  |  | RESP | 21 | 0.513 (0.055) | [0.488; 0.539] |  |
|  | EO | NR | 9 | 0.525 (0.067) | [0.473; 0.577] | 0.427 (0.002) |
|  |  | RESP | 19 | 0.539 (0.051) | [0.515; 0.564] |  |
| CP5 | EC | NR | 9 | 0.574 (0.12) | [0.482; 0.666] | 0.371 (0.002) |
|  |  | RESP | 21 | 0.567 (0.077) | [0.533; 0.602] |  |
|  | EO | NR | 9 | 0.607 (0.103) | [0.528; 0.686] | 0.42 (0.002) |
|  |  | RESP | 19 | 0.627 (0.076) | [0.59; 0.663] |  |
| P7 | EC | NR | 9 | 0.561 (0.106) | [0.48; 0.642] | 0.424 (0.002) |
|  |  | RESP | 21 | 0.54 (0.071) | [0.508; 0.573] |  |
|  | EO | NR | 9 | 0.642 (0.09) | [0.573; 0.711] | 0.392 (0.002) |
|  |  | RESP | 19 | 0.629 (0.079) | [0.591; 0.667] |  |
| P3 | EC | NR | 9 | 0.511 (0.085) | [0.446; 0.577] | 0.39 (0.002) |
|  |  | RESP | 21 | 0.5 (0.056) | [0.475; 0.526] |  |
|  | EO | NR | 9 | 0.535 (0.072) | [0.48; 0.591] | 0.436 (0.002) |
|  |  | RESP | 19 | 0.554 (0.066) | [0.522; 0.586] |  |
| Pz | EC | NR | 9 | 0.505 (0.079) | [0.444; 0.566] | 0.574 (0.002) |
|  |  | RESP | 21 | 0.48 (0.044) | [0.46; 0.501] |  |
|  | EO | NR | 9 | 0.513 (0.049) | [0.475; 0.551] | 0.371 (0.002) |
|  |  | RESP | 19 | 0.514 (0.033) | [0.498; 0.53] |  |
| PO3 | EC | NR | 9 | 0.528 (0.093) | [0.457; 0.6] | 1.067 (0.003) |
|  |  | RESP | 21 | 0.489 (0.032) | [0.474; 0.504] |  |
|  | EO | NR | 9 | 0.561 (0.061) | [0.514; 0.608] | 0.554 (0.002) |
|  |  | RESP | 19 | 0.536 (0.059) | [0.507; 0.564] |  |
| O1 | EC | NR | 9 | 0.584 (0.1) | [0.507; 0.661] | 0.902 (0.003) |
|  |  | RESP | 21 | 0.529 (0.082) | [0.491; 0.566] |  |
|  | EO | NR | 9 | 0.627 (0.092) | [0.557; 0.698] | 0.486 (0.002) |
|  |  | RESP | 19 | 0.598 (0.08) | [0.559; 0.637] |  |
| Oz | EC | NR | 9 | 0.571 (0.099) | [0.495; 0.647] | 0.559 (0.002) |
|  |  | RESP | 21 | 0.533 (0.085) | [0.494; 0.571] |  |
|  | EO | NR | 9 | 0.627 (0.085) | [0.561; 0.692] | 0.767 (0.003) |
|  |  | RESP | 19 | 0.579 (0.081) | [0.54; 0.618] |  |
| O2 | EC | NR | 9 | 0.574 (0.111) | [0.489; 0.659] | 0.445 (0.002) |
|  |  | RESP | 21 | 0.547 (0.09) | [0.506; 0.587] |  |
|  | EO | NR | 9 | 0.642 (0.085) | [0.576; 0.707] | 0.893 (0.003) |
|  |  | RESP | 19 | 0.591 (0.078) | [0.553; 0.628] |  |
| PO4 | EC | NR | 9 | 0.521 (0.109) | [0.437; 0.605] | 0.388 (0.002) |
|  |  | RESP | 21 | 0.508 (0.069) | [0.477; 0.539] |  |
|  | EO | NR | 9 | 0.561 (0.076) | [0.502; 0.619] | 0.395 (0.002) |
|  |  | RESP | 19 | 0.551 (0.046) | [0.529; 0.573] |  |
| P4 | EC | NR | 9 | 0.517 (0.089) | [0.448; 0.586] | 0.382 (0.002) |
|  |  | RESP | 21 | 0.507 (0.075) | [0.472; 0.541] |  |
|  | EO | NR | 9 | 0.545 (0.067) | [0.493; 0.596] | 0.392 (0.002) |
|  |  | RESP | 19 | 0.537 (0.045) | [0.515; 0.558] |  |
| P8 | EC | NR | 9 | 0.579 (0.107) | [0.497; 0.661] | 0.565 (0.002) |
|  |  | RESP | 21 | 0.541 (0.077) | [0.506; 0.576] |  |
|  | EO | NR | 9 | 0.628 (0.097) | [0.553; 0.703] | 0.378 (0.002) |
|  |  | RESP | 19 | 0.62 (0.079) | [0.582; 0.658] |  |
| CP6 | EC | NR | 9 | 0.608 (0.104) | [0.528; 0.688] | 0.483 (0.002) |
|  |  | RESP | 21 | 0.579 (0.076) | [0.544; 0.613] |  |
|  | EO | NR | 9 | 0.631 (0.1) | [0.554; 0.708] | 0.387 (0.002) |
|  |  | RESP | 19 | 0.619 (0.08) | [0.581; 0.658] |  |
| CP2 | EC | NR | 9 | 0.523 (0.057) | [0.479; 0.566] | 0.376 (0.002) |
|  |  | RESP | 21 | 0.53 (0.076) | [0.495; 0.564] |  |
|  | EO | NR | 9 | 0.534 (0.062) | [0.487; 0.582] | 0.47 (0.002) |
|  |  | RESP | 19 | 0.551 (0.045) | [0.529; 0.573] |  |
| C4 | EC | NR | 9 | 0.561 (0.074) | [0.504; 0.617] | 0.381 (0.002) |
|  |  | RESP | 21 | 0.569 (0.063) | [0.541; 0.598] |  |
|  | EO | NR | 9 | 0.597 (0.072) | [0.541; 0.652] | 0.453 (0.002) |
|  |  | RESP | 19 | 0.618 (0.073) | [0.583; 0.654] |  |
| T8 | EC | NR | 9 | 0.608 (0.131) | [0.507; 0.708] | 0.393 (0.002) |
|  |  | RESP | 21 | 0.59 (0.09) | [0.549; 0.631] |  |
|  | EO | NR | 9 | 0.631 (0.099) | [0.555; 0.708] | 0.385 (0.002) |
|  |  | RESP | 19 | 0.643 (0.081) | [0.604; 0.682] |  |
| FC6 | EC | NR | 9 | 0.597 (0.103) | [0.518; 0.677] | 0.381 (0.002) |
|  |  | RESP | 21 | 0.585 (0.097) | [0.54; 0.629] |  |
|  | EO | NR | 9 | 0.652 (0.108) | [0.569; 0.735] | 0.419 (0.002) |
|  |  | RESP | 19 | 0.675 (0.093) | [0.63; 0.72] |  |
| FC2 | EC | NR | 9 | 0.539 (0.035) | [0.512; 0.565] | 0.372 (0.002) |
|  |  | RESP | 21 | 0.534 (0.067) | [0.504; 0.565] |  |
|  | EO | NR | 9 | 0.553 (0.061) | [0.506; 0.599] | 0.407 (0.002) |
|  |  | RESP | 19 | 0.565 (0.063) | [0.535; 0.596] |  |
| F4 | EC | NR | 9 | 0.575 (0.07) | [0.521; 0.628] | 0.471 (0.002) |
|  |  | RESP | 21 | 0.601 (0.083) | [0.563; 0.639] |  |
|  | EO | NR | 9 | 0.642 (0.111) | [0.556; 0.727] | 0.562 (0.002) |
|  |  | RESP | 19 | 0.679 (0.07) | [0.645; 0.713] |  |
| F8 | EC | NR | 9 | 0.606 (0.112) | [0.52; 0.692] | 0.374 (0.002) |
|  |  | RESP | 21 | 0.596 (0.107) | [0.547; 0.645] |  |
|  | EO | NR | 9 | 0.634 (0.077) | [0.575; 0.694] | 0.677 (0.002) |
|  |  | RESP | 19 | 0.678 (0.086) | [0.636; 0.719] |  |
| AF4 | EC | NR | 9 | 0.557 (0.09) | [0.488; 0.626] | 0.419 (0.002) |
|  |  | RESP | 21 | 0.578 (0.09) | [0.537; 0.62] |  |
|  | EO | NR | 9 | 0.615 (0.132) | [0.514; 0.716] | 0.66 (0.002) |
|  |  | RESP | 19 | 0.669 (0.09) | [0.625; 0.712] |  |
| Fp2 | EC | NR | 9 | 0.565 (0.108) | [0.482; 0.649] | 0.369 (0.002) |
|  |  | RESP | 21 | 0.57 (0.089) | [0.529; 0.61] |  |
|  | EO | NR | 9 | 0.628 (0.122) | [0.534; 0.722] | 0.556 (0.002) |
|  |  | RESP | 19 | 0.669 (0.083) | [0.629; 0.709] |  |
| Fz | EC | NR | 9 | 0.533 (0.063) | [0.484; 0.581] | 0.383 (0.002) |
|  |  | RESP | 21 | 0.523 (0.07) | [0.492; 0.555] |  |
|  | EO | NR | 9 | 0.535 (0.07) | [0.481; 0.589] | 0.421 (0.002) |
|  |  | RESP | 19 | 0.551 (0.063) | [0.52; 0.581] |  |
| Cz | EC | NR | 9 | 0.54 (0.054) | [0.498; 0.581] | 0.401 (0.002) |
|  |  | RESP | 21 | 0.53 (0.045) | [0.51; 0.551] |  |
|  | EO | NR | 9 | 0.54 (0.044) | [0.506; 0.573] | 0.456 (0.002) |
|  |  | RESP | 19 | 0.551 (0.035) | [0.535; 0.568] |  |

**Supplementary J. Time of Day**

Figure S20a. Estimated marginal means for Lempel-Ziv Complexity across recording time and task by response status. Note: Bars represent 95% Highest Posterior Density; Baseline, BAS; post-treatment, POST; follow-up, FUP; Before 12pm, Pre12; After 12pm, Post12; Eyes Closed, EC; Eyes Open, EO; NR, Non-Responder; RESP, Responder.


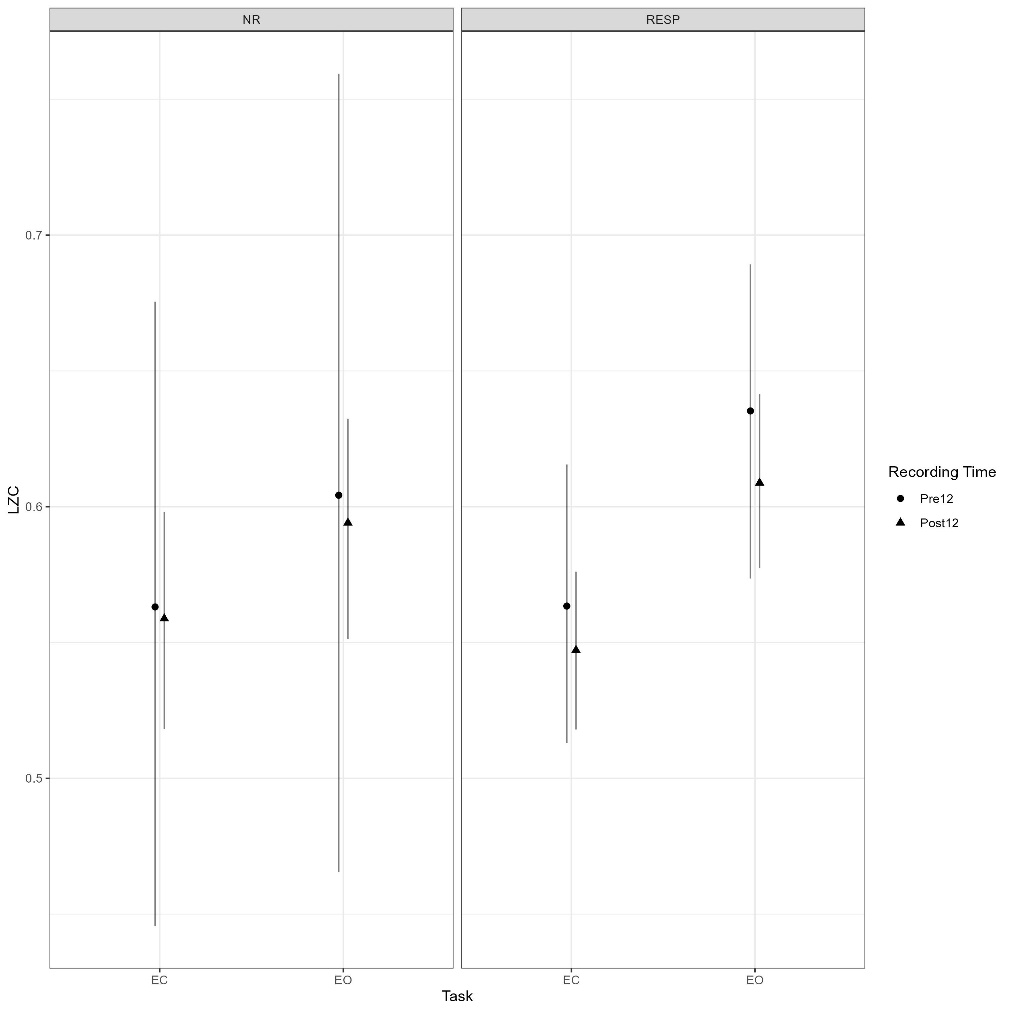


Figure S20b. Estimated marginal means for Lempel-Ziv Complexity across recording time and response status by task. Note: Bars represent 95% Highest Posterior Density; Baseline, BAS; post-treatment, POST; follow-up, FUP; Before 12pm, Pre12; After 12pm, Post12; Eyes Closed, EC; Eyes Open, EO; NR, Non-Responder; RESP, Responder.


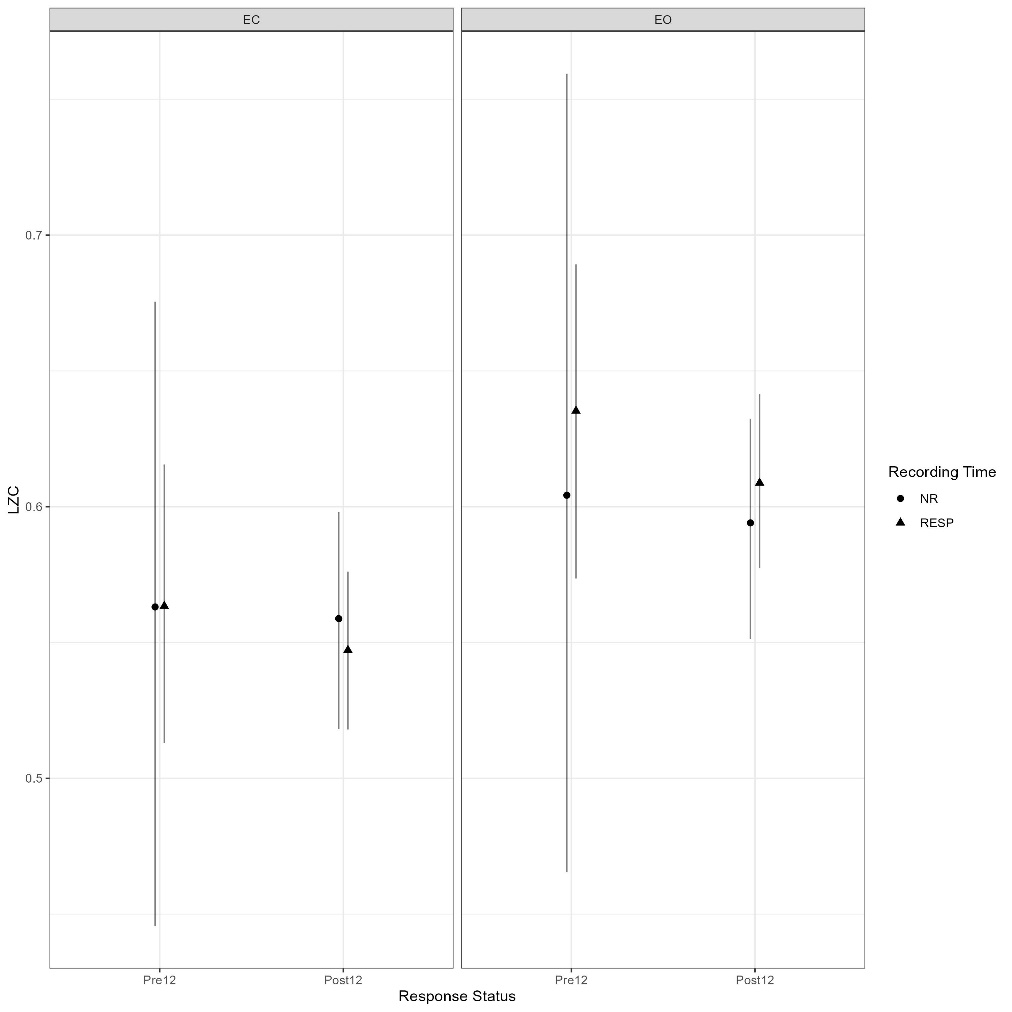


Figure S21a. Estimated marginal means for Multi-Scale Entropy across recording time and scale by task and response status. Note: Bars represent 95% Highest Posterior Density; Baseline, BAS; post-treatment, POST; follow-up, FUP; Before 12pm, Pre12; After 12pm, Post12; Eyes Closed, EC; Eyes Open, EO; NR, Non-Responder; RESP, Responder; Response Measures, resp.meas.


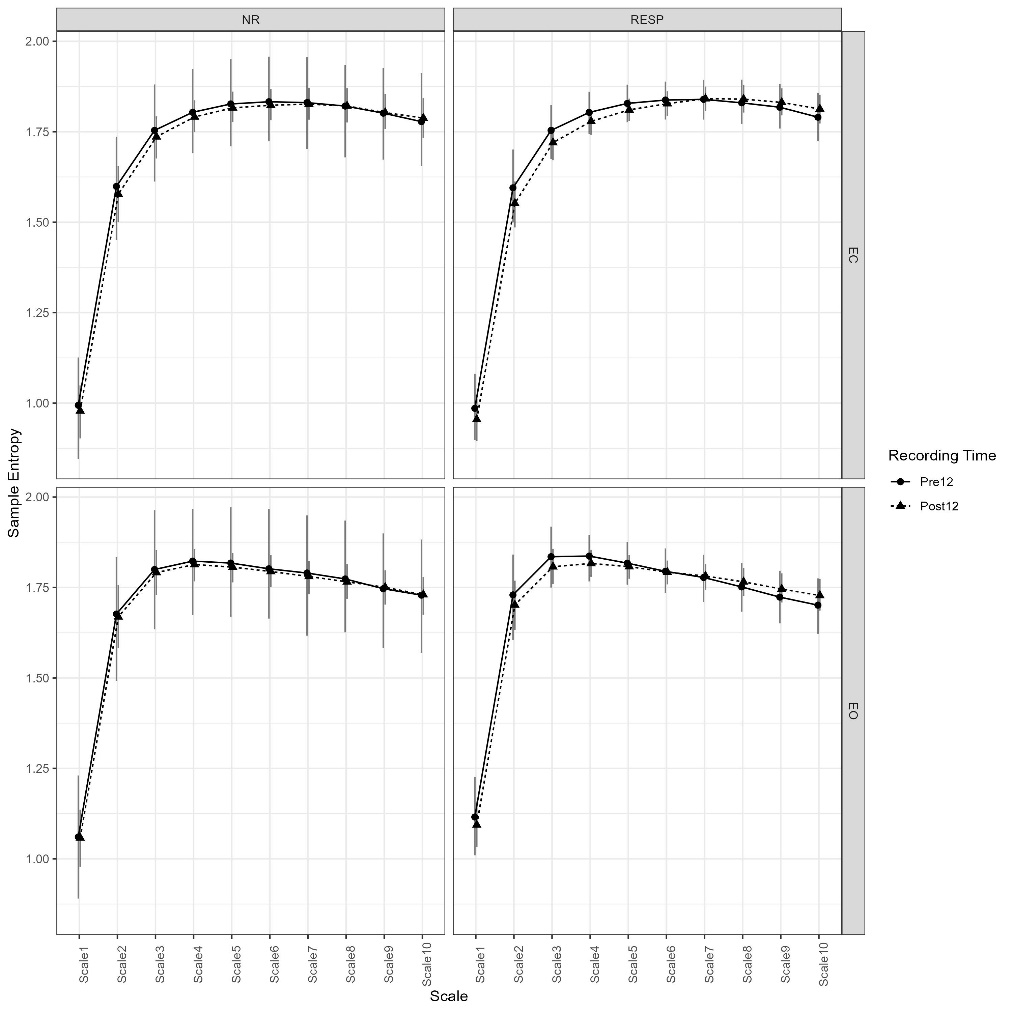


Figure S21b. Estimated marginal means for Multi-Scale Entropy across response status and scale by task and recording time. Note: Bars represent 95% Highest Posterior Density; Baseline, BAS; post-treatment, POST; follow-up, FUP; Before 12pm, Pre12; After 12pm, Post12; Eyes Closed, EC; Eyes Open, EO; NR, Non-Responder; RESP, Responder. Response Measures, resp.meas.


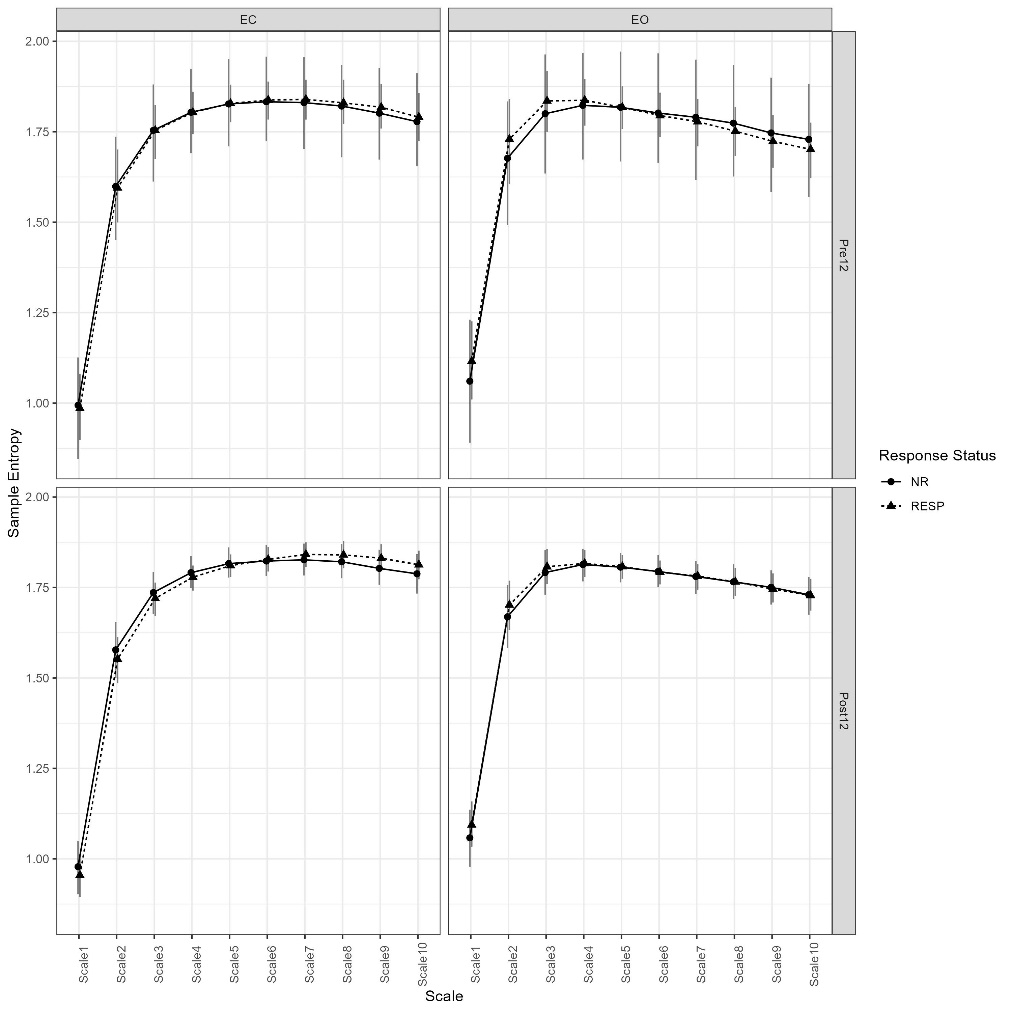

Supplement: Supplementary file 1 — Supporting Information [file BRB3-14-e70166-s001.docx]
